# Exploring molecular targets: herbal isolates in cervical cancer therapy

**DOI:** 10.1186/s44342-024-00008-1

**Published:** 2024-06-26

**Authors:** Maryam Ahmadi, Razieh Abdollahi, Marzieh Otogara, Amir Taherkhani

**Affiliations:** 1 Clinical Research Development Unit of Fatemiyeh Hospital, Department of Gynecology, School of Medicine, Hamadan University of Medical Sciences, Hamadan, Iran; 2 Mother and Child Care Research Center, Hamadan University of Medical Sciences, Hamadan, Iran; 3 Research Center for Molecular Medicine, Hamadan University of Medical Sciences, Hamadan, Iran

**Keywords:** Cervical cancer, Drug, Pathogenesis, Pathway, Prognosis, Target

## Abstract

**Objective:**

Cervical cancer (CxCa) stands as a significant global health challenge, ranking fourth in cancer-related mortality among the female population. While chemotherapy regimens have demonstrated incremental progress in extending overall survival, the outlook for recurrent CxCa patients remains disheartening. An imperative necessity arises to delve into innovative therapeutic avenues, with molecular targeted therapy emerging as a promising candidate. Previous investigations have shed light on the therapeutic effectiveness of five distinct herbal compounds, epicatechin, curcumin, myricetin, jatrorrhizine, and arborinine, within the context of CxCa.

**Methods:**

A systems biology approach was employed to discern differentially expressed genes (DEGs) in CxCa tissues relative to healthy cervical epithelial tissues. A protein–protein interaction network (PPIN) was constructed, anchored in the genes related to CxCa. The central genes were discerned within the PPIN, and Kaplan–Meier survival curves explored their prognostic significance. An assessment of the binding affinity of the selected herbal compounds to the master regulator of prognostic markers in CxCa was conducted.

**Results:**

A significant correlation between the overexpression of MYC, IL6, JUN, RRM2, and VEGFA and an adverse prognosis in CxCa was indicated. The regulation of these markers is notably influenced by the transcription factor CEBPD. Molecular docking analysis indicated that the binding affinity between myricetin and the CEBPD DNA binding site was robust.

**Conclusion:**

The findings presented herein have unveiled pivotal genes and pathways that play a central role in the malignant transformation of CxCa. CEBPD has emerged as a potential target for harnessing the therapeutic potential of myricetin in this context.

**Supplementary Information:**

The online version contains supplementary material available at 10.1186/s44342-024-00008-1.

## Introduction

Cervical cancer (CxCa) is ranked fourteenth among all cancers and the fourth-ranked cancer among women worldwide [1]. The prevalence of cervical cancer remains significant globally, with an estimated 604,127 cases and 341,831 deaths in 2020 [2]. The age-standardized incidence rate was 13.3 cases per 100,000 women-years, and the mortality rate was 7.2 deaths per 100,000 women-years. There are substantial geographical and socioeconomic inequalities in cervical cancer incidence and mortality, with higher rates in countries with lower levels of human development [2]. In the UK, there has been a decline in cervical cancer incidence due to the national human papillomavirus (HPV) immunization program and cervical cancer screening service. Still, disparities in screening rates and early diagnosis persist [3]. The 5-year survival rates range from 50 to 70%, and the prognosis of patients with cervical cancer varies depending on factors such as stage, histology, age, and treatment modalities. Older patients with squamous cell carcinoma have shown better survival rates. Targeted therapy and immunotherapy demonstrate promise in improving outcomes for advanced and metastatic cervical cancer, but further research is needed [4–9].

Accumulating evidence indicates that several herbs have anticancer effects in cervical cancer. In a study by Nayim et al. [10], the methanolic root extract of *Imperata cylindrica* (IC) inhibited the proliferation of HeLa and CaSki cells and induced apoptosis and cell cycle arrest in the G0/G1 phase. The extract contained compounds with reported anticancer activity, including epicatechin, curcumin, myricetin, and jatrorrhizine. Furthermore, Piboonprai et al. [11] isolated arborinine, a major acridone alkaloid from the ethyl acetate extract of *Glycosmis parva* (G. parva) leaves, and demonstrated its anticancer effects in cervical cancer cells (HeLa). Arborinine decreased cell proliferation, induced apoptosis through caspase-dependent mechanisms, and suppressed cancer cell migration and invasion.

In this context, it was postulated that epicatechin, curcumin, myricetin, jatrorrhizine, and arborinine could potentially target crucial genes implicated in the development of cervical cancer. Consequently, a comprehensive bioinformatics study was undertaken to pinpoint the prognostic indicators associated with adverse outcomes in individuals diagnosed with cervical cancer. Subsequently, a gene regulatory network (GRN) was formulated to elucidate the upstream regulators governing these prognostic markers. The primary transcription factor (TF) identified as a potential target for the aforementioned herbal compounds was assessed. Subsequently, the binding affinity of epicatechin, curcumin, myricetin, jatrorrhizine, and arborinine to the binding site of this potential target was evaluated utilizing the AutoDock tool. Hence, the current study encompassed two distinct phases: (1) a systems biology investigation aimed at identifying potential biomarkers linked to an unfavorable prognosis in cervical cancer patients and (2) a structural bioinformatics analysis conducted to delineate the binding affinities between the upstream regulator responsible for transcription of negative markers in cervical cancer and the five active components. In the context of this study, the term “negative markers” denotes genes characterized by overexpression, which exhibits a correlation with unfavorable prognosis among cancer patients [12]. The systems biology component of the study involved re-examining the gene expression profile dataset GSE63514, originally established by Den Boon et al. [13]. This re-analysis was conducted to discern differentially expressed genes (DEGs) within the context of cervical squamous epithelial cancer in comparison with the gene expression profile of normal cervical epithelium.

## Materials and methods

### Study design

Den Boon et al. [13] diligently recruited female participants into the Study to Understand Cervical Cancer Early Endpoints and Determinants (SUCCEED) with the explicit approval and informed consent granted by the Human Subject Research Institutional Review Boards at the University of Wisconsin–Madison, the National Cancer Institute, and the University of Oklahoma Health Sciences Center. Cervical samples obtained through the SUCCEED initiative were subjected to a cryosectioning process, creating a series of sections measuring 10 to 12 µm thick. Subsequently, the PixCell II Laser Capture Microdissection System was employed to capture the epithelial lining of the cervix meticulously. This process involved the selection of specimens from both normal healthy controls and those with precancerous or invasive cancerous cell masses within cervical lesions. RNA extraction was carried out in adherence to the manufacturer’s guidelines (Invitrogen) using TRIzol. The extracted RNA was subsequently utilized to generate double-stranded cDNA featuring a T7 RNA polymerase promoter-linked, oligo (dT)-primed structure. This cDNA, in turn, served as a template for synthesizing T7 transcripts that were complementary to both human and viral mRNAs, collectively referred to as cRNA. The T7 transcripts underwent a secondary cycle of cDNA synthesis and amplification, facilitated using T7 RNA polymerase. Out of the 227 processed specimens, 128 epithelial RNA extracts, comprising 24 from normal samples, 76 from cervical intraepithelial neoplasia samples, and 28 from cancer specimens, provided cRNA of exceptional quality, suitable for subsequent Affymetrix U133 Plus 2.0 microarray analysis.

### Dataset recovery

In this study, the gene expression dataset GSE63514 [13] was utilized for re-analysis, accessible through the following link: http://www.ncbi.nlm.nih.gov/geo. This dataset was originally generated using the GPL570 [HG-U133_Plus_2] Affymetrix Human Genome U133 Plus 2.0 Array, serving as the foundational platform for our re-analysis. The DEGs in cervical cancerous tissues (*n* = 28) were discerned in comparison with normal cervical epithelia (*n* = 24), employing the criteria of an adjusted *p*-value less than 0.01 and an absolute Log2 fold change (FC) exceeding 1. Additionally, the volcano plot of the dataset GSE63514 was presented through the Shiny server, accessible via https://huygens.science.uva.nl/ [14].

### Networking and functional analysis

To augment the identification of genes relevant to cervical cancer, we incorporated the DisGeNET database (https://www.disgenet.org/ [15]). Our methodology involved cross-referencing the DEGs identified from the GEO dataset (GSE63514) with the gene-disease associations cataloged in DisGeNET. This approach aimed to comprehensively capture a broader spectrum of genes pertinent to cervical cancer.

To unveil pivotal genes associated with cervical cancer, particularly those that have been previously reported, version 7.0 of the DisGeNET database was employed. In pursuit of this, the dataset bearing the ID C0279671 was obtained and downloaded. The genes in this dataset were subsequently integrated with the DEGs identified in the current study, culminating in assembling a comprehensive set of genes intricately linked to cervical cancer.

Next, the interactions among the set of genes were inferred utilizing the STRING version 12.0 knowledge base, which can be accessed at http://string-db.org [16]. Furthermore, unconnected proteins were systematically removed from the network to refine the analysis [17]. Subsequently, the Cytoscape 3.10.1 software, accessed at https://cytoscape.org/ [17], was employed to visualize the PPIN and compute the nodes’ centrality within this protein graph. Building upon the methodology established by Jeong et al. [18], which underscored a positive correlation between a protein’s degree within the PPIN and its essentiality, we have delineated hub genes as those demonstrating a degree exceeding twice the network’s average degree, in conjunction with betweenness and closeness centralities surpassing the network’s average. These hub genes were further scrutinized to assess their potential significance in shaping the prognosis of patients affected by cervical cancer (CxCa). To delve deeper into the pathways and biological processes orchestrating the malignant transition from normal cervical epithelia to a cancerous state, modules within the PPIN were unearthed using the MCODE (Molecular Complex Detection) plugin [19]. Significant modules, characterized by the following features, were regarded as noteworthy and chosen for subsequent analysis of pathways and biological processes: (1) an MCODE score exceeding 3 and (2) a gene count surpassing 10 [20]. Examining noteworthy pathways and biological processes influenced by the clusters was executed by utilizing the g:Profiler tool, accessible at https://biit.cs.ut.ee/gprofiler/gost [21]. Significance was assessed by adopting a defined cutoff criterion, necessitating a false discovery rate (FDR) below 0.05 and a prerequisite for a minimum of 10 enriched genes within each specific term.

### Kaplan–Meier and boxplot analyses

The prognostic impact of the hub genes in cervical cancer was assessed through the generation of Kaplan–Meier curves, a task facilitated by the GEPIA2 database, which can be accessed at http://gepia2.cancer-pku.cn/#survival [22]. The prognostic significance of the genes was determined by applying the log-rank test, and genes with a hazard ratio (HR) *p*-value less than 0.05 were deemed to possess significant prognostic relevance. The GEPIA2 harnesses the analytical capabilities to scrutinize RNA sequencing data sourced from the Cancer Genome Atlas [23] and the Genotype-Tissue Expression [24] databases, thereby yielding robust and dependable outcomes for the assessment of survival and box plot analyses, especially when comparing cancer patients to their healthy counterparts. Furthermore, the expression patterns of prognostic markers within CxCa tissues and healthy control samples were thoroughly assessed using pertinent data from the GEPIA2 database.

### Gene regulatory network

The present study aimed to identify upstream regulators mediating the transcription of prognostic markers associated with poor prognosis in patients with CxCa. Therefore, the iRegulon plugin within the Cytoscape was employed for the possible detection of transcription factors responsible for the regulation of hub genes. iRegulon calculates the normalized enrichment score (NES) for each regulator, and those with NES > 5 were considered significant [25].

### Consensus sequences logo and matching score calculation

The consensus sequence logo for the binding site of the transcription factor, responsible for regulating all negative markers in cervical cancer within this study, was provided through the utilization of the JASPAR database (https://jaspar.genereg.net/) [26]. Subsequently, the calculation of the total match score for the consensus sequence of the transcription factor was manually performed, employing R programming (version 4.0.0) [27], in alignment with the methodology outlined by Xiong [28]. This score is interpretable as the probability of the consensus sequence aligning with the transcription factor’s binding site, with a possibility of being two times more likely than that arising by random chance. Our previously published work can include additional, comprehensive details regarding this methodology [17].

### Molecular docking analysis

A Windows-based PC with the following features was used for molecular docking analyses: system type, 64-bit processor, Intel Core i7, and installed memory 32 GB. A possible target for epicatechin, curcumin, myricetin, jatrorrhizine, and arborinine was assigned to the upstream transcription factor that plays a significant role in the transcription of negative markers in CxCa patients. The energy minimizing of the protein was employed on the receptor using Swiss-pdbViewer version 4.1.0, which can be accessed at https://spdbv.unil.ch [29]. The structures of the ligands were initially obtained in SDF (Structure-Data File) format and subsequently converted into PDF (Portable Document Format) files before undergoing the energy minimization process [30–32]. Kollmann charges and polar hydrogens were incorporated into the protein, and local charges and rotational motion parameters were applied to the ligands. Ultimately, the PDBQT files for the receptor and ligands were constructed utilizing the MGL tools [33].

### Post-docking analysis

The Gibbs free energy of binding (Δ*G*_binding_) between the investigated herbal compounds and the receptor was determined through the utilization of the AutoDock 4.0 software. For each ligand, a total of 50 independent runs were configured. Subsequently, the most negative value of Δ*G*_binding_, as observed in the root mean square deviation (RMSD) table, was documented as the binding energy between the ligand and the receptor [33]. To elucidate the interactions between the receptor’s DNA binding site and the active compounds, the BIOVIA Discovery Studio Visualizer version 19.1.0.18287 was employed.

## Results

### Critical genes mediating cervical cancer

Comprehensive demographic information concerning patients, encompassing factors such as race, ethnicity, income, marital status, history of pregnancy, age at sexual debut, smoking status, and BMI (kg/m^2^), among others, is available in the original publication [34].

**Fig. 1:**
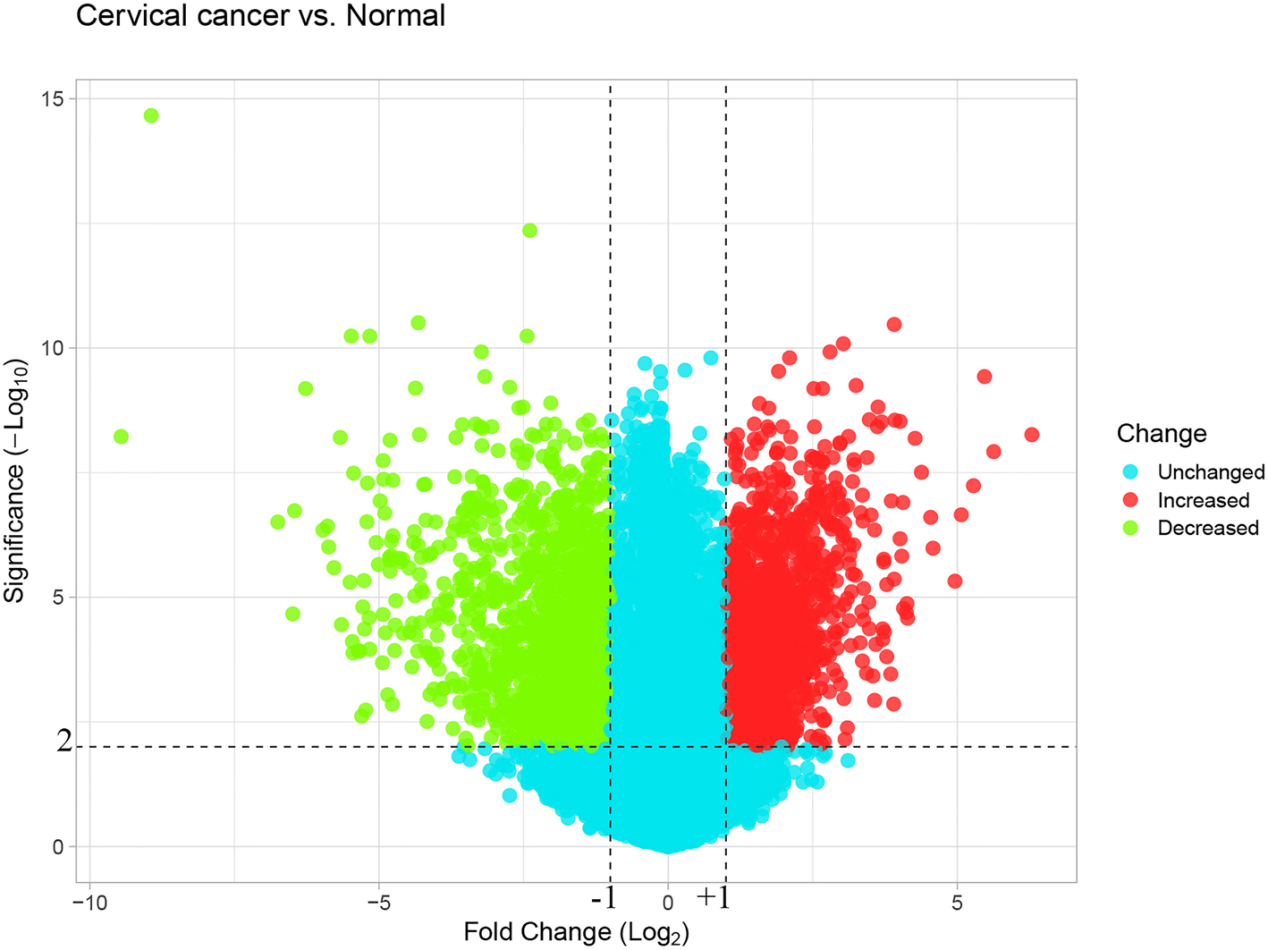
The volcano plot illustrating the differential gene expression profiles within cervical cancer tissues when juxtaposed with those originating from healthy epithelial samples

The application of GEO2R yielded a notable distinction in cervical cancer observations compared to those derived from normal cervical epithelia. A comprehensive set of 801 DEGs, meeting the stringent criteria of a *p*-value less than 0.01 and an absolute Log2 FC greater than 1, were successfully pinpointed within the context of the compared groups (as detailed in Additional file 2: Table S1). The outcome of this analysis is vividly depicted in Fig. 1, showcasing a volcano plot elucidating the dataset GSE63514.

**Fig. 2:**
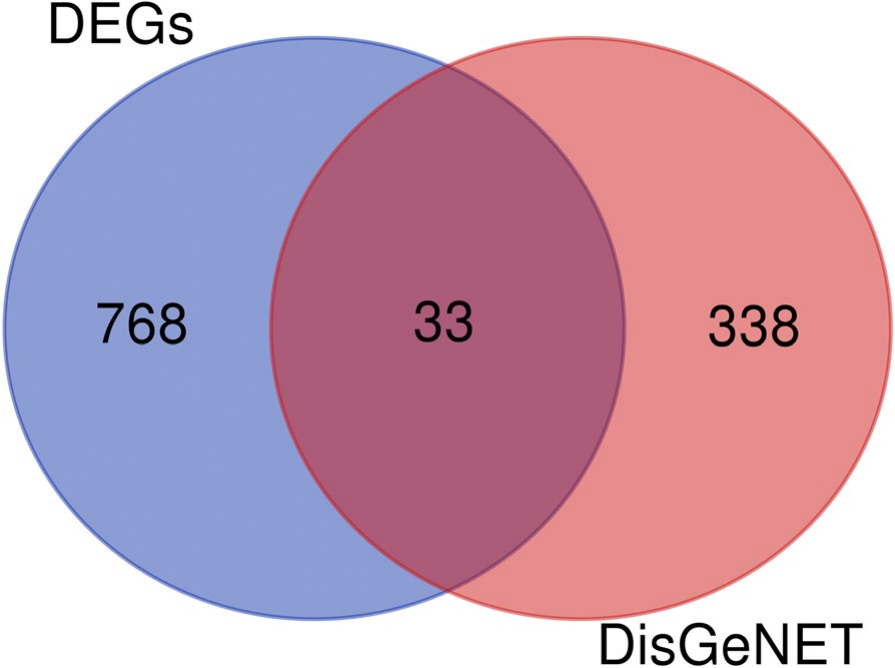
The Venn diagram illustrating the number of common genes between the differentially expressed gene dataset and cervical cancer-related genes sourced from the DisGeNET database

Renowned as a discovery platform, DisGeNET hosts 1 of the most extensive collections of genes and variants associated with human diseases, publicly accessible. This database seamlessly integrates data from expert-curated repositories, GWAS catalogs, findings from animal models, and scientific literature. Notably, DisGeNET ensures uniform annotation of data with controlled vocabularies and community-driven ontologies. Moreover, it provides diverse original metrics to aid in prioritizing genotype–phenotype relationships. An exhaustive exploration of the DisGeNET database led to the identification of 371 genes intricately associated with cervical cancer, as documented in Additional file 3: Table S2. In the culmination of these findings, the DEGs and DisGeNET-derived genes were methodically integrated, yielding a meticulously curated list of 1139 distinct genes. Notably, 33 genes were found to be common between the 2 datasets, underscoring their intimate association with cervical cancer pathogenesis (Fig. 2).

### PPIN and gene set enrichment analyses

The PPIN encompasses both physical protein–protein interactions and functional associations derived from a multitude of sources, including experimental data, curated databases, co-expression data, and text mining from scientific literature. Each interaction is assigned a score ranging from 0 to 1, with higher values indicative of greater confidence in the interaction [35]. To visually depict the interrelationships among genes associated with CxCa, the network was graphically represented, adhering to a stringent confidence score threshold of 0.7 or higher. This threshold, determined through the utilization of the STRING database, ensures the reliability and biological relevance of the interactions incorporated within the network.

Nodes lacking connections were systematically removed from the graph, resulting in Cytoscape rendering the PPIN comprising 758 vertices and 8585 edges. In the context of PPIN, degree centrality denotes the count of direct connections associated with a node (gene/protein) within the network. Conversely, betweenness centrality assesses the frequency with which a node occupies positions on the shortest paths between pairs of other nodes, while closeness centrality measures the proximity of a node to all other nodes [36]. A topological analysis unveiled 57 nodes exhibiting a degree exceeding twice the average while also demonstrating betweenness and closeness values surpassing the average node metrics. Consequently, these nodes were designated hub proteins within the PPIN linked to the malignant transformation in cervical cancer, as outlined in Table 1. The mean values for degree, betweenness, and closeness centrality were 22.65, 0.006, and 0.3423, respectively.

**Table 1:** A total of 57 genes linked to cervical cancer pathogenesis were assigned as hubs in this research

Name	Degree	Betweenness	Closeness
CDK1	171	0.051	0.440
CCNA2	151	0.024	0.426
CCNB1	145	0.025	0.423
TP53	144	0.153	0.482
TOP2A	143	0.021	0.423
BUB1	143	0.010	0.405
NCAPG	138	0.010	0.381
BUB1B	127	0.010	0.404
AURKB	125	0.009	0.402
CDC6	122	0.009	0.390
RRM2	122	0.008	0.403
CDC20	119	0.007	0.387
ASPM	118	0.006	0.374
NDC80	117	0.011	0.372
CHEK1	116	0.010	0.406
AURKA	114	0.014	0.408
EXO1	114	0.010	0.398
RFC4	110	0.007	0.397
BIRC5	107	0.031	0.419
CENPF	100	0.006	0.365
CEP55	97	0.007	0.366
PCNA	95	0.015	0.394
RAD51	94	0.013	0.396
MCM5	93	0.010	0.384
TYMS	91	0.009	0.369
STAT3	90	0.038	0.434
EGFR	89	0.085	0.447
MKI67	89	0.007	0.398
AKT1	86	0.050	0.446
HMMR	83	0.013	0.389
FOXM1	81	0.007	0.396
CTNNB1	75	0.037	0.431
MYC	73	0.025	0.437
RFC3	71	0.008	0.381
MAPK3	68	0.033	0.427
IL6	62	0.022	0.392
CCND1	62	0.016	0.430
ACTB	61	0.044	0.438
HRAS	61	0.022	0.395
CDC25C	61	0.007	0.396
ESR1	58	0.025	0.419
TNF	58	0.023	0.393
VEGFA	57	0.011	0.395
RPA3	57	0.008	0.373
STAT1	55	0.030	0.401
JUN	55	0.019	0.421
PIK3CA	55	0.014	0.391
ERBB2	53	0.025	0.423
PTEN	52	0.019	0.413
CASP3	52	0.017	0.429
MAPK1	52	0.010	0.396
CDH1	50	0.014	0.396
CD44	49	0.023	0.407
HIF1A	47	0.013	0.417
E2F1	47	0.012	0.396
MDM2	46	0.013	0.411
CDKN2A	46	0.010	0.403

**Fig. 3:**
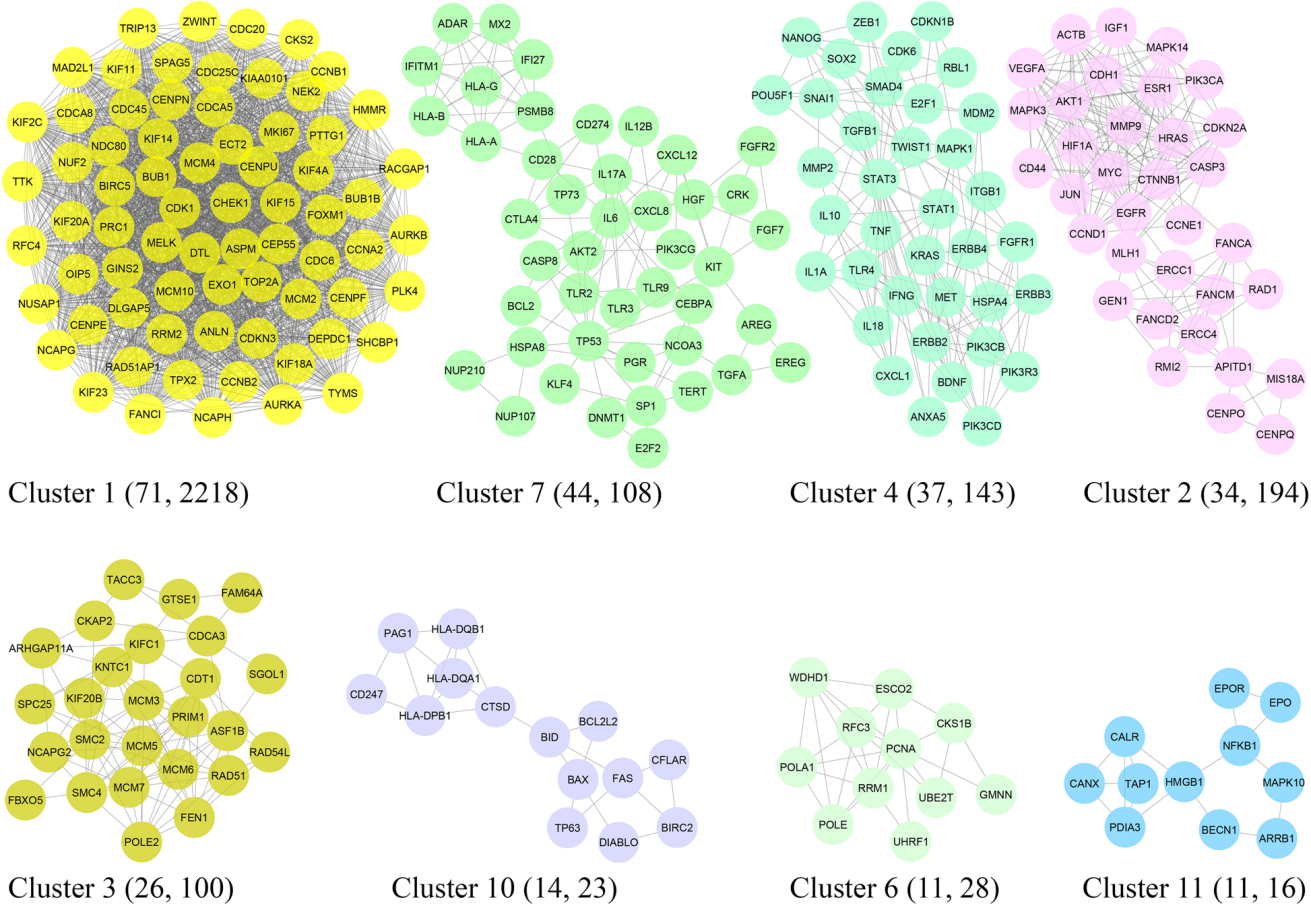
Protein–protein interaction network analysis. The MCODE plugin revealed eight significant modules from the network associated with cervical cancer. MCODE, molecular complex detection

**Fig. 4:**
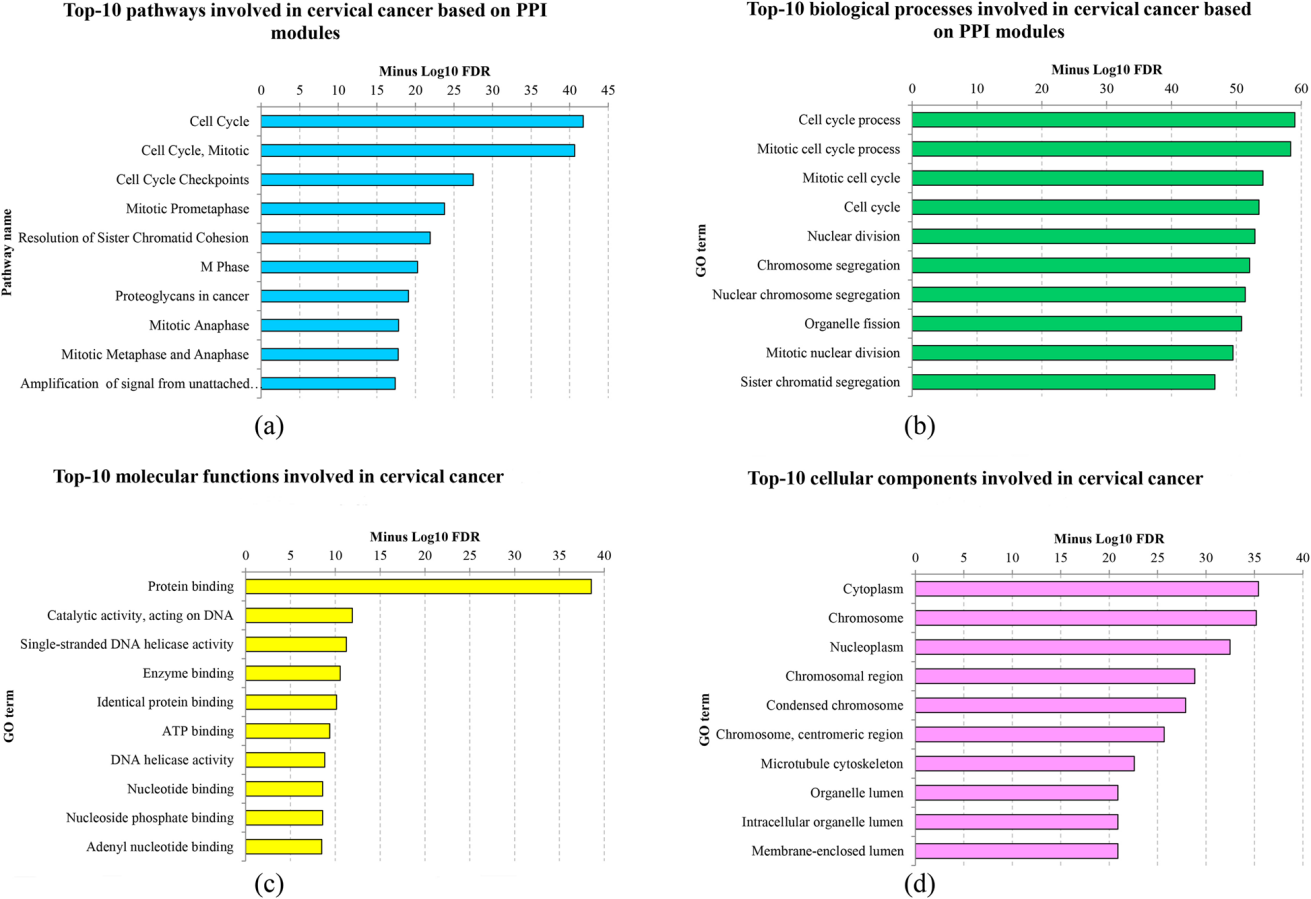
The top-ranked pathways (**a**), biological processes (**b**), molecular functions (**c**), and cellular components (**d**) that exhibit significant enrichment in cervical cancer, as determined by false discovery rate (FDR). The *X*-axis indicates the name of the respective term, while the *Y*-axis portrays the − log10 FDR, providing a graphical representation of the statistical significance of these enrichments. FDR, false discovery rate

The MCODE algorithm [37] is instrumental in detecting densely connected regions within the PPIN, potentially denoting molecular complexes or functional modules. The MCODE score serves as a quantitative measure, indicating the likelihood of a node’s affiliation with a densely connected subgraph. Higher scores correlate with an increased probability of node involvement in a functional cluster or complex. Nodes exhibiting elevated MCODE scores underwent additional scrutiny to elucidate potential functional modules or pathways pertinent to cervical cancer pathogenesis. Within the PPIN, eight distinct modules were discerned, each containing more than ten genes and boasting an MCODE score exceeding three. These clusters are labeled as nos. 1, 2, 3, 4, 6, 7, 10, and 11 (Fig. 3). The GSEA was systematically conducted on these clusters to unveil the pathways and biological processes that exhibited significant enrichment in the context of cervical cancer. The comprehensive catalog of pathways and biological processes is comprehensively documented in Additional files 4 and 5: Tables S3 and S4, respectively. Nonetheless, the top ten terms are visually depicted in Fig. 4a, b for a concise overview. In addition to pathways and biological processes associated with cell cycle and mitotic division, it is noteworthy that the “proteoglycans in cancer” pathway (KEGG: 05205) exhibited significant enrichment in the context of cervical cancer progression.

A comprehensive set of 1139 unique genes was input into g:Profiler to elucidate the molecular functions and cellular components that experience significant perturbations in cervical cancer. Subsequently, the analysis revealed that “protein binding” (GO: 0005488) emerged as the most notably enriched molecular function in cervical cancer, while “cytoplasm” (GO: 0005737) stood out as the foremost cellular component that undergoes significant dysregulation in CxCa. Figure 4c, d visually depicts cervical cancer’s top 10 enriched molecular functions and cellular features, respectively. For a more extensive list, please refer to Additional files 6 and 7: Tables S5 and S6, which provide a comprehensive catalog of these enriched functions and components.

### Survival and expression analyses

The dataset employed for this analysis was specifically tailored to “cervical squamous cell carcinoma and endocervical adenocarcinoma” (CESC), representing one of the cancer types accessible within the GEPIA2 database. The Kaplan–Meier survival curves elucidated a statistically significant association between the upregulation of five genes, specifically MYC, IL6, JUN, RRM2, and VEGFA, and an unfavorable clinical outcome in individuals afflicted by CxCa. MYC, IL6, and JUN were identified as significant factors in the DisGeNET database. RRM2 was among the DEGs, whereas VEGFA was also noted as a DEG and was part of the DisGeNET database. Moreover, RRM2 was associated with cluster no. 1 and IL6 with cluster no. 7. Conversely, MYC, JUN, and VEGFA were linked to cluster no. 2. Moreover, the overexpression of RFC4, EXO1, PCNA, TOP2A, and TYMS demonstrated a correlation with a more favorable prognosis in patients diagnosed with cervical cancer, as determined through log-rank test and hazard ratio (HR) *p*-values of less than 0.05 (refer to Table 2 and Additional file 1: Fig. S1).

**Table 2:** A total of ten genes were found to be prognostic markers in patients with CxCa

A. Single gene			
**Gene symbol (gene label)**	**HR (high)**	***P* (log-rank test)**	***P* (HR)**
MYC (A)	2	0.0039	0.0046
IL6 (B)	1.7	0.024	0.026
JUN (C)	1.7	0.027	0.029
RRM2 (D)	1.7	0.029	0.031
VEGFA (E)	1.7	0.032	0.035
RFC4 (F)	0.56	0.014	0.015
EXO1 (G)	0.57	0.018	0.019
PCNA (H)	0.58	0.022	0.023
TOP2A (I)	0.59	0.029	0.031
TYMS (J)	0.62	0.041	0.044
**B. Combination of genes**			
**Prognostic panel**	**HR (high)**	***P* (log-rank test)**	***P* (HR)**
A + B	1.4	0.14	0.14
A + B + C	1.9	0.029	0.031
A + B + C + D	1.7	0.023	0.024
A + B + C + D + E	1.9	0.0068	0.0079
F + G	0.65	0.069	0.079
F + G + H	0.59	0.028	0.03
F + G + H + I	0.59	0.027	0.029
F + G + H + I + J	0.53	0.0083	0.0094

*CxCa* cervical cancer, *HR* hazard ratio

**Fig. 5:**
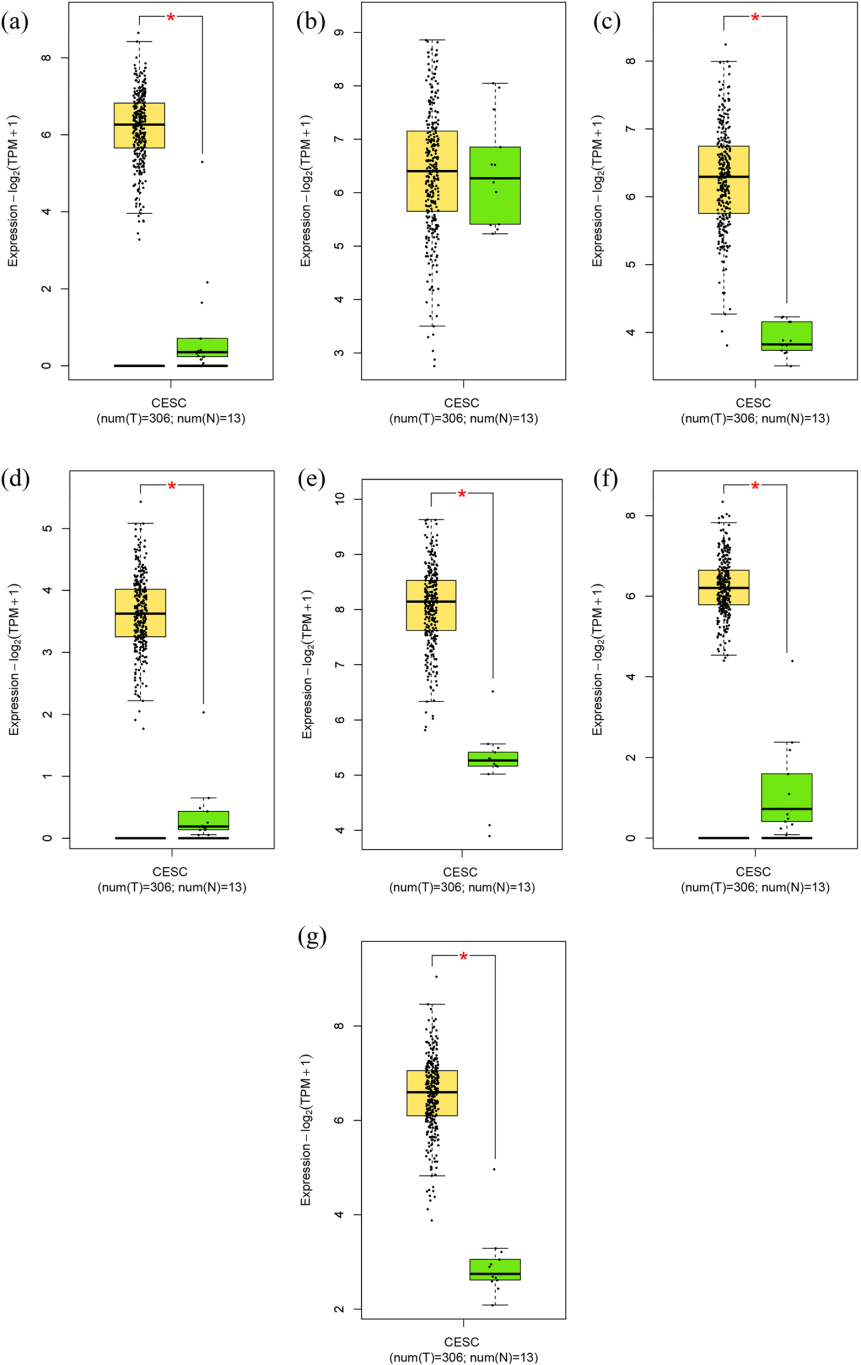
The boxplot analysis, conducted utilizing data from the GEPIA2 database, offers insights into the gene expression patterns of prognostic markers in CxCa. This analysis used a dataset comprising 306 CxCa samples (highlighted in yellow) and 13 normal tissues (depicted in green). These findings underscore these markers’ differential gene expression, accentuating their potential role in CxCa, with CxCa samples consistently showing elevated expression levels compared to normal tissues. RRM2 (**a**). VEGFA (**b**). RFC4 (**c**). EXO1 (**d**). PCNA (**e**). TOP2A (**f**). TYMS (**g**). CxCa, cervical cancer

Among the ten markers that indicated poor or favorable prognosis in cervical cancer patients, this study highlighted RRM2, VEGFA, RFC4, EXO1, PCNA, TOP2A, and TYMS as DEGs. To authenticate the distinct expression profiles of RRM2, VEGFA, RFC4, EXO1, PCNA, TOP2A, and TYMS in cervical cancer, the GEPIA2 server was employed. Specifically, an analysis was conducted using the CESC dataset, which forms part of the TCGA data accessible within GEPIA2. Boxplot visualizations were generated to juxtapose the mRNA expression levels of these genes in cervical cancer tissues (tumor samples) and normal cervical epithelial tissues (normal samples). The outcomes of this assessment substantiated our preliminary observations, revealing the upregulation of RRM2 and VEGFA, alongside the downregulation of RFC4, EXO1, PCNA, TOP2A, and TYMS in cervical cancer tissues relative to normal cervical epithelial tissues (Fig. 5).

### GRN and consensus sequence logo

**Fig. 6:**
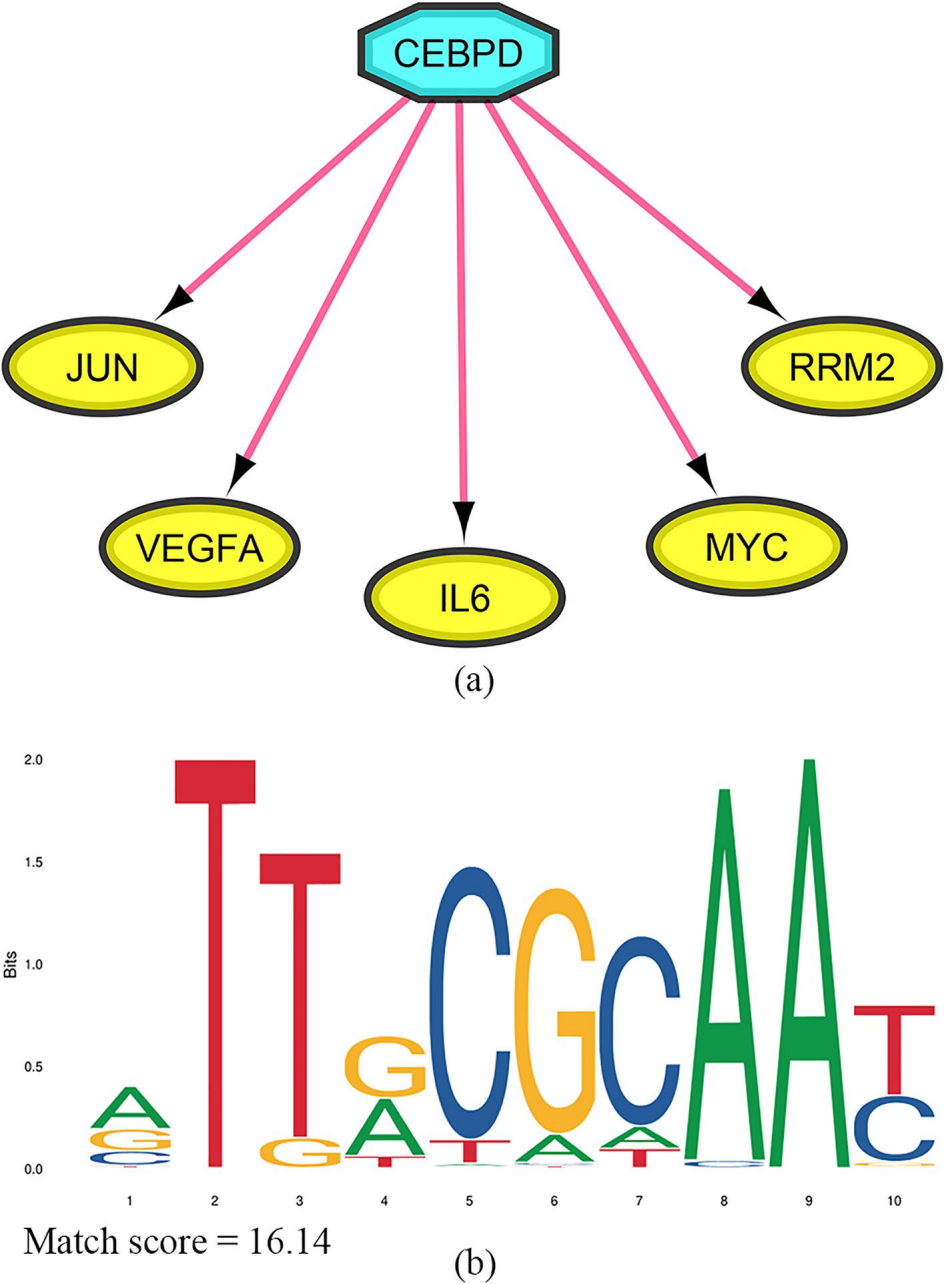
**a** A regulatory motif involving CEBPD acting as the upstream regulator for five negative markers in cervical cancer. **b** The consensus logo represents the CEBPD binding site. CEBPD, CCAAT/enhancer-binding protein beta

In the pursuit of identifying master regulators responsible for orchestrating the transcription of markers associated with an adverse prognosis in cervical cancer patients, a rigorous analysis was conducted. The NES serves as a vital statistical metric within gene set enrichment analysis (GSEA), offering a means to evaluate the overrepresentation of genes associated with a specific biological pathway or set within a ranked gene list. It is essential to note that there is not a universally applicable threshold for NES. While a commonly utilized starting point is |NES|> 1 to identify potentially significant enrichments [38], the precise interpretation hinges upon the specific research inquiry and disciplinary context of the study. Herein, we opted for a more stringent threshold, requiring an NES exceeding 5 for transcription factors (Table 3). This decision aimed to prioritize those transcription factors deemed more likely to hold functional relevance within our system. The most noteworthy discovery in this regard was the transcription factor H1FX, which showed an exceptionally significant NES score of 7.167. H1FX was found to exert its regulatory influence over four key markers, namely RRM2, MYC, VEGFA, and JUN. The second most significant result was also attributed to CEBPD, with an NES value of 5.972. Notably, this transcription factor exhibited substantial interactions with all five markers associated with a negative prognosis in CxCa (Fig. 6a). Consequently, CEBPD was identified as a potential target for therapeutic interventions involving epicatechin, curcumin, myricetin, jatrorrhizine, and arborinine, indicating its potential role in mitigating the adverse outcomes linked to CxCa.

**Table 3:** A total of ten transcription factors were identified as upstream regulators of negative markers in CxCa

Transcription factor	NES	Targets
H1FX	7.167	RRM2, MYC, VEGFA, JUN
CEBPD	5.972	RRM2, MYC, VEGFA, JUN, IL6
SIN3A	5.615	RRM2, MYC, VEGFA, JUN
JAZF1	5.587	MYC, VEGFA, JUN, IL6
JUND	5.452	MYC, VEGFA, JUN, IL6
LEF1	5.307	MYC, VEGFA, IL6
RDBP	5.277	RRM2, MYC, JUN
ZBTB7A	5.171	RRM2, MYC, VEGFA
HIF1A	5.035	RRM2, MYC, VEGFA, IL6
ATF4	5.008	VEGFA, JUN, IL6

*CxCa* cervical cancer

A comprehensive investigation of CEBPD was conducted using the JASPAR webserver. Within this analysis, the consensus sequence logo for this transcription factor was readily accessible within the database. The match score for the consensus sequence of CEBPD was meticulously calculated and yielded a score of 16.14, as illustrated in Fig. 6b. This score signifies the similarity or correspondence between the identified consensus sequence and the known binding pattern for CEBPD, reinforcing its significance in the regulatory network associated with CxCa.

### Molecular docking and post-docking analyses

The three-dimensional configuration of CEBPD proved unattainable within the RCSB database (https://www.rcsb.org) [39]. As a result, a homology modeling approach was pursued utilizing the SWISS-MODEL web server, accessible at https://swissmodel.expasy.org/ [40]. In this endeavor, the 6mg3.1.A CCAAT/enhancer-binding protein beta structure was employed as a template for modeling, bearing a sequence identity of 62.67%.

The CASTp server (Computed Atlas of Surface Topography of proteins), which can be accessed at http://sts.bioe.uic.edu/castp/index.html?201l, was utilized to analyze and predict potential binding sites and interacting residues within the CEBPD protein structure [41]. This analysis leveraged the three-dimensional shape and surface topography of the protein. Its primary objective was to identify the key residues within the DNA binding site of CEBPD, as these residues are expected to play a crucial role in facilitating the binding of CEBPD to its target DNA sequences. The interacting residues within the CEBPD DNA binding site, as determined by the CASTp server, include Arg209, Ala212, Lys213, Arg215, Asn216, and Gln217.

A smaller Δ*G*_binding_ value corresponds to a heightened binding affinity between ligands and receptors. It has been experimentally ascertained that when Δ*G*_binding_ falls below − 7.00 kcal/mol, it signifies a robust binding affinity between a ligand and a receptor [35]. In this context, the calculated Δ*G*_binding_ values for myricitin and curcumin with the CEBPD DNA binding site were observed to be − 8.44 and − 7.11 kcal/mol, respectively. This substantiates the classification of these herbal compounds as potential inhibitors of CEBPD. Consequently, the inhibition of CEBPD is postulated to play a pivotal role in the therapeutic effects of myricitin and curcumin in patients afflicted with CxCa. Comprehensive information regarding the various energy components between CEBPD and the examined ligands can be found in Table 4.

**Table 4:** Various categories of energy parameters and *K*i values about the interactions between CEBPD and the ligands were computed employing the AutoDock 4.0 tool

PubChem ID	Name	Free binding energy	Intermolecular energy	Internal energy	Torsional free energy	Unbound system’s energy	*K*i
5,281,672	Myricetin	− 8.44	− 7.04	− 4.87	2.39	− 1.09	650.77 nM
969,516	Curcumin	− 7.11	− 8.48	− 2.97	3.58	− 0.76	6.12 µM
1203	Epicatechin	− 6.53	− 6.62	− 2.29	1.79	− 0.59	16.46 µM
72,323	Jatrorrhizine	− 6.13	− 6.91	0.81	1.19	− 0.41	32.36 µM
5,281,832	Arborinine	− 5.3	− 5.86	− 0.85	0.89	− 0.52	130.99 µM

*Ki* inhibition constant, *CEBPD* CCAAT/enhancer-binding protein beta

**Fig. 7:**
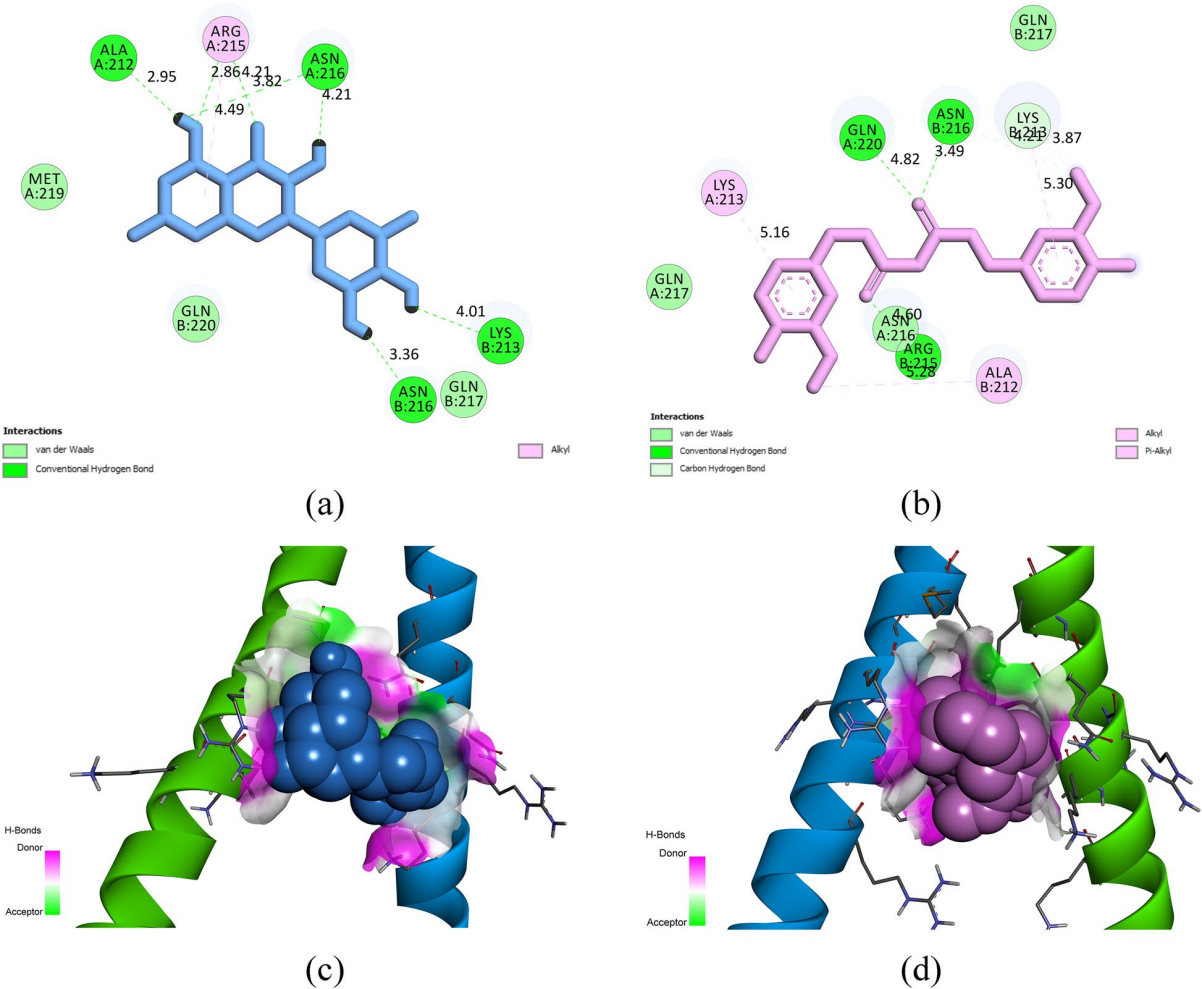
Two-dimensional arrangement of myricetin (**a**) and curcumin (**b**) within the CEBPD DNA binding site. Three-dimensional configuration of myricetin (**c**) and curcumin (**d**) within the CEBPD DNA binding site. CEBPD, CCAAT/enhancer-binding protein beta

The post-docking analysis revealed that myricetin exhibited seven hydrogen bonds and one hydrophobic interaction with CEBPD. Further, curcumin formed five hydrogen bonds and four hydrophobic interactions with residues located within the CEBPD DNA binding site, as documented in Table 5 and shown in Fig. 7.

**Table 5:** Interactions between myricetin, curcumin, and CEBPD residues

Ligand name	Hydrogen bond (distance Å)	Hydrophobic interaction (distance Å)
Myricetin	Lys213 (4.01); Asn216 (3.36, 4.21, 3.82); Arg215 (2.86, 4.21); Ala212 (2.95)	Arg215 (4.49)
Curcumin	Gln220 (4.82); Asn216 (3.49, 4.21); Arg215 (4.6); Lys213 (3.87)	Lys213 (5.16); Ala212 (5.28); Lys213 (3.87, 5.3)

*CEBPD* CCAAT/enhancer-binding protein beta

## Discussion

Cervical cancer exhibits a substantial prevalence, holding a prominent position among gynecologic cancers on a global scale. Disparities in screening rates and clinical outcomes are notable, with distinct challenges being more pronounced within low socioeconomic and marginalized populations. To alleviate the burden of cervical cancer, primary prevention strategies such as HPV vaccination and targeted interventions play a pivotal role [1]. Moreover, prior research endeavors have revealed the therapeutic efficacy of epicatechin, curcumin, myricetin, jatrorrhizine, and arborinine in cervical cancer. Hence, the current study conducted a comprehensive bioinformatics analysis aimed at delineating the principal genes, pathways, and biological processes underpinning the process of malignant transformation in cervical CxCa. The investigation also elucidates the potential mechanisms responsible for the therapeutic properties of epicatechin, curcumin, myricetin, jatrorrhizine, and arborinine in cervical cancer. Our systems biology analysis outcomes revealed a significant association between the overexpression of key genes, namely MYC, IL6, JUN, RRM2, and VEGFA, and an unfavorable prognosis in individuals diagnosed with CxCa. Additionally, it was observed that CEBPD emerges as a notably influential upstream regulator of these prognostic markers, as evidenced by an NES of 5.972. The structural bioinformatics analysis has provided compelling evidence of myricetin and curcumin displaying a considerable binding affinity to the DNA binding site of CEBPD, meeting the criteria with Δ*G*_binding_ of less than $-$ 7 kcal/mol. The exceptionally strong binding affinity observed between myricetin and CEBPD is particularly noteworthy, with a Δ*G* binding of − 8.44 kcal/mol and a Ki value of 650.77 nM. Myricetin demonstrated seven hydrogen bonds and one hydrophobic interaction within the CEBPD DNA binding site, engaging with amino acids including Ala212, Lys213, Arg215, and Asn216.

Myricetin, a flavonoid abundant in various plants, including berries, herbs, and walnuts, has emerged as a promising bioactive compound renowned for its potent anticancer properties. Numerous research studies have explored the potential anticancer effects of myricetin across a spectrum of malignancies, encompassing colon, breast, prostate, bladder, and pancreatic cancers. Myricetin’s multifaceted attributes encompass anti-inflammatory, anticancer, apoptosis-inducing, and anticarcinogenic properties, effectively curbing cancer cell proliferation [42]. This flavonoid has proven to be a valuable asset in combating cancer through diverse mechanisms. These mechanisms encompass the modulation of inflammatory responses and signal transduction pathways, including angiogenesis regulation, cell cycle arrest, and the induction of apoptosis. Moreover, myricetin has been observed to amplify the chemotherapeutic potential of other anticancer medications [43]. Additionally, it has demonstrated the capacity to enhance apoptotic activity in various cancer types, underscoring its potential as a valuable therapeutic agent [44]. Yi et al. [45] conducted a study to evaluate the anticancer effects of myricetin, methyl eugenol, and cisplatin, individually and in combination, on cervical cancer (HeLa) cells. In their research, HeLa cells were subjected to separate treatments with myricetin and methyl eugenol and co-treatment with cisplatin, with subsequent assessment of cell growth and apoptosis through various assays. The findings from this study demonstrated that the combined administration of myricetin or methyl eugenol with cisplatin exhibited a more pronounced inhibitory effect on cancer cell growth and a heightened capacity to induce apoptosis when compared to treatment with each drug individually. Moreover, the combination therapy yielded a more substantial induction of apoptosis, as evidenced by fluorescence microscopy using Hoechst 33,258 and AO-ETBR staining. Additionally, the co-treatment led to a significant increase in the number of cells in the G0/G1 phase in comparison with single-drug treatments. Furthermore, the combination approach resulted in a greater loss of mitochondrial membrane potential and enhanced Caspase-3 activity when contrasted with individual drug treatments. The authors concluded that the combination of myricetin or methyl eugenol with cisplatin holds substantial potential as a clinical chemotherapeutic strategy for human cervical cancer.

CEBPD, an acronym denoting the CCAAT/enhancer-binding protein delta, emerges as a pivotal transcription factor with significant implications in cancer initiation and advancement. Recent years have borne witness to a burgeoning interest in comprehending the multifaceted roles that CEBPD plays across various cancer types, including but not limited to cervical cancer and pancreatic ductal adenocarcinoma. In a notable study by Zhou et al. [46], a pivotal discovery unfolded as they unveiled a potent avenue to enhance chemosensitivity to cisplatin (CDDP) within cervical cancer cells. This breakthrough hinged upon the inhibition of CEBPD’s nuclear import, a feat achieved by targeting importin 4 (IPO4). The study compellingly demonstrated that CEBPD orchestrates the transcriptional upregulation of DNA-PKcs (PRKDC), a factor intricately linked to CDDP sensitivity. Moreover, the research elucidated how IPO4 magnified the nuclear translocation of CEBPD, thereby activating PRKDC-mediated DNA damage repair. In vitro and in vivo experiments underscored the salient impact of IPO4 and CEBPD knockdown, notably amplifying CDDP-induced cytotoxicity. These findings coalesce to propose an enticing prospect: manipulating the IPO4-CEBPD-PRKDC axis as a viable strategy to heighten chemosensitivity in cervical cancer. In a separate investigation conducted by Quist et al. [47] in 2021, a pioneering exploration into the clinical ramifications of HPV16 E6E7-NFX1-123-regulated genes within cervical cancer development was undertaken. This inquiry unearthed an intriguing correlation between CEBPD and disease progression in cervical precancer and cancer stages. In vitro experiments offered further insights, elucidating the impact of HPV16 E6E7 and exogenous NFX1-123 on the expression of CEBPD. These findings underscore CEBPD’s pivotal involvement in the pathogenesis of cervical cancer, further elucidating its regulation by HPV16 E6E7-NFX1-123.

The current investigation elucidated that CEBPD serves as an upstream regulatory factor for key genes, including MYC, IL6, JUN, RRM2, and VEGFA, all of which have been linked to an unfavorable prognosis in cervical cancer patients. Among these genes, MYC exhibited the most pronounced correlation with an adverse clinical outcome in CxCa patients, as evidenced by an HR of 2 and a *p*-value for HR of 0.0046.

MYC’s involvement in cervical cancer has been the subject of inquiry across multiple studies. Shou et al. [48] conducted a 2018 study focusing on the expression of c-MYC and bcat1 in cervical tissues. Their findings revealed significantly elevated c-MYC expression in cervical cancer tissues compared to normal cervical and cervical intraepithelial neoplasia (CIN). Similarly, the expression of bcat1 was notably higher in cervical cancer tissues when juxtaposed with normal cervical tissues and CIN tissues. Furthermore, a positive correlation emerged between the expressions of c-MYC and bcat1 within cervical squamous carcinoma and adenocarcinoma domains. This led the authors to posit that heightened c-MYC expression may stimulate cervical cancer invasion and metastasis, while escalated bcat1 expression may bolster proliferation, invasion, and metastasis in cervical cancer, potentially exercising a synergistic influence on the pathogenesis of this malignancy. In 2021, Bai et al. [49] delved into the impact of polydatin, an active compound sourced from the roots of *Polygonum cuspidatum*, on cervical cancer. Their research unveiled that polydatin inhibited cell viability, migration, and invasion within cervical cancer cells. This inhibitory influence was closely associated with the suppression of c-MYC expression. Intriguingly, the study also showcased that overexpression of c-MYC counteracted the inhibitory effects of polydatin on cell proliferation and metastasis. As a result, Bai et al. [20] posited that polydatin can suppress cell proliferation and metastasis by inhibiting c-MYC expression in cervical cancer. Furthermore, Hu et al. [50] conducted a study focusing on miR-145’s role in cervical cancer. Their investigation unveiled a negative correlation between miR-145 and genes associated with metabolic reprogramming. MiR-145 was found to impede the proliferation and metastasis of cervical cancer cells by inhibiting aerobic glycolysis. Importantly, the authors demonstrated that miR-145 can directly bind to MYC’s 3′-untranslated region (3′-UTR). Overexpression of MYC was identified as a key regulator of glycolysis-related genes. This study suggested that miR-145 influences aerobic glycolysis through its interaction with MYC, presenting it as a potential therapeutic target for the management of cervical cancer.

Gene set enrichment analysis has unveiled that, in addition to pathways and biological processes intricately linked to the cell cycle and mitotic processes, the “proteoglycan in cancer” pathway (KEGG: 05205) emerges as a pivotal contributor to the malignant transformation of normal cervical epithelia into cancerous tissues. Proteoglycans, an intriguing class of macromolecules characterized by a protein core adorned with covalently attached, lengthy chains of glycosaminoglycans (GAGs) [51], assume a paramount role as major constituents of the extracellular matrix (ECM). Within this context, they play an indispensable role in shaping cell behavior and influencing the properties of the extracellular matrix [51]. In cancer, proteoglycans have demonstrated their involvement in various facets of cancer initiation and progression [52]. Notably, one of their pivotal roles lies in angiogenesis, which generates new blood vessels to support tumor growth [51]. Proteoglycans actively engage with many cytokines and growth factors, thereby promoting angiogenesis and aiding in the recruitment of endothelial cells to facilitate the formation of new blood vessels [51]. Furthermore, proteoglycans influence the signaling pathways that underpin angiogenesis, including the VEGF pathway [52]. Beyond angiogenesis, proteoglycans extend their involvement in tumor cell proliferation, invasion, and metastasis [51]. They interact with cell surface receptors, adhesion molecules, and enzymes housed within the ECM, thereby impacting cell behavior and fostering tumor cell migration and invasion [51]. The capacity of proteoglycans to shape the microenvironment to be permissive for tumor cell invasion and metastasis is a salient aspect of their contribution [51]. Specifically, they modulate the physical attributes of the ECM, such as its stiffness and porosity, facilitating tumor cell migration and invasion [51]. Moreover, proteoglycans have been implicated in the realm of immune evasion by cancer cells [52]. Their influence extends to the modulation of immune cell functionality and the dynamics of interactions between tumor cells and immune cells within the tumor microenvironment [52]. Proteoglycans are known to affect the recruitment and activation of immune cells and the presentation of antigens to the immune system, thereby contributing to immune evasion [52].

## Conclusion

A comprehensive analysis identified 801 DEGs in CxCa tissues compared to healthy tissues, with 516 upregulated and 285 downregulated. A PPIN was meticulously constructed using these DEGs and CxCa-related genes sourced from DisGeNET. Kaplan–Meier survival curves underscored that the overexpression of five central hub genes, MYC, IL6, JUN, RRM2, and VEGFA, was significantly associated with an unfavorable prognosis in CxCa. Notably, it has been postulated that myricetin exhibits substantial binding affinity to CEBPD, a key regulator in the transcription of prognostic markers in CxCa. These results have unveiled both prognostic markers and the underlying molecular mechanisms that drive the malignant transformation in CxCa. Furthermore, it is suggested that targeting transcription factors involved in regulating prognostic markers may represent a promising mechanism through which myricetin exerts its therapeutic potential, potentially leading to curative benefits for patients afflicted with CxCa.

### Supplementary Information

Additional file 1: Fig. S1. The prognostic significance of MYC, IL6, JUN, RRM2, VEGFA, RFC4, EXO1, PCNA, TOP2A, and TYMS was prominently observed in patients diagnosed with CxCa. The graphical representation, with the X-axis denoting the survival time of CxCa patients and the Y-axis representing the corresponding survival probability, was elucidated through Kaplan–Meier survival curves. Furthermore, the dotted lines in the graph delineate the 95% confidence intervals, providing a measure of the statistical reliability associated with the observed survival probabilities. CxCa; cervical cancer.

Additional file 2: Table S1. A total of 801 differentially expressed genes in cervical cancer tissues compared to normal cervical epithelia.

Additional file 3: Table S2. A total of 371 genes found to be associated with cervical cancer according to DisGeNET database.

Additional file 4: Table S3. Pathways enriched in cervical cancer.

Additional file 5: Table S4. Biological processes enriched in cervical cancer.

Additional file 6: Table S5. Molecular functions enriched in cervical cancer.

Additional file 7: Table S6. Cellular components enriched in cervical cancer.

## Data Availability

The datasets used and/or analyzed during the current study are available from the corresponding author upon reasonable request.
