# Supplementary figures and images for: Exploring molecular targets: herbal isolates in cervical cancer therapy
Source: Genomics Inform. 2024 Jun 26;22:9. doi: 10.1186/s44342-024-00008-1 (PMC11201312; doi:10.1186/s44342-024-00008-1)

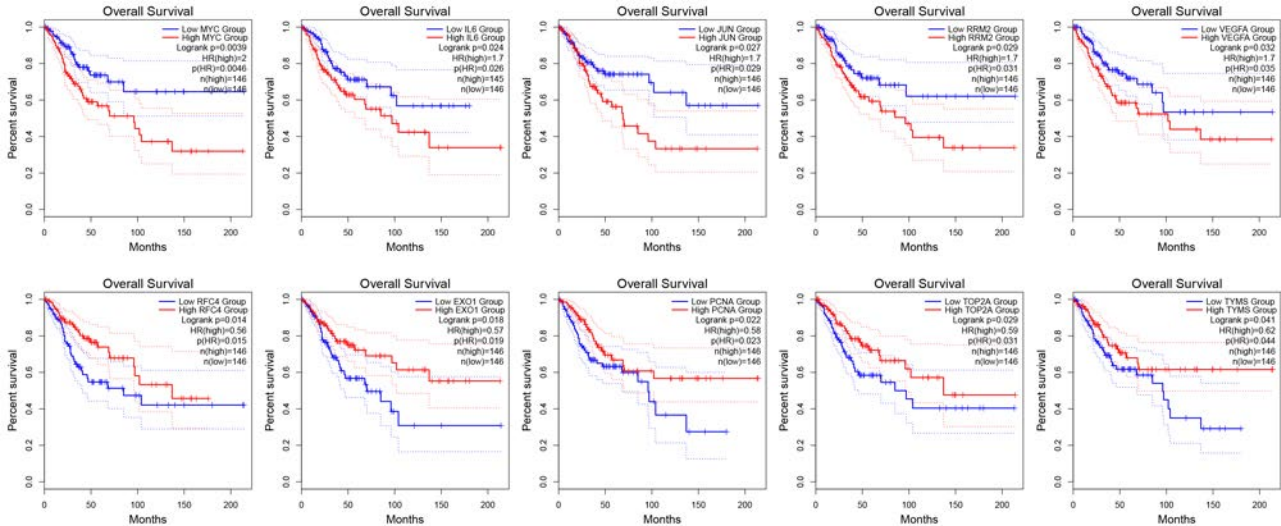

Supplement: Supplementary file 1 — Additional file 1: Fig. S1. The prognostic significance of MYC, IL6, JUN, RRM2, VEGFA, RFC4, EXO1, PCNA, TOP2A, and TYMS was prominently observed in patients diagnosed with CxCa. The graphical representation, with the X-axis denoting the survival time of CxCa patients and the Y-axis representing the corresponding survival probability, was elucidated through Kaplan–Meier survival curves. Furthermore, the dotted lines in the graph delineate the 95% confidence intervals, providing a measure of the statistical reliability associated with the observed survival probabilities. CxCa; cervical cancer. [file 44342_2024_8_MOESM1_ESM.pdf]
