# Supplementary material for: Exploring molecular targets: herbal isolates in cervical cancer therapy
Source: Genomics Inform. 2024 Jun 26;22:9. doi: 10.1186/s44342-024-00008-1 (PMC11201312; doi:10.1186/s44342-024-00008-1)
Supplement: Supplementary file 2 — Additional file 2: Table S1. A total of 801 differentially expressed genes in cervical cancer tissues compared to normal cervical epithelia. [file 44342_2024_8_MOESM2_ESM.pdf]

Gene.symbol	FDR	logFC	|LOG2FC|
CDKN2A	4.96E-07	3.98	3.98
SYCP2	1.6E-06	3.9	3.9
ELAVL2	3.16E-05	3.55	3.55
TCAM1P	1.87E-07	3.55	3.55
HS6ST2	6.53E-06	3.48	3.48
ABCA17P	5.95E-05	3.38	3.38
TMSB15B	3.08E-05	3.33	3.33
SYNGR3	1.13E-05	3.25	3.25
ZFR2	1.38E-05	3.18	3.18
NEFH	0.000469	3.12	3.12
HENMT1	1.59E-06	2.84	2.84
BRIP1	2.57E-08	2.77	2.77
DTL	4.39E-08	2.725	2.725
KLHDC7B	0.000422	2.71	2.71
MYO3A	4.82E-05	2.65	2.65
MIR9-3HG	0.000022	2.64	2.64
MMP12	0.0044	2.6	2.6
TSEN54	9.39E-05	2.59	2.59
E2F7	2.55E-06	2.56	2.56
PRIM1	5.67E-07	2.48	2.48
APOC1	5.45E-05	2.47	2.47
KIAA2022	0.000515	2.43	2.43
CDC25C	3.16E-06	2.38	2.38
CENPE	3.81E-05	2.35	2.35
POLE2	5.13E-07	2.34	2.34
FAM111B	3.72E-05	2.33	2.33
CDC7	3.2E-09	2.32	2.32
DDIAS	2.23E-05	2.32	2.32
CENPK	3.2E-09	2.31	2.31
MLF1	0.000077	2.3	2.3
GINS2	1.23E-07	2.29	2.29
APOBEC3B	6.45E-06	2.28	2.28
HIST1H3F	1.99E-05	2.28	2.28
AIM2	0.00158	2.27	2.27
KIF23	1.27E-07	2.26	2.26
KIF15	2.41E-06	2.22	2.22
CCNE2	1.27E-06	2.21	2.21
MMP9	0.00622	2.21	2.21
CDC6	1.1E-06	2.195	2.195
C3orf70	0.000104	2.16	2.16
MTFR2	2.83E-07	2.16	2.16
SPAG5	5.25E-07	2.16	2.16
WDR66	0.00343	2.16	2.16
NUP210	0.000121	2.14	2.14
BMP8B	1.88E-06	2.14	2.14
WDR76	6.45E-06	2.14	2.14

RMI2	3.96E-09	2.13	2.13
ZNF367	5.24E-08	2.11	2.11
NUSAP1	0.000433	2.105	2.105
UHRF1	1.78E-08	2.1	2.1
C18orf54	0.000144	2.10	2.10
CENPO	6.34E-07	2.09	2.09
TIPIN	1.01E-07	2.09	2.09
WDHD1	1.5E-06	2.08	2.08
HMMR	1.63E-06	2.07	2.07
BBOX1-AS1	0.00362	2.06	2.06
HELLS	0.001215	2.06	2.06
OIP5	0.00006	2.06	2.06
KNL1	2.52E-06	2.05	2.05
FANCA	0.000139	2.04	2.04
TTK	0.000002	2.04	2.04
UGT8	0.00171	2.04	2.04
KIF14	4.15E-05	2.03	2.03
C9orf84	0.000496	2.03	2.03
TICRR	5.76E-06	2.03	2.03
STIL	3.36E-08	2.01	2.01
KIF4A	7.22E-06	2	2
CENPQ	3.87E-06	1.99	1.99
PLK4	0.000187	1.985	1.985
C5orf34	1.29E-06	1.98	1.98
NUF2	2.74E-06	1.98	1.98
CHI3L1	0.000271	1.97	1.97
CA9	0.000365	1.96	1.96
LRR1	1.23E-07	1.96	1.96
SLC35F6	1.04E-05	1.95	1.95
CDKN3	0.00038	1.945	1.945
C1orf112	3.5E-06	1.94	1.94
NEK2	4.33E-07	1.94	1.94
CLEC2B	0.000211	1.93	1.93
RIBC2	1.92E-05	1.93	1.93
DLGAP5	1.05E-06	1.92	1.92
ESCO2	2.55E-06	1.92	1.92
CDT1	6.67E-05	1.905	1.905
CENPI	4.33E-07	1.89	1.89
EAF2	1.92E-05	1.89	1.89
MKI67	4.35E-05	1.89	1.89
SHCBP1	2.49E-07	1.89	1.89
FAM72A	3.5E-06	1.88	1.88
FOXM1	5.68E-05	1.88	1.88
KLHL7	0.000022	1.88	1.88
RBL1	2.32E-07	1.87	1.87
CENPF	3.24E-06	1.86	1.86
ASPM	0.003397	1.84	1.84

CD163	0.001	1.84	1.84
C4orf46	3.71E-05	1.82	1.82
CEP128	0.000316	1.82	1.82
NDC80	6.12E-07	1.82	1.82
ZGRF1	5.67E-07	1.82	1.82
CDK1	4.37E-05	1.82	1.82
CD14	0.00209	1.81	1.81
KIF11	4.12E-06	1.81	1.81
NCR3LG1	0.000699	1.81	1.81
RFC3	1.8E-07	1.8	1.8
CENPN	5.98E-05	1.8	1.8
MCM5	0.00187	1.795	1.795
E2F8	0.000002	1.79	1.79
CABLES2	5.95E-05	1.78	1.78
HPSE	0.00208	1.78	1.78
LIN9	1.29E-06	1.78	1.78
MYO5C	0.000668	1.78	1.78
ZWILCH	7.15E-05	1.775	1.775
CDCA5	9.26E-08	1.77	1.77
CEP55	6.99E-06	1.77	1.77
LINC01133	0.000749	1.77	1.77
MCM2	3.19E-05	1.77	1.77
SPC25	2.74E-06	1.77	1.77
MCM10	0.000398	1.76	1.76
CDCA2	7.94E-05	1.76	1.76
SLC15A3	6.14E-05	1.76	1.76
NETO2	0.000162	1.75	1.75
PKMYT1	4.39E-05	1.75	1.75
FAM26F	0.000529	1.75	1.75
DEPDC1	0.000108	1.74	1.74
MYBL2	1.72E-05	1.74	1.74
RAD51AP1	8.01E-08	1.74	1.74
UBE2T	0.000155	1.74	1.74
SMC2	3.39E-06	1.735	1.735
ADAMDEC1	0.00185	1.73	1.73
KIAA1524	9.96E-06	1.73	1.73
SGO2	0.000323	1.73	1.73
UBL7-AS1	0.000104	1.73	1.73
KIF2C	0.001116	1.72	1.72
GINS1	3.84E-07	1.71	1.71
ARHGAP11B	0.000218	1.7	1.7
MCM6	3.27E-09	1.7	1.7
PARPBP	2.11E-05	1.7	1.7
TCF19	1.27E-05	1.7	1.7
CHEK1	4.31E-05	1.70	1.70
IL17RB	0.00756	1.69	1.69
MDK	0.00258	1.69	1.69

PRR11	3.5E-06	1.69	1.69
MELK	1.87E-07	1.68	1.68
RNASEH2A	7.07E-05	1.68	1.68
AURKA	2.78E-06	1.675	1.675
TOP2A	0.000105	1.67	1.67
KNTC1	3.72E-05	1.67	1.67
MAP4K1	0.0025	1.67	1.67
MTHFD1L	0.00617	1.67	1.67
DNA2	0.000047	1.65	1.65
KIF20A	0.000033	1.65	1.65
RSRC1	0.000404	1.65	1.65
ANLN	5.31E-06	1.64	1.64
BUB1B	4.76E-06	1.64	1.64
TRIM74	0.00362	1.64	1.64
CDC45	1.71E-05	1.63	1.63
RFC4	1.92E-05	1.63	1.63
CSPG5	0.000333	1.62	1.62
MND1	0.000247	1.62	1.62
FBXO5	5.89E-05	1.62	1.62
CCNB1	9.49E-06	1.615	1.615
RRM2	1.45E-05	1.615	1.615
ASB2	0.000159	1.61	1.61
IQGAP3	7.68E-05	1.61	1.61
SLFN13	0.00121	1.61	1.61
FANCI	5.08E-07	1.6	1.6
DEPDC1B	1.71E-05	1.59	1.59
DCLRE1B	7.44E-06	1.59	1.59
FOXF2	0.000753	1.58	1.58
GPSM2	0.00116	1.58	1.58
MX2	0.00578	1.58	1.58
RNF212	0.000067	1.58	1.58
LSG1	0.00525	1.57	1.57
MCM8	0.000366	1.57	1.57
ATAD2	0.000333	1.5625	1.5625
CDC20	0.00265	1.56	1.56
FADS1	8.97E-05	1.56	1.56
NRIP3	0.000963	1.56	1.56
CHP2	0.00984	1.55	1.55
DONSON	3.84E-07	1.55	1.55
EXO1	5.14E-06	1.55	1.55
FAM64A	0.00157	1.55	1.55
GTSE1	0.000666	1.55	1.55
PLEKHG4	1.29E-05	1.55	1.55
RAD54L	2.35E-05	1.55	1.55
INSIG1	6.71E-05	1.55	1.55
NCAPG	1.53E-06	1.545	1.545
CCDC15	0.000155	1.54	1.54

POLQ	0.000508	1.54	1.54
PRC1	0.000002	1.54	1.54
RFPL1S	0.000451	1.54	1.54
TMEM97	0.000971	1.54	1.54
POLA1	6.85E-06	1.53	1.53
TDRKH	9.85E-05	1.53	1.53
TRIP13	2.77E-05	1.53	1.53
FOXD1	0.0021	1.52	1.52
GIPC2	0.000417	1.52	1.52
HERC5	0.000214	1.52	1.52
MESP2	2.52E-05	1.52	1.52
SMC5	0.00105	1.52	1.52
STRIP2	0.000034	1.52	1.52
KLHL35	0.0052	1.51	1.51
KPNA1	0.000242	1.51	1.51
NCK1-AS1	8.94E-05	1.51	1.51
SLAMF8	0.000189	1.51	1.51
WNK3	0.000715	1.51	1.51
CENPU	0.000328	1.5	1.5
MUC4	0.000651	1.5	1.5
STMN1	0.000002	1.5	1.5
TARP	0.00139	1.5	1.5
ZCWPW1	0.0001	1.5	1.5
BUB1	7.49E-05	1.49	1.49
E2F1	0.00013	1.49	1.49
CMPK2	0.00864	1.48	1.48
EPHA4	0.00669	1.48	1.48
INTS7	0.00016	1.48	1.48
LRRCC1	0.000118	1.48	1.48
SLFN11	0.000271	1.48	1.48
ADGRF1	0.00218	1.47	1.47
BIRC5	0.000125	1.47	1.47
HLTF	5.73E-06	1.47	1.47
PDK3	0.000452	1.47	1.47
RMI1	4.71E-07	1.46	1.46
TFR2	0.00135	1.46	1.46
WDR5B	0.000689	1.46	1.46
ZWINT	8.01E-08	1.46	1.46
PXDC1	8.94E-05	1.45	1.45
RFC5	5.65E-07	1.45	1.45
SLC25A28	0.00247	1.45	1.45
SLC7A7	0.00136	1.45	1.45
BLM	1.15E-05	1.44	1.44
CXorf57	0.000967	1.44	1.44
RNASEH2B	2.39E-05	1.44	1.44
ZYG11A	0.000635	1.44	1.44
CDCA7	0.000248	1.43	1.43

CPNE8	0.000736	1.43	1.43
FAM184A	0.00602	1.43	1.43
MCM4	0.000502	1.43	1.43
BCL7A	0.000108	1.42	1.42
BORA	2.41E-05	1.42	1.42
CDC25A	0.000444	1.42	1.42
GABRQ	0.00405	1.42	1.42
NCAPH	1.39E-07	1.42	1.42
RAD51	6.47E-06	1.42	1.42
REEP1	0.00242	1.42	1.42
RAD18	6.1E-05	1.41	1.41
CDCA3	2.77E-05	1.41	1.41
RPP25	0.000215	1.41	1.41
CENPH	0.000175	1.4	1.4
CENPJ	0.000064	1.4	1.4
DLEU2	7.61E-05	1.4	1.4
ECT2	4.06E-06	1.4	1.4
FST	0.00552	1.4	1.4
HAUS8	0.000683	1.4	1.4
RHEBL1	1.42E-05	1.4	1.4
SUV39H2	1.15E-05	1.4	1.4
TK1	0.00083	1.395	1.395
PHF19	0.001914	1.39	1.39
DUT	5.49E-05	1.39	1.39
NAA50	0.000651	1.39	1.39
FAM102B	0.000259	1.38	1.38
KIAA0101	0.000002	1.38	1.38
MICB	0.00325	1.38	1.38
NUP62CL	0.000345	1.38	1.38
PCSK9	0.00278	1.38	1.38
RGS1	0.00722	1.38	1.38
RTKN2	0.00102	1.38	1.38
BARD1	0.001237	1.37	1.37
GBP5	0.00926	1.37	1.37
LAMP3	0.000592	1.37	1.37
CCNA2	8.96E-05	1.365	1.365
C12orf54	0.001093	1.365	1.365
CCNB2	1.59E-05	1.36	1.36
LOC81691	0.000233	1.36	1.36
LRP6	0.000187	1.36	1.36
NEMP1	4.68E-05	1.36	1.36
NFE2L3	0.000163	1.36	1.36
RCC1	0.000383	1.36	1.36
SLC52A1	0.00386	1.36	1.36
TMEM56	0.00394	1.36	1.36
SCCPDH	0.00652	1.355	1.355
C21orf91	0.000281	1.35	1.35

NIN	0.000753	1.35	1.35
STAT1	0.00311	1.35	1.35
EMC3-AS1	0.00611	1.34	1.34
EZH2	6.99E-06	1.34	1.34
FOXRED2	0.000369	1.34	1.34
TEX30	0.000154	1.34	1.34
THRAP3	0.00797	1.34	1.34
GSDMB	0.00192	1.33	1.33
C3orf52	9.54E-05	1.32	1.32
DLEU2L	0.000239	1.32	1.32
HIVEP3	0.00262	1.32	1.32
TAZ	0.00318	1.32	1.32
SSX2IP	0.001428	1.3175	1.3175
HPS3	5.34E-05	1.31	1.31
MTF2	0.00364	1.31	1.31
NEDD1	3.16E-06	1.31	1.31
PLA2G7	0.00281	1.31	1.31
TNPO3	0.000276	1.31	1.31
ZFX	0.000118	1.31	1.31
CCDC138	0.000446	1.3	1.3
CENPW	0.000331	1.3	1.3
ENTPD1	0.00262	1.3	1.3
FA2H	0.00044	1.3	1.3
HOXD11	0.00171	1.3	1.3
SLC25A37	9.21E-05	1.3	1.3
UNC5CL	0.000362	1.3	1.3
TMPO	2.36E-06	1.295	1.295
B3GLCT	0.000417	1.29	1.29
CKS2	1.63E-06	1.29	1.29
LRP8	0.003452	1.285	1.285
COQ4	0.000215	1.28	1.28
NAIP	0.00106	1.28	1.28
PDS5B	0.00415	1.28	1.28
RNASEH2C	0.000691	1.28	1.28
TYMS	3.97E-05	1.28	1.28
ZNF541	0.00457	1.28	1.28
CDCA8	0.000974	1.27	1.27
DSCC1	5.51E-05	1.27	1.27
ERCC6L	0.000141	1.27	1.27
USP1	0.000821	1.265	1.265
C9orf40	1.72E-05	1.26	1.26
CCDC150	0.0029	1.26	1.26
DBF4	4.72E-06	1.26	1.26
DSN1	0.000126	1.26	1.26
GIT2	0.00211	1.26	1.26
HSPBAP1	0.00412	1.26	1.26
NCAPG2	3.26E-05	1.26	1.26

NDE1	1.39E-07	1.26	1.26
TOPBP1	1.01E-07	1.26	1.26
ZNF473	0.00145	1.26	1.26
EPS15L1	0.00339	1.25	1.25
FAM109B	0.00072	1.25	1.25
LINC00467	0.000167	1.25	1.25
LMNB1	0.00145	1.25	1.25
POLE	0.00444	1.25	1.25
RFWD3	0.00137	1.25	1.25
RPL39L	0.000127	1.25	1.25
RTTN	2.23E-05	1.25	1.25
SCYL3	0.00188	1.25	1.25
TNNT1	0.00694	1.25	1.25
FAM49B	0.00191	1.24	1.24
FANCD2	0.000116	1.24	1.24
HP1BP3	0.00205	1.24	1.24
RAD1	0.000607	1.24	1.24
SMC4	0.000381	1.24	1.24
CELSR3	0.00323	1.23	1.23
CSE1L	0.000413	1.23	1.23
NUP107	1.29E-05	1.23	1.23
TCOF1	0.000507	1.23	1.23
TIMELESS	0.000548	1.23	1.23
VEGFA	0.00668	1.23	1.23
DHFR	0.000125	1.225	1.225
C22orf39	0.000591	1.22	1.22
FEN1	7.79E-07	1.22	1.22
FOXA1	0.000911	1.22	1.22
GPR19	5.93E-05	1.22	1.22
PRPS2	0.00435	1.22	1.22
TACC3	0.000261	1.22	1.22
BID	0.000795	1.215	1.215
CAMTA1	0.002495	1.215	1.215
CCSAP	2.83E-07	1.21	1.21
CCT5	0.00704	1.21	1.21
FIGNL1	2.44E-06	1.21	1.21
IRX2	0.00029	1.21	1.21
ORC6	0.000122	1.21	1.21
PIGX	0.000483	1.21	1.21
RYR1	0.00507	1.21	1.21
TXNRD3NB	0.000304	1.21	1.21
VRK1	2.72E-07	1.21	1.21
IPO9	0.000249	1.2	1.2
CROCC	0.00139	1.2	1.2
GK	0.00528	1.2	1.2
KIF18A	0.00166	1.2	1.2
MARCH3	0.00724	1.2	1.2

MCM3	0.000617	1.2	1.2
MCM7	0.00665	1.2	1.2
ZNF597	0.000219	1.2	1.2
ZNF684	0.000156	1.2	1.2
ASF1B	0.00225	1.19	1.19
CKS1B	1.45E-06	1.19	1.19
GPC2	0.000185	1.19	1.19
TMEM38B	0.00492	1.19	1.19
TRIM45	0.00174	1.19	1.19
RRM1	0.003486	1.18	1.18
CKAP2	4.19E-05	1.18	1.18
CNTLN	9.73E-06	1.18	1.18
FLJ45482	0.00326	1.18	1.18
MED30	0.00669	1.18	1.18
RECQL4	0.000796	1.18	1.18
USP18	0.00176	1.18	1.18
ALG10	0.00019	1.17	1.17
CHML	0.000675	1.17	1.17
CHTF18	0.00369	1.17	1.17
EFCAB11	0.00034	1.17	1.17
KIFC1	0.00474	1.17	1.17
SASS6	2.01E-07	1.17	1.17
SPICE1	0.000732	1.17	1.17
MAD2L1	0.000973	1.165	1.165
EML6	0.00154	1.16	1.16
FLJ32255	0.000285	1.16	1.16
KTI12	8.55E-05	1.16	1.16
NCAPD3	2.39E-05	1.16	1.16
PRIM2	8.61E-07	1.16	1.16
SLC36A4	0.000146	1.16	1.16
STAG1	7.95E-05	1.16	1.16
CYP27B1	0.000153	1.15	1.15
DBR1	5.95E-05	1.15	1.15
FNDC3B	0.00315	1.15	1.15
OSGIN2	0.000426	1.15	1.15
SYNM	0.00271	1.15	1.15
MIS18A	0.001236	1.14	1.14
CEP152	0.000431	1.14	1.14
ERVMER34-1	0.00135	1.14	1.14
GABPB1	2.11E-05	1.14	1.14
GGH	0.000345	1.14	1.14
NUDCD1	0.000254	1.14	1.14
NUDT1	0.00844	1.14	1.14
SSR3	0.000622	1.14	1.14
TIMP3	0.00494	1.14	1.14
WHSC1	0.000351	1.14	1.14
ARHGAP4	0.00639	1.13	1.13

ASXL1	0.00246	1.13	1.13
CBX3	0.000107	1.13	1.13
ETV5	0.00108	1.13	1.13
ETV7	0.00463	1.13	1.13
IGSF6	0.002	1.13	1.13
RPGRIP1L	0.000694	1.13	1.13
ANP32E	0.000104	1.12	1.12
PTTG3P	0.000144	1.12	1.12
ANKRD13C	0.000218	1.11	1.11
FAM24B	0.000047	1.11	1.11
LSM5	6.18E-05	1.11	1.11
PBX3	0.0037	1.11	1.11
PTTG1	0.000399	1.11	1.11
RAP2B	0.00312	1.11	1.11
TEAD4	0.00213	1.11	1.11
TMEM116	0.000366	1.11	1.11
CEP78	0.000219	1.11	1.11
AURKB	0.00786	1.1	1.1
CEP126	0.00271	1.1	1.1
DUSP2	0.00533	1.1	1.1
IFI30	0.00113	1.1	1.1
KPNA4	0.00364	1.1	1.1
DERL3	0.00475	1.09	1.09
IFNAR1	0.00793	1.09	1.09
LINC00312	0.00656	1.09	1.09
MORC4	0.00113	1.09	1.09
PAG1	0.00623	1.09	1.09
POC1A	0.000103	1.09	1.09
SENP1	8.63E-05	1.09	1.09
SGO1	0.00022	1.09	1.09
SLC25A19	0.0021	1.09	1.09
DDX20	0.000439	1.08	1.08
FANCM	1.65E-05	1.08	1.08
GDAP2	0.00122	1.08	1.08
MPHOSPH9	0.000652	1.08	1.08
RACGAP1	2.19E-06	1.08	1.08
SLC35G1	0.00428	1.08	1.08
TAF5	0.000301	1.08	1.08
HAUS5	0.001771	1.08	1.08
MCPH1	0.005355	1.08	1.08
CHAF1B	6.58E-05	1.07	1.07
CKLF	3.84E-07	1.07	1.07
MANEAL	0.00779	1.07	1.07
MASTL	0.000215	1.07	1.07
TTLL7	0.005	1.07	1.07
ABHD2	7.81E-05	1.06	1.06
ALG10B	0.000505	1.06	1.06

CLDN1	0.00196	1.06	1.06
HYLS1	0.000151	1.06	1.06
PIK3R3	0.00189	1.06	1.06
RFESD	0.00106	1.06	1.06
SFMBT1	0.000694	1.06	1.06
TMEM106C	0.00994	1.06	1.06
TPGS2	0.000836	1.06	1.06
UGGT2	0.00324	1.06	1.06
AIF1	0.00272	1.05	1.05
CCNE1	0.00481	1.05	1.05
E2F2	0.000629	1.05	1.05
PXYLP1	0.00049	1.05	1.05
TBC1D15	0.000573	1.05	1.05
TOP3A	0.00912	1.05	1.05
TPX2	0.00846	1.05	1.05
APITD1	6.84E-05	1.04	1.04
BRICD5	0.00324	1.04	1.04
GEN1	4.99E-05	1.04	1.04
GMNN	3.34E-06	1.04	1.04
PCNA	8.41E-06	1.04	1.04
SLF1	0.000399	1.04	1.04
TMEM39A	0.000221	1.04	1.04
BAX	0.00107	1.03	1.03
GK3P	0.00113	1.03	1.03
KIF20B	9.61E-05	1.03	1.03
NRM	0.00288	1.03	1.03
PRELID2	0.0045	1.03	1.03
RPA3	7.51E-05	1.03	1.03
TBC1D31	0.00115	1.03	1.03
TMEM206	0.00968	1.03	1.03
UCK2	0.00808	1.03	1.03
ZNF836	0.00165	1.03	1.03
MYNN	0.001146	1.025	1.025
AMN1	0.00122	1.02	1.02
ARHGAP11A	0.000128	1.02	1.02
HUNK	0.000669	1.02	1.02
PRPS1	0.000066	1.02	1.02
SUV39H1	2.41E-05	1.02	1.02
COL8A2	0.000622	1.01	1.01
ERO1B	0.00343	1.01	1.01
FCER1G	0.00824	1.01	1.01
KHDRBS1	0.00426	1.01	1.01
KLHL13	0.00695	1.01	1.01
MYB	0.00856	1.01	1.01
TRIM59	0.00234	1.01	1.01
ZFAT	0.000176	1.01	1.01
GAS2L3	0.000121	0.06	1.66

BAIAP2	0.00572	-1.01	1.01
CLDN17	0.00933	-1.01	1.01
GRAMD2	0.00852	-1.01	1.01
TNRC6B	0.000156	-1.01	1.01
TP53INP2	0.00362	-1.01	1.01
HYMAI	0.00404	-1.02	1.02
MFSD3	0.00618	-1.02	1.02
DOCK9	0.00702	-1.03	1.03
FGFR1	0.00323	-1.03	1.03
MYBBP1A	0.000306	-1.03	1.03
PRPH	0.00503	-1.03	1.03
SLC22A14	0.000064	-1.03	1.03
STXBP6	0.00658	-1.03	1.03
SYNGR1	0.000351	-1.03	1.03
TMPRSS13	0.00618	-1.03	1.03
TPTEP1	0.00269	-1.03	1.03
ZSCAN2	0.000314	-1.03	1.03
CCDC124	0.0025	-1.04	1.04
HIPK2	0.000319	-1.04	1.04
MAP3K10	0.000308	-1.04	1.04
NLRX1	0.00179	-1.04	1.04
PLEKHF1	0.00171	-1.04	1.04
RNPEPL1	0.000104	-1.04	1.04
SHANK2	0.00219	-1.04	1.04
WWC2	0.00233	-1.04	1.04
B3GNT7	0.00161	-1.05	1.05
C4orf3	0.00137	-1.05	1.05
CYP2C9	0.000417	-1.05	1.05
MAGI1	0.000242	-1.05	1.05
PIP	0.000732	-1.05	1.05
PRLR	0.00904	-1.05	1.05
PROM2	0.00844	-1.05	1.05
TCP11L2	0.00164	-1.05	1.05
TMEM159	0.0031	-1.05	1.05
MAFF	0.000163	-1.06	1.06
NRTN	0.00139	-1.06	1.06
HOXB5	0.00287	-1.07	1.07
MYO6	0.00759	-1.07	1.07
TGFA	0.000632	-1.07	1.07
EXOC7	0.000355	-1.08	1.08
FANK1	0.000088	-1.08	1.08
MXRA7	0.000883	-1.08	1.08
PCDHGA1	8.86E-05	-1.08	1.08
PEAR1	0.000936	-1.08	1.08
SLC39A5	0.0017	-1.08	1.08
ACP5	0.00499	-1.09	1.09
C6orf89	0.000621	-1.09	1.09

DLGAP1	5.23E-05	-1.09	1.09
NFATC4	0.00354	-1.09	1.09
RAB40B	0.000922	-1.09	1.09
CFAP70	0.000266	-1.1	1.1
GLG1	9.39E-05	-1.1	1.1
SPINT1	0.00402	-1.1	1.1
UGT2B15	0.00073	-1.1	1.1
CMTM3	0.000578	-1.11	1.11
GSTA4	0.0071	-1.11	1.11
HNMT	0.00175	-1.11	1.11
PLIN3	0.00639	-1.11	1.11
TOLLIP	0.00128	-1.11	1.11
AREG	0.00834	-1.12	1.12
CRTAP	0.000565	-1.12	1.12
ITPR2	0.0013	-1.12	1.12
B3GNT8	0.00512	-1.13	1.13
HLA-G	0.00419	-1.13	1.13
SLC48A1	0.00051	-1.13	1.13
ZBTB43	2.23E-05	-1.13	1.13
EEPD1	0.00273	-1.14	1.14
IGDCC3	0.00761	-1.14	1.14
PDLIM2	0.00113	-1.14	1.14
PPP1R16A	2.12E-05	-1.14	1.14
THAP8	0.00177	-1.14	1.14
ACTR3C	0.000247	-1.15	1.15
EIF2AK2	0.00969	-1.15	1.15
GPR1	0.00022	-1.15	1.15
PLAGL1	0.00333	-1.15	1.15
USP5	0.000402	-1.15	1.15
DACH1	0.000579	-1.16	1.16
EMILIN3	0.000722	-1.16	1.16
HTR4	0.00292	-1.16	1.16
ID4	9.03E-05	-1.16	1.16
OBSCN	0.000811	-1.16	1.16
PER1	7.73E-05	-1.16	1.16
SEPT5	5.95E-05	-1.16	1.16
CERS4	0.0064	-1.17	1.17
COL17A1	0.0052	-1.17	1.17
DIS3L2	8.86E-05	-1.17	1.17
DOK4	0.000179	-1.17	1.17
SYNPO2L	0.00202	-1.17	1.17
LGR6	0.00507	-1.18	1.18
NBR2	0.00645	-1.18	1.18
SLC7A5	0.00935	-1.18	1.18
ZNF264	0.00404	-1.18	1.18
LRRC6	0.00151	-1.19	1.19
ZNF117	0.004313	-1.19	1.19

CFAP69	0.000476	-1.2	1.2
DLL1	0.00163	-1.2	1.2
DYRK3	8.78E-05	-1.2	1.2
TGFBR3	0.00554	-1.2	1.2
SERPINB1	0.000573	-1.21	1.21
CDK16	0.00175	-1.21	1.21
LARS2	0.000161	-1.21	1.21
RARG	0.000342	-1.21	1.21
ACAA1	0.000866	-1.22	1.22
AHNAK	0.00108	-1.22	1.22
HPGD	0.00452	-1.22	1.22
PHACTR4	0.00408	-1.22	1.22
SMPD2	0.000209	-1.22	1.22
ZNF304	0.00211	-1.22	1.22
CABLES1	0.00453	-1.23	1.23
CRYL1	0.000146	-1.23	1.23
FBXL7	0.00192	-1.23	1.23
TRAPPC6A	0.00517	-1.23	1.23
B4GALT1	1.76E-05	-1.24	1.24
NOL3	0.000336	-1.24	1.24
SNX9	0.001686	-1.24	1.24
UBL3	0.000304	-1.24	1.24
PINK1	8.78E-05	-1.25	1.25
PRR15L	0.00924	-1.25	1.25
SH3TC1	0.00154	-1.25	1.25
PHF23	6.44E-05	-1.265	1.265
CFD	0.0036	-1.27	1.27
TTL	0.0021	-1.27	1.27
AKIRIN2	0.0015	-1.28	1.28
FBXO2	0.00501	-1.28	1.28
NUDT18	0.0013	-1.28	1.28
DNM3	0.00488	-1.29	1.29
GNL3L	0.000185	-1.29	1.29
NOVA2	6.45E-05	-1.29	1.29
ZCCHC24	0.000259	-1.29	1.29
TWIST2	0.00575	-1.3	1.3
HSD3B7	0.000241	-1.31	1.31
KLHL3	0.00149	-1.31	1.31
MYL5	0.000127	-1.32	1.32
SZT2	0.000712	-1.32	1.32
ZSCAN18	0.00727	-1.32	1.32
C15orf59	0.002672	-1.33	1.33
C6orf132	0.000621	-1.33	1.33
QSOX1	5.64E-05	-1.33	1.33
SPTLC3	0.0027	-1.33	1.33
UPP1	0.00457	-1.33	1.33
PHLDA1	0.00182	-1.34	1.34

PPP1R3B	0.00466	-1.34	1.34
SDR9C7	0.00288	-1.34	1.34
SLC16A6	0.00091	-1.34	1.34
ZNF667	0.000629	-1.34	1.34
ANKRD35	0.00308	-1.37	1.37
CCNG2	0.000714	-1.37	1.37
LRRC20	0.000327	-1.37	1.37
RAI2	0.00171	-1.37	1.37
SORBS2	0.00765	-1.37	1.37
C1orf186	0.000626	-1.38	1.38
CITED2	0.00281	-1.38	1.38
FAM234B	0.00217	-1.38	1.38
SHROOM3	0.004608	-1.38	1.38
ST8SIA5	0.000127	-1.38	1.38
RHOD	0.000982	-1.385	1.385
CYP2J2	0.00265	-1.39	1.39
SDK2	0.000359	-1.39	1.39
AIF1L	0.000271	-1.4	1.4
BIN1	0.00042	-1.4	1.4
PRSS2	0.0064	-1.4	1.4
SERPINB9P1	0.000717	-1.4	1.4
HFE	0.00325	-1.41	1.41
ZNF134	0.00199	-1.41	1.41
ARHGEF7	0.000101	-1.42	1.42
BORCS6	7.27E-05	-1.42	1.42
ABTB2	0.000239	-1.43	1.43
PAPSS2	0.00711	-1.43	1.43
PLA2G6	0.00197	-1.43	1.43
SLC37A1	0.000169	-1.43	1.43
SLIT2	0.00453	-1.43	1.43
C1QTNF3	0.000573	-1.44	1.44
EVA1B	0.00484	-1.44	1.44
TIMP2	0.000868	-1.45	1.45
RSPH1	0.00193	-1.46	1.46
VAT1	0.000123	-1.46	1.46
ADAMTSL4	0.00149	-1.48	1.48
BNIP3	0.003246	-1.49	1.49
ECHDC2	0.000342	-1.49	1.49
MAPK10	3.39E-05	-1.49	1.49
COL4A6	0.0057	-1.5	1.5
NPAS2	0.000241	-1.5	1.5
FAM20A	0.00556	-1.51	1.51
LDOC1	0.00378	-1.51	1.51
PLAG1	0.000481	-1.51	1.51
ALAD	0.000357	-1.52	1.52
C15orf52	0.00316	-1.52	1.52
TDRP	0.000505	-1.52	1.52

TVP23C	0.0002	-1.53	1.53
GGT2	0.005532	-1.5325	1.5325
C6orf1	3.79E-05	-1.55	1.55
ARRB1	0.000275	-1.555	1.555
BEX4	0.00332	-1.57	1.57
SMOX	4.37E-05	-1.57	1.57
EPHX2	0.00112	-1.58	1.58
IRF2BP1	0.00038	-1.58	1.58
SH3BGRL2	0.00841	-1.58	1.58
MIR31HG	0.002	-1.59	1.59
ZNF415	0.00548	-1.59	1.59
HINT3	0.000521	-1.6	1.6
MAOB	0.000844	-1.6	1.6
ZNF431	0.000156	-1.61	1.61
ACPP	0.00577	-1.62	1.62
CCND1	0.000361	-1.62	1.62
CHMP6	0.0001	-1.62	1.62
F3	0.00025	-1.62	1.62
IL17C	0.000759	-1.62	1.62
PLA2G4F	0.000859	-1.62	1.62
SLC46A2	0.00673	-1.63	1.63
SLC7A2	0.00188	-1.63	1.63
LRRC17	0.00181	-1.64	1.64
NAP1L2	0.000901	-1.64	1.64
CAPS	0.000436	-1.66	1.66
TBX3	0.000304	-1.66	1.66
ZNF365	0.00425	-1.66	1.66
IGFBP6	0.00209	-1.68	1.68
LINC01214	0.00134	-1.68	1.68
ST6GAL2	0.00174	-1.68	1.68
ZNF91	0.00092	-1.68	1.68
TOM1	0.000146	-1.69	1.69
ZNF439	0.000103	-1.69	1.69
SNORA68	0.000543	-1.7	1.7
SLC5A1	0.001839	-1.705	1.705
C2orf54	0.000271	-1.71	1.71
ZNF320	0.00182	-1.71	1.71
ZNF844	0.00158	-1.74	1.74
PTCRA	0.000142	-1.76	1.76
ZNF300P1	0.00789	-1.76	1.76
ZNF506	0.000104	-1.76	1.76
ACOX2	1.99E-05	-1.77	1.77
SPNS2	0.000478	-1.77	1.77
MAB21L3	0.00962	-1.78	1.78
GPAT4	0.00263	-1.79	1.79
THSD4	0.001049	-1.80	1.80
CTTNBP2	3.21E-05	-1.8	1.8

SLC16A9	0.00217	-1.81	1.81
TMEM132C	0.000335	-1.82	1.82
DPP4	0.000192	-1.825	1.825
FLG-AS1	0.00312	-1.83	1.83
MYZAP	0.00533	-1.84	1.84
BICDL2	5.45E-05	-1.85	1.85
APOD	0.00128	-1.86	1.86
SORBS1	0.000837	-1.87	1.87
EEF2KMT	0.000118	-1.88	1.88
GGTLC1	0.000162	-1.88	1.88
SLC15A1	0.00453	-1.88	1.88
HBA2	0.002754	-1.88	1.88
FAM13C	0.000661	-1.89	1.89
ESR1	0.00528	-1.9	1.9
RAB11B-AS1	8.81E-07	-1.9	1.9
RGS20	0.000883	-1.91	1.91
CAPN5	1.63E-05	-1.96	1.96
IL36RN	0.00303	-1.96	1.96
RGS12	0.000492	-1.96	1.96
PDZRN3	0.000883	-1.97	1.97
ATP6V1C2	0.00304	-1.99	1.99
NDN	0.00368	-1.99	1.99
PRSS3P2	0.000574	-2	2
UGT2B17	0.00201	-2.03	2.03
CDA	0.00661	-2.09	2.09
FAM3D	0.00673	-2.1	2.1
DCUN1D3	3.04E-08	-2.15	2.15
TMEM229B	6.17E-05	-2.15	2.15
FBXO32	0.000105	-2.16	2.16
SOSTDC1	0.00979	-2.28	2.28
SLURP1	0.00627	-2.29	2.29
CITED4	0.000193	-2.32	2.32
MMP28	0.000552	-2.32	2.32
EPB41L3	0.000322	-2.35	2.35
RBM20	0.00108	-2.36	2.36
EMP1	0.000211	-2.37	2.37
GYS2	0.00572	-2.37	2.37
GREB1	0.00664	-2.45	2.45
ALOX15B	0.00157	-2.59	2.59
CGNL1	4.91E-05	-2.61	2.61
EREG	0.0011	-2.64	2.64
FMO2	0.00885	-2.65	2.65
PHYHIP	3.49E-06	-2.76	2.76
LEXM	0.000417	-2.8	2.8
ZBED2	0.000643	-3.13	3.13
RPTN	0.00117	-3.27	3.27
UPK1A	0.00396	-3.31	3.31

CRISP2	1.01E-07	-3.81	3.81
CRISP3	5.34E-05	-4.57	4.57
CDKN2C	0.00494	1.11	1.11
