# Supplementary material for: Exploring molecular targets: herbal isolates in cervical cancer therapy
Source: Genomics Inform. 2024 Jun 26;22:9. doi: 10.1186/s44342-024-00008-1 (PMC11201312; doi:10.1186/s44342-024-00008-1)
Supplement: Supplementary file 3 — Additional file 3: Table S2. A total of 371 genes found to be associated with cervical cancer according to DisGeNET database. [file 44342_2024_8_MOESM3_ESM.pdf]

Disease	Disease_id	Gene
Cervical Squamous Cell Carcinoma	C0279671	CDKN2A
Cervical Squamous Cell Carcinoma	C0279671	TP53
Cervical Squamous Cell Carcinoma	C0279671	EGFR
Cervical Squamous Cell Carcinoma	C0279671	VEGFA
Cervical Squamous Cell Carcinoma	C0279671	PIK3CA
Cervical Squamous Cell Carcinoma	C0279671	PIK3CG
Cervical Squamous Cell Carcinoma	C0279671	PIK3CD
Cervical Squamous Cell Carcinoma	C0279671	HLA-DRB1
Cervical Squamous Cell Carcinoma	C0279671	ERBB2
Cervical Squamous Cell Carcinoma	C0279671	PIK3CB
Cervical Squamous Cell Carcinoma	C0279671	H3P10
Cervical Squamous Cell Carcinoma	C0279671	PDXP
Cervical Squamous Cell Carcinoma	C0279671	SMUG1
Cervical Squamous Cell Carcinoma	C0279671	AKT1
Cervical Squamous Cell Carcinoma	C0279671	TP63
Cervical Squamous Cell Carcinoma	C0279671	GSTM1
Cervical Squamous Cell Carcinoma	C0279671	RASSF1
Cervical Squamous Cell Carcinoma	C0279671	CCND1
Cervical Squamous Cell Carcinoma	C0279671	SOX2
Cervical Squamous Cell Carcinoma	C0279671	NME1
Cervical Squamous Cell Carcinoma	C0279671	RBM45
Cervical Squamous Cell Carcinoma	C0279671	CTNNB1
Cervical Squamous Cell Carcinoma	C0279671	MYC
Cervical Squamous Cell Carcinoma	C0279671	UVRAG
Cervical Squamous Cell Carcinoma	C0279671	IL6
Cervical Squamous Cell Carcinoma	C0279671	KRAS
Cervical Squamous Cell Carcinoma	C0279671	CKAP4
Cervical Squamous Cell Carcinoma	C0279671	VIM
Cervical Squamous Cell Carcinoma	C0279671	PDPN
Cervical Squamous Cell Carcinoma	C0279671	TGFB1
Cervical Squamous Cell Carcinoma	C0279671	HIF1A
Cervical Squamous Cell Carcinoma	C0279671	HLA-A
Cervical Squamous Cell Carcinoma	C0279671	HLA-DQB1
Cervical Squamous Cell Carcinoma	C0279671	OSMR
Cervical Squamous Cell Carcinoma	C0279671	RPE65
Cervical Squamous Cell Carcinoma	C0279671	ST6GAL1
Cervical Squamous Cell Carcinoma	C0279671	BSG
Cervical Squamous Cell Carcinoma	C0279671	AURKA
Cervical Squamous Cell Carcinoma	C0279671	FHIT
Cervical Squamous Cell Carcinoma	C0279671	KIT
Cervical Squamous Cell Carcinoma	C0279671	TWIST1
Cervical Squamous Cell Carcinoma	C0279671	NQO1
Cervical Squamous Cell Carcinoma	C0279671	PTEN
Cervical Squamous Cell Carcinoma	C0279671	RTN4
Cervical Squamous Cell Carcinoma	C0279671	MIR143
Cervical Squamous Cell Carcinoma	C0279671	TLR4

Cervical Squamous Cell Carcinoma	C0279671	TLR1
Cervical Squamous Cell Carcinoma	C0279671	TP73
Cervical Squamous Cell Carcinoma	C0279671	ITGB1
Cervical Squamous Cell Carcinoma	C0279671	C1QBP
Cervical Squamous Cell Carcinoma	C0279671	ESR1
Cervical Squamous Cell Carcinoma	C0279671	ACKR3
Cervical Squamous Cell Carcinoma	C0279671	GABPA
Cervical Squamous Cell Carcinoma	C0279671	GSTT1
Cervical Squamous Cell Carcinoma	C0279671	ARHGAP24
Cervical Squamous Cell Carcinoma	C0279671	CISH
Cervical Squamous Cell Carcinoma	C0279671	SLC52A3
Cervical Squamous Cell Carcinoma	C0279671	NFE2L2
Cervical Squamous Cell Carcinoma	C0279671	HGF
Cervical Squamous Cell Carcinoma	C0279671	ST3GAL4
Cervical Squamous Cell Carcinoma	C0279671	MDM2
Cervical Squamous Cell Carcinoma	C0279671	TSHZ1
Cervical Squamous Cell Carcinoma	C0279671	MMP9
Cervical Squamous Cell Carcinoma	C0279671	MIR221
Cervical Squamous Cell Carcinoma	C0279671	CXCL14
Cervical Squamous Cell Carcinoma	C0279671	KRT19
Cervical Squamous Cell Carcinoma	C0279671	LGALS1
Cervical Squamous Cell Carcinoma	C0279671	KEAP1
Cervical Squamous Cell Carcinoma	C0279671	MAPK1
Cervical Squamous Cell Carcinoma	C0279671	MCM5
Cervical Squamous Cell Carcinoma	C0279671	PPP1R2C
Cervical Squamous Cell Carcinoma	C0279671	ERCC2
Cervical Squamous Cell Carcinoma	C0279671	MIR21
Cervical Squamous Cell Carcinoma	C0279671	NCOA5
Cervical Squamous Cell Carcinoma	C0279671	PTK6
Cervical Squamous Cell Carcinoma	C0279671	S100B
Cervical Squamous Cell Carcinoma	C0279671	MIB1
Cervical Squamous Cell Carcinoma	C0279671	TLR2
Cervical Squamous Cell Carcinoma	C0279671	RBL1
Cervical Squamous Cell Carcinoma	C0279671	PVT1
Cervical Squamous Cell Carcinoma	C0279671	BCL2
Cervical Squamous Cell Carcinoma	C0279671	RARB
Cervical Squamous Cell Carcinoma	C0279671	RAD51
Cervical Squamous Cell Carcinoma	C0279671	BCL2L2
Cervical Squamous Cell Carcinoma	C0279671	ACTB
Cervical Squamous Cell Carcinoma	C0279671	ROCK1
Cervical Squamous Cell Carcinoma	C0279671	BDNF
Cervical Squamous Cell Carcinoma	C0279671	S100A9
Cervical Squamous Cell Carcinoma	C0279671	RAB2A
Cervical Squamous Cell Carcinoma	C0279671	S100A11
Cervical Squamous Cell Carcinoma	C0279671	CXCL12
Cervical Squamous Cell Carcinoma	C0279671	MIR499A
Cervical Squamous Cell Carcinoma	C0279671	POU5F1

Cervical Squamous Cell Carcinoma	C0279671	TLR9
Cervical Squamous Cell Carcinoma	C0279671	RIPK4
Cervical Squamous Cell Carcinoma	C0279671	DUOX1
Cervical Squamous Cell Carcinoma	C0279671	PGR
Cervical Squamous Cell Carcinoma	C0279671	ERAP1
Cervical Squamous Cell Carcinoma	C0279671	NLK
Cervical Squamous Cell Carcinoma	C0279671	RBMX2
Cervical Squamous Cell Carcinoma	C0279671	REV1
Cervical Squamous Cell Carcinoma	C0279671	ATRAID
Cervical Squamous Cell Carcinoma	C0279671	WT1-AS
Cervical Squamous Cell Carcinoma	C0279671	TLR7
Cervical Squamous Cell Carcinoma	C0279671	PCNA
Cervical Squamous Cell Carcinoma	C0279671	TMED7
Cervical Squamous Cell Carcinoma	C0279671	P2RX7
Cervical Squamous Cell Carcinoma	C0279671	UGT1A1
Cervical Squamous Cell Carcinoma	C0279671	TRIT1
Cervical Squamous Cell Carcinoma	C0279671	GOLPH3L
Cervical Squamous Cell Carcinoma	C0279671	PTHLH
Cervical Squamous Cell Carcinoma	C0279671	MIR362
Cervical Squamous Cell Carcinoma	C0279671	DANCR
Cervical Squamous Cell Carcinoma	C0279671	PTCH1
Cervical Squamous Cell Carcinoma	C0279671	PSMD9
Cervical Squamous Cell Carcinoma	C0279671	KLF13
Cervical Squamous Cell Carcinoma	C0279671	CHPT1
Cervical Squamous Cell Carcinoma	C0279671	PSMB9
Cervical Squamous Cell Carcinoma	C0279671	PSMB8
Cervical Squamous Cell Carcinoma	C0279671	TCIM
Cervical Squamous Cell Carcinoma	C0279671	DIABLO
Cervical Squamous Cell Carcinoma	C0279671	MAPK3
Cervical Squamous Cell Carcinoma	C0279671	PRKCD
Cervical Squamous Cell Carcinoma	C0279671	EXOC1
Cervical Squamous Cell Carcinoma	C0279671	AGK
Cervical Squamous Cell Carcinoma	C0279671	OPRD1
Cervical Squamous Cell Carcinoma	C0279671	CDH1
Cervical Squamous Cell Carcinoma	C0279671	NCOA3
Cervical Squamous Cell Carcinoma	C0279671	CANX
Cervical Squamous Cell Carcinoma	C0279671	CASP3
Cervical Squamous Cell Carcinoma	C0279671	CASP8
Cervical Squamous Cell Carcinoma	C0279671	DPY30
Cervical Squamous Cell Carcinoma	C0279671	IFITM1
Cervical Squamous Cell Carcinoma	C0279671	DCLK3
Cervical Squamous Cell Carcinoma	C0279671	BECN1
Cervical Squamous Cell Carcinoma	C0279671	MIA
Cervical Squamous Cell Carcinoma	C0279671	CALR
Cervical Squamous Cell Carcinoma	C0279671	FTO
Cervical Squamous Cell Carcinoma	C0279671	AIMP2
Cervical Squamous Cell Carcinoma	C0279671	HAND2-AS1

Cervical Squamous Cell Carcinoma	C0279671	NANOG
Cervical Squamous Cell Carcinoma	C0279671	DHDDS
Cervical Squamous Cell Carcinoma	C0279671	PSCA
Cervical Squamous Cell Carcinoma	C0279671	ITPKC
Cervical Squamous Cell Carcinoma	C0279671	KCNH6
Cervical Squamous Cell Carcinoma	C0279671	SRSF9
Cervical Squamous Cell Carcinoma	C0279671	SERPINH1
Cervical Squamous Cell Carcinoma	C0279671	CD163
Cervical Squamous Cell Carcinoma	C0279671	COX5A
Cervical Squamous Cell Carcinoma	C0279671	CD28
Cervical Squamous Cell Carcinoma	C0279671	GRAP2
Cervical Squamous Cell Carcinoma	C0279671	CD44
Cervical Squamous Cell Carcinoma	C0279671	RASSF2
Cervical Squamous Cell Carcinoma	C0279671	CDC6
Cervical Squamous Cell Carcinoma	C0279671	MFN2
Cervical Squamous Cell Carcinoma	C0279671	KLF4
Cervical Squamous Cell Carcinoma	C0279671	PIWIL1
Cervical Squamous Cell Carcinoma	C0279671	TNFRSF11A
Cervical Squamous Cell Carcinoma	C0279671	IL18R1
Cervical Squamous Cell Carcinoma	C0279671	CFLAR
Cervical Squamous Cell Carcinoma	C0279671	RPS6KA4
Cervical Squamous Cell Carcinoma	C0279671	CLDN1
Cervical Squamous Cell Carcinoma	C0279671	USP8
Cervical Squamous Cell Carcinoma	C0279671	CD247
Cervical Squamous Cell Carcinoma	C0279671	MTDH
Cervical Squamous Cell Carcinoma	C0279671	SLC25A20
Cervical Squamous Cell Carcinoma	C0279671	MALL
Cervical Squamous Cell Carcinoma	C0279671	SP1
Cervical Squamous Cell Carcinoma	C0279671	SPP1
Cervical Squamous Cell Carcinoma	C0279671	SSRP1
Cervical Squamous Cell Carcinoma	C0279671	STAT1
Cervical Squamous Cell Carcinoma	C0279671	STAT3
Cervical Squamous Cell Carcinoma	C0279671	KLF5
Cervical Squamous Cell Carcinoma	C0279671	TAP1
Cervical Squamous Cell Carcinoma	C0279671	TAZ
Cervical Squamous Cell Carcinoma	C0279671	SOS2
Cervical Squamous Cell Carcinoma	C0279671	MIR542
Cervical Squamous Cell Carcinoma	C0279671	DEPTOR
Cervical Squamous Cell Carcinoma	C0279671	ST3GAL1
Cervical Squamous Cell Carcinoma	C0279671	ST3GAL3
Cervical Squamous Cell Carcinoma	C0279671	SIM2
Cervical Squamous Cell Carcinoma	C0279671	SLC1A5
Cervical Squamous Cell Carcinoma	C0279671	SLC2A1
Cervical Squamous Cell Carcinoma	C0279671	HLTF
Cervical Squamous Cell Carcinoma	C0279671	SNAI1
Cervical Squamous Cell Carcinoma	C0279671	TBX2
Cervical Squamous Cell Carcinoma	C0279671	TCF3

Cervical Squamous Cell Carcinoma	C0279671	TPM3
Cervical Squamous Cell Carcinoma	C0279671	TYMS
Cervical Squamous Cell Carcinoma	C0279671	VPS51
Cervical Squamous Cell Carcinoma	C0279671	YY1
Cervical Squamous Cell Carcinoma	C0279671	ZIC1
Cervical Squamous Cell Carcinoma	C0279671	CA2
Cervical Squamous Cell Carcinoma	C0279671	CA9
Cervical Squamous Cell Carcinoma	C0279671	PAX8
Cervical Squamous Cell Carcinoma	C0279671	TNF
Cervical Squamous Cell Carcinoma	C0279671	TM7SF2
Cervical Squamous Cell Carcinoma	C0279671	MIR642A
Cervical Squamous Cell Carcinoma	C0279671	ZEB1
Cervical Squamous Cell Carcinoma	C0279671	TMBIM6
Cervical Squamous Cell Carcinoma	C0279671	TERT
Cervical Squamous Cell Carcinoma	C0279671	THBS1
Cervical Squamous Cell Carcinoma	C0279671	THBS2
Cervical Squamous Cell Carcinoma	C0279671	TIMP3
Cervical Squamous Cell Carcinoma	C0279671	TLR3
Cervical Squamous Cell Carcinoma	C0279671	SELE
Cervical Squamous Cell Carcinoma	C0279671	CPOX
Cervical Squamous Cell Carcinoma	C0279671	DDC
Cervical Squamous Cell Carcinoma	C0279671	DDX53
Cervical Squamous Cell Carcinoma	C0279671	AFP
Cervical Squamous Cell Carcinoma	C0279671	AGA
Cervical Squamous Cell Carcinoma	C0279671	DMBT1
Cervical Squamous Cell Carcinoma	C0279671	DNM2
Cervical Squamous Cell Carcinoma	C0279671	DNMT1
Cervical Squamous Cell Carcinoma	C0279671	DUT
Cervical Squamous Cell Carcinoma	C0279671	DAPK1
Cervical Squamous Cell Carcinoma	C0279671	CYBB
Cervical Squamous Cell Carcinoma	C0279671	CRK
Cervical Squamous Cell Carcinoma	C0279671	MAPK14
Cervical Squamous Cell Carcinoma	C0279671	CSF2RB
Cervical Squamous Cell Carcinoma	C0279671	OR2AG1
Cervical Squamous Cell Carcinoma	C0279671	CTLA4
Cervical Squamous Cell Carcinoma	C0279671	DNAJB7
Cervical Squamous Cell Carcinoma	C0279671	CTSD
Cervical Squamous Cell Carcinoma	C0279671	ZNF385B
Cervical Squamous Cell Carcinoma	C0279671	EPHX2
Cervical Squamous Cell Carcinoma	C0279671	EPO
Cervical Squamous Cell Carcinoma	C0279671	EPOR
Cervical Squamous Cell Carcinoma	C0279671	FGF7
Cervical Squamous Cell Carcinoma	C0279671	FGFR2
Cervical Squamous Cell Carcinoma	C0279671	VASH1
Cervical Squamous Cell Carcinoma	C0279671	FOXM1
Cervical Squamous Cell Carcinoma	C0279671	EPB41L3
Cervical Squamous Cell Carcinoma	C0279671	FLT1

Cervical Squamous Cell Carcinoma	C0279671	SASH1
Cervical Squamous Cell Carcinoma	C0279671	SYNE1
Cervical Squamous Cell Carcinoma	C0279671	FBN1
Cervical Squamous Cell Carcinoma	C0279671	FABP4
Cervical Squamous Cell Carcinoma	C0279671	ERBB3
Cervical Squamous Cell Carcinoma	C0279671	ERBB4
Cervical Squamous Cell Carcinoma	C0279671	ERCC1
Cervical Squamous Cell Carcinoma	C0279671	ERCC4
Cervical Squamous Cell Carcinoma	C0279671	AKT2
Cervical Squamous Cell Carcinoma	C0279671	EZH2
Cervical Squamous Cell Carcinoma	C0279671	F3
Cervical Squamous Cell Carcinoma	C0279671	ALDH1A1
Cervical Squamous Cell Carcinoma	C0279671	SIRT3
Cervical Squamous Cell Carcinoma	C0279671	ADA
Cervical Squamous Cell Carcinoma	C0279671	SLC44A3-AS1
Cervical Squamous Cell Carcinoma	C0279671	LNCNEF
Cervical Squamous Cell Carcinoma	C0279671	CDK6
Cervical Squamous Cell Carcinoma	C0279671	MSLN
Cervical Squamous Cell Carcinoma	C0279671	CDKN1B
Cervical Squamous Cell Carcinoma	C0279671	NET1
Cervical Squamous Cell Carcinoma	C0279671	ADAR
Cervical Squamous Cell Carcinoma	C0279671	CDKN3
Cervical Squamous Cell Carcinoma	C0279671	LINC01507
Cervical Squamous Cell Carcinoma	C0279671	CCAT2
Cervical Squamous Cell Carcinoma	C0279671	SRA1
Cervical Squamous Cell Carcinoma	C0279671	PDCD6
Cervical Squamous Cell Carcinoma	C0279671	TMED7-TICAM2
Cervical Squamous Cell Carcinoma	C0279671	MIR378C
Cervical Squamous Cell Carcinoma	C0279671	MAGI2-AS3
Cervical Squamous Cell Carcinoma	C0279671	BCL2L2-PABPN1
Cervical Squamous Cell Carcinoma	C0279671	KIF20A
Cervical Squamous Cell Carcinoma	C0279671	EBI3
Cervical Squamous Cell Carcinoma	C0279671	CEBPA
Cervical Squamous Cell Carcinoma	C0279671	CIB1
Cervical Squamous Cell Carcinoma	C0279671	ZNRD2
Cervical Squamous Cell Carcinoma	C0279671	CTHRC1
Cervical Squamous Cell Carcinoma	C0279671	RMI2
Cervical Squamous Cell Carcinoma	C0279671	ANIB1
Cervical Squamous Cell Carcinoma	C0279671	CLCN3
Cervical Squamous Cell Carcinoma	C0279671	CHCHD1
Cervical Squamous Cell Carcinoma	C0279671	NAT2
Cervical Squamous Cell Carcinoma	C0279671	ADM
Cervical Squamous Cell Carcinoma	C0279671	COX8A
Cervical Squamous Cell Carcinoma	C0279671	H3P23
Cervical Squamous Cell Carcinoma	C0279671	CKB
Cervical Squamous Cell Carcinoma	C0279671	AHSA1
Cervical Squamous Cell Carcinoma	C0279671	CDC42EP3

Cervical Squamous Cell Carcinoma	C0279671	CTCF
Cervical Squamous Cell Carcinoma	C0279671	CD226
Cervical Squamous Cell Carcinoma	C0279671	DCTN6
Cervical Squamous Cell Carcinoma	C0279671	ZNF268
Cervical Squamous Cell Carcinoma	C0279671	HPSE
Cervical Squamous Cell Carcinoma	C0279671	WIF1
Cervical Squamous Cell Carcinoma	C0279671	CD109
Cervical Squamous Cell Carcinoma	C0279671	FOLH1
Cervical Squamous Cell Carcinoma	C0279671	IL12B
Cervical Squamous Cell Carcinoma	C0279671	KRT14
Cervical Squamous Cell Carcinoma	C0279671	KRT17
Cervical Squamous Cell Carcinoma	C0279671	MIRLET7I
Cervical Squamous Cell Carcinoma	C0279671	MIR122
Cervical Squamous Cell Carcinoma	C0279671	MIR181A2
Cervical Squamous Cell Carcinoma	C0279671	MIR205
Cervical Squamous Cell Carcinoma	C0279671	MIR206
Cervical Squamous Cell Carcinoma	C0279671	MIR29A
Cervical Squamous Cell Carcinoma	C0279671	KRT13
Cervical Squamous Cell Carcinoma	C0279671	KRT8
Cervical Squamous Cell Carcinoma	C0279671	IL12RB2
Cervical Squamous Cell Carcinoma	C0279671	IL17A
Cervical Squamous Cell Carcinoma	C0279671	IL18
Cervical Squamous Cell Carcinoma	C0279671	INHA
Cervical Squamous Cell Carcinoma	C0279671	ITPR3
Cervical Squamous Cell Carcinoma	C0279671	JUN
Cervical Squamous Cell Carcinoma	C0279671	KRT5
Cervical Squamous Cell Carcinoma	C0279671	KRT7
Cervical Squamous Cell Carcinoma	C0279671	MIR30D
Cervical Squamous Cell Carcinoma	C0279671	MIR31
Cervical Squamous Cell Carcinoma	C0279671	MIR99A
Cervical Squamous Cell Carcinoma	C0279671	MSN
Cervical Squamous Cell Carcinoma	C0279671	MUTYH
Cervical Squamous Cell Carcinoma	C0279671	NCF2
Cervical Squamous Cell Carcinoma	C0279671	NCK1
Cervical Squamous Cell Carcinoma	C0279671	NFKB1
Cervical Squamous Cell Carcinoma	C0279671	NNMT
Cervical Squamous Cell Carcinoma	C0279671	MIR375
Cervical Squamous Cell Carcinoma	C0279671	OGG1
Cervical Squamous Cell Carcinoma	C0279671	MIR373
Cervical Squamous Cell Carcinoma	C0279671	MPO
Cervical Squamous Cell Carcinoma	C0279671	EPCAM
Cervical Squamous Cell Carcinoma	C0279671	SMAD4
Cervical Squamous Cell Carcinoma	C0279671	MAL
Cervical Squamous Cell Carcinoma	C0279671	IFNG
Cervical Squamous Cell Carcinoma	C0279671	MET
Cervical Squamous Cell Carcinoma	C0279671	MKI67
Cervical Squamous Cell Carcinoma	C0279671	MLH1

Cervical Squamous Cell Carcinoma	C0279671	MMP2
Cervical Squamous Cell Carcinoma	C0279671	OGN
Cervical Squamous Cell Carcinoma	C0279671	FOLR1
Cervical Squamous Cell Carcinoma	C0279671	CXCL1
Cervical Squamous Cell Carcinoma	C0279671	PDIA3
Cervical Squamous Cell Carcinoma	C0279671	ICOS
Cervical Squamous Cell Carcinoma	C0279671	DNMT3L
Cervical Squamous Cell Carcinoma	C0279671	ANXA5
Cervical Squamous Cell Carcinoma	C0279671	ANXA6
Cervical Squamous Cell Carcinoma	C0279671	HLA-B
Cervical Squamous Cell Carcinoma	C0279671	HLA-DPB1
Cervical Squamous Cell Carcinoma	C0279671	UHRF1
Cervical Squamous Cell Carcinoma	C0279671	CD274
Cervical Squamous Cell Carcinoma	C0279671	TINCR
Cervical Squamous Cell Carcinoma	C0279671	RNF19A
Cervical Squamous Cell Carcinoma	C0279671	POLDIP2
Cervical Squamous Cell Carcinoma	C0279671	GATA6
Cervical Squamous Cell Carcinoma	C0279671	AATF
Cervical Squamous Cell Carcinoma	C0279671	ANGPT1
Cervical Squamous Cell Carcinoma	C0279671	ANGPT2
Cervical Squamous Cell Carcinoma	C0279671	GRB10
Cervical Squamous Cell Carcinoma	C0279671	HLA-DQA1
Cervical Squamous Cell Carcinoma	C0279671	HLA-G
Cervical Squamous Cell Carcinoma	C0279671	HMGB1
Cervical Squamous Cell Carcinoma	C0279671	LINC01193
Cervical Squamous Cell Carcinoma	C0279671	IGFBP6
Cervical Squamous Cell Carcinoma	C0279671	TICAM2
Cervical Squamous Cell Carcinoma	C0279671	FAS
Cervical Squamous Cell Carcinoma	C0279671	IL1A
Cervical Squamous Cell Carcinoma	C0279671	IL4R
Cervical Squamous Cell Carcinoma	C0279671	CXCL8
Cervical Squamous Cell Carcinoma	C0279671	IL10
Cervical Squamous Cell Carcinoma	C0279671	APOE
Cervical Squamous Cell Carcinoma	C0279671	IGF1
Cervical Squamous Cell Carcinoma	C0279671	HMGB2
Cervical Squamous Cell Carcinoma	C0279671	APC
Cervical Squamous Cell Carcinoma	C0279671	HRAS
Cervical Squamous Cell Carcinoma	C0279671	BIRC2
Cervical Squamous Cell Carcinoma	C0279671	HSPA4
Cervical Squamous Cell Carcinoma	C0279671	HSPA8
Cervical Squamous Cell Carcinoma	C0279671	IFI27
Cervical Squamous Cell Carcinoma	C0279671	IFNA17
Cervical Squamous Cell Carcinoma	C0279671	IL10RA
