# Supplementary material for: Exploring molecular targets: herbal isolates in cervical cancer therapy
Source: Genomics Inform. 2024 Jun 26;22:9. doi: 10.1186/s44342-024-00008-1 (PMC11201312; doi:10.1186/s44342-024-00008-1)
Supplement: Supplementary file 4 — Additional file 4: Table S3. Pathways enriched in cervical cancer. [file 44342_2024_8_MOESM4_ESM.pdf]

Cluster no.	source	Pathway name	Pathway ID	FDR
	REAC	Cell Cycle	REAC:R-HSA-1640170	1.76E-42
	REAC	Cell Cycle, Mitotic	REAC:R-HSA-69278	2.24E-41
	REAC	Cell Cycle Checkpoints	REAC:R-HSA-69620	3.14E-28
	KEGG	Cell cycle	KEGG:04110	4.86E-25
	REAC	Mitotic Prometaphase	REAC:R-HSA-68877	1.68E-24
	REAC	Resolution of Sister Chromatid Cohesi	REAC:R-HSA-2500257	1.23E-22
	REAC	M Phase	REAC:R-HSA-68886	5.10E-21
	REAC	Mitotic Anaphase	REAC:R-HSA-68882	1.53E-18
	REAC	Mitotic Metaphase and Anaphase	REAC:R-HSA-2555396	1.68E-18
	REAC	Amplification of signal from unattach	REAC:R-HSA-141444	4.20E-18
	REAC	Amplification of signal from the kineto	REAC:R-HSA-141424	4.20E-18
	REAC	Mitotic Spindle Checkpoint	REAC:R-HSA-69618	7.47E-17
	REAC	EML4 and NUDC in mitotic spindle forr	REAC:R-HSA-9648025	1.84E-16
1	REAC	Separation of Sister Chromatids	REAC:R-HSA-2467813	8.25E-16
	REAC	RHO GTPases Activate Formins	REAC:R-HSA-5663220	2.96E-15
	REAC	RHO GTPase Effectors	REAC:R-HSA-195258	6.01E-13
	REAC	Signaling by Rho GTPases	REAC:R-HSA-194315	1.19E-09
	REAC	Signaling by Rho GTPases, Miro GTPas	REAC:R-HSA-9716542	1.81E-09
	REAC	Regulation of mitotic cell cycle	REAC:R-HSA-453276	1.06E-08
	REAC	APC/C-mediated degradation of cell cy	REAC:R-HSA-174143	1.06E-08
	REAC	G2/M Checkpoints	REAC:R-HSA-69481	2.19E-08
	REAC	Mitotic G1 phase and G1/S transition	REAC:R-HSA-453279	9.75E-08
	REAC	G2/M Transition	REAC:R-HSA-69275	1.42E-07
	REAC	Mitotic G2-G2/M phases	REAC:R-HSA-453274	1.60E-07
	REAC	G1/S Transition	REAC:R-HSA-69206	5.25E-07
	REAC	Transcriptional Regulation by TP53	REAC:R-HSA-3700989	0.00012354
	KEGG	Proteoglycans in cancer	KEGG:05205	9.14E-19
	KEGG	Endocrine resistance	KEGG:01522	9.45E-15
	KEGG	Pathways in cancer	KEGG:05200	1.49E-14
	KEGG	Endometrial cancer	KEGG:05213	1.86E-13
	KEGG	Colorectal cancer	KEGG:05210	1.93E-13
	KEGG	Prostate cancer	KEGG:05215	4.12E-11
	KEGG	Kaposi sarcoma-associated herpesviru	KEGG:05167	5.98E-11
	KEGG	Breast cancer	KEGG:05224	8.24E-11
	KEGG	Gastric cancer	KEGG:05226	8.88E-11
	KEGG	Human cytomegalovirus infection	KEGG:05163	3.14E-10
	KEGG	Thyroid hormone signaling pathway	KEGG:04919	3.94E-10
	KEGG	Focal adhesion	KEGG:04510	2.70E-09
	KEGG	Rap1 signaling pathway	KEGG:04015	4.11E-09
	KEGG	Cellular senescence	KEGG:04218	5.06E-09
	KEGG	Hepatitis B	KEGG:05161	6.93E-09
	KEGG	Hepatocellular carcinoma	KEGG:05225	9.39E-09
	KEGG	MicroRNAs in cancer	KEGG:05206	1.50E-08
2	REAC	Signaling by Receptor Tyrosine Kinases	REAC:R-HSA-9006934	2.38E-08
	KEGG	Chemical carcinogenesis - receptor act	KEGG:05207	9.95E-08
	REAC	ESR-mediated signaling	REAC:R-HSA-8939211	1.80E-07

	KEGG	Salmonella infection	KEGG:05132	4.97E-07
	KEGG	MAPK signaling pathway	KEGG:04010	2.4394E-06
	REAC	Signaling by Nuclear Receptors	REAC:R-HSA-9006931	3.0928E-06
	KEGG	Human papillomavirus infection	KEGG:05165	7.4919E-06
	REAC	DNA Repair	REAC:R-HSA-73894	1.0262E-05
	KEGG	PI3K-Akt signaling pathway	KEGG:04151	1.3705E-05
	REAC	Cytokine Signaling in Immune system	REAC:R-HSA-1280215	2.4827E-05
	REAC	Signaling by Interleukins	REAC:R-HSA-449147	2.6109E-05
	REAC	Generic Transcription Pathway	REAC:R-HSA-212436	4.5313E-05
	REAC	Gene expression (Transcription)	REAC:R-HSA-74160	9.5895E-05
	REAC	RNA Polymerase II Transcription	REAC:R-HSA-73857	0.00016681
	REAC	Disease	REAC:R-HSA-1643685	0.00018603
	REAC	Signal Transduction	REAC:R-HSA-162582	0.00034846
	REAC	Cell Cycle	REAC:R-HSA-1640170	0.00101021
	REAC	Immune System	REAC:R-HSA-168256	0.03882917
3	REAC	Cell Cycle	REAC:R-HSA-1640170	4.32E-15
	REAC	Cell Cycle, Mitotic	REAC:R-HSA-69278	7.68E-15
4	KEGG	Proteoglycans in cancer	KEGG:05205	7.92E-20
	KEGG	AGE-RAGE signaling pathway in diabet	KEGG:04933	8.97E-15
	KEGG	Pancreatic cancer	KEGG:05212	1.40E-14
	REAC	Signaling by Interleukins	REAC:R-HSA-449147	9.04E-14
	REAC	Interleukin-4 and Interleukin-13 signal	REAC:R-HSA-6785807	1.03E-13
	REAC	Cytokine Signaling in Immune system	REAC:R-HSA-1280215	4.28E-13
	KEGG	Chronic myeloid leukemia	KEGG:05220	8.37E-13
	KEGG	Pathways in cancer	KEGG:05200	4.59E-12
	KEGG	Prostate cancer	KEGG:05215	1.37E-11
	KEGG	Non-small cell lung cancer	KEGG:05223	2.46E-11
	KEGG	Melanoma	KEGG:05218	2.46E-11
	KEGG	PI3K-Akt signaling pathway	KEGG:04151	2.77E-11
	KEGG	Hepatitis B	KEGG:05161	1.49E-10
	KEGG	FoxO signaling pathway	KEGG:04068	3.97E-10
	KEGG	Endocrine resistance	KEGG:01522	4.34E-10
	KEGG	Signaling pathways regulating pluripot	KEGG:04550	1.04E-09
	KEGG	MicroRNAs in cancer	KEGG:05206	1.35E-09
	KEGG	Gastric cancer	KEGG:05226	1.52E-09
	KEGG	Cellular senescence	KEGG:04218	2.71E-09
	KEGG	Hepatitis C	KEGG:05160	2.90E-09
	KEGG	Influenza A	KEGG:05164	6.07E-09
	KEGG	Human papillomavirus infection	KEGG:05165	4.92E-08
	REAC	Diseases of signal transduction by grov	REAC:R-HSA-5663202	5.25E-08
	KEGG	Lipid and atherosclerosis	KEGG:05417	8.20E-08
	KEGG	Hepatocellular carcinoma	KEGG:05225	1.16E-07
	KEGG	Kaposi sarcoma-associated herpesviru	KEGG:05167	5.28E-07
	KEGG	Epstein-Barr virus infection	KEGG:05169	6.43E-07
	KEGG	Viral carcinogenesis	KEGG:05203	7.08E-07
	KEGG	Human cytomegalovirus infection	KEGG:05163	2.0131E-06
	KEGG	MAPK signaling pathway	KEGG:04010	2.3023E-06

	REAC	Developmental Biology	REAC:R-HSA-1266738	3.0216E-06
	REAC	Disease	REAC:R-HSA-1643685	7.7149E-06
	REAC	Immune System	REAC:R-HSA-168256	1.5239E-05
	REAC	Generic Transcription Pathway	REAC:R-HSA-212436	0.0005226
	REAC	Signal Transduction	REAC:R-HSA-162582	0.00071959
	REAC	RNA Polymerase II Transcription	REAC:R-HSA-73857	0.00171231
	REAC	Gene expression (Transcription)	REAC:R-HSA-74160	0.00561575
	REAC	Cytokine Signaling in Immune system	REAC:R-HSA-1280215	1.70E-12
	KEGG	Measles	KEGG:05162	1.05E-11
	KEGG	Pathways in cancer	KEGG:05200	2.75E-10
	REAC	Immune System	REAC:R-HSA-168256	3.14E-08
	REAC	Disease	REAC:R-HSA-1643685	5.29E-08
	KEGG	Human cytomegalovirus infection	KEGG:05163	7.20E-08
	KEGG	Kaposi sarcoma-associated herpesviru	KEGG:05167	2.35E-07
	KEGG	PI3K-Akt signaling pathway	KEGG:04151	1.2881E-06
	REAC	Interferon Signaling	REAC:R-HSA-913531	1.3732E-06
	KEGG	Epstein-Barr virus infection	KEGG:05169	4.1308E-06
7	KEGG	MAPK signaling pathway	KEGG:04010	1.7374E-05
	REAC	PIP3 activates AKT signaling	REAC:R-HSA-1257604	1.7635E-05
	REAC	Intracellular signaling by second messe	REAC:R-HSA-9006925	7.0246E-05
	REAC	Signaling by Receptor Tyrosine Kinases	REAC:R-HSA-9006934	0.00018254
	KEGG	Herpes simplex virus 1 infection	KEGG:05168	0.00055668
	REAC	Signaling by Interleukins	REAC:R-HSA-449147	0.00071439
	REAC	Diseases of signal transduction by grov	REAC:R-HSA-5663202	0.00180379
	REAC	Signal Transduction	REAC:R-HSA-162582	0.00307621
	REAC	SARS-CoV Infections	REAC:R-HSA-9679506	0.00420563
	REAC	Infectious disease	REAC:R-HSA-5663205	0.00882985
	REAC	Viral Infection Pathways	REAC:R-HSA-9824446	0.02300685
