# Supplementary material for: Exploring molecular targets: herbal isolates in cervical cancer therapy
Source: Genomics Inform. 2024 Jun 26;22:9. doi: 10.1186/s44342-024-00008-1 (PMC11201312; doi:10.1186/s44342-024-00008-1)
Supplement: Supplementary file 5 — Additional file 5: Table S4. Biological processes enriched in cervical cancer. [file 44342_2024_8_MOESM5_ESM.pdf]

Cluster no.	Term name	Term ID	FDR
	cell cycle process	GO:0022402	9.18E-60
	mitotic cell cycle process	GO:1903047	4.20E-59
	mitotic cell cycle	GO:0000278	7.81E-55
	cell cycle	GO:0007049	3.23E-54
	nuclear division	GO:0000280	1.36E-53
	chromosome segregation	GO:0007059	9.21E-53
	nuclear chromosome segregation	GO:0098813	4.35E-52
	organelle fission	GO:0048285	1.57E-51
	mitotic nuclear division	GO:0140014	3.31E-50
	sister chromatid segregation	GO:0000819	1.98E-47
	mitotic sister chromatid segregation	GO:0000070	1.50E-46
	chromosome organization	GO:0051276	2.60E-46
	regulation of cell cycle	GO:0051726	5.52E-45
	regulation of cell cycle process	GO:0010564	8.67E-44
	cell division	GO:0051301	4.58E-42
	mitotic cell cycle phase transition	GO:0044772	7.59E-32
	cell cycle phase transition	GO:0044770	1.85E-30
	regulation of chromosome segregatio	GO:0051983	4.01E-29
	regulation of mitotic nuclear division	GO:0007088	3.81E-28
	regulation of nuclear division	GO:0051783	6.32E-28
	organelle organization	GO:0006996	1.34E-27
	regulation of mitotic cell cycle	GO:0007346	2.11E-27
	positive regulation of cell cycle proces	GO:0090068	2.23E-27
	microtubule cytoskeleton organizatioi	GO:1902850	2.24E-26
	regulation of mitotic cell cycle phase t	GO:1901990	5.39E-26
	positive regulation of cell cycle	GO:0045787	1.73E-25
	spindle organization	GO:0007051	1.50E-24
	regulation of cell cycle phase transitio	GO:1901987	1.71E-24
	regulation of chromosome separation	GO:1905818	2.57E-24
	regulation of chromosome organizatic	GO:0033044	4.19E-24
	microtubule cytoskeleton organizatioi	GO:0000226	1.04E-23
	chromosome separation	GO:0051304	1.45E-23
	regulation of sister chromatid segrega	GO:0033045	2.28E-23
	mitotic spindle organization	GO:0007052	4.46E-23
	regulation of mitotic metaphase/anap	GO:0030071	1.31E-22
	regulation of mitotic sister chromatid	GO:0033047	1.40E-22
	regulation of organelle organization	GO:0033043	1.62E-22
	regulation of metaphase/anaphase tra	GO:1902099	2.39E-22
	negative regulation of mitotic nuclear	GO:0045839	2.66E-22
	metaphase/anaphase transition of mi	GO:0007091	2.91E-22
	metaphase/anaphase transition of cel	GO:0044784	5.19E-22
	regulation of mitotic sister chromatid	GO:0010965	6.61E-22
	mitotic spindle checkpoint signaling	GO:0071174	1.43E-21
	spindle assembly checkpoint signaling	GO:0071173	1.43E-21
	mitotic spindle assembly checkpoint s	GO:0007094	1.43E-21
	mitotic sister chromatid separation	GO:0051306	1.55E-21

negative regulation of nuclear divisior	GO:0051784	2.05E-21
spindle checkpoint signaling	GO:0031577	2.05E-21
negative regulation of sister chromati	GO:0033046	2.91E-21
negative regulation of mitotic sister cl	GO:2000816	2.91E-21
negative regulation of mitotic metaph	GO:0045841	2.91E-21
negative regulation of mitotic sister cl	GO:0033048	2.91E-21
negative regulation of chromosome s	GO:1905819	5.73E-21
negative regulation of chromosome s	GO:0051985	5.73E-21
negative regulation of metaphase/ana	GO:1902100	5.73E-21
mitotic cell cycle checkpoint signaling	GO:0007093	8.76E-21
chromosome localization	GO:0050000	9.80E-21
metaphase chromosome alignment	GO:0051310	2.04E-20
microtubule-based process	GO:0007017	2.50E-20
negative regulation of chromosome o	GO:2001251	2.95E-20
cell cycle checkpoint signaling	GO:0000075	6.09E-20
spindle assembly	GO:0051225	1.19E-19
establishment of chromosome localiza	GO:0051303	1.92E-19
negative regulation of mitotic cell cycl	GO:1901991	6.60E-19
non-membrane-bounded organelle as	GO:0140694	4.76E-18
negative regulation of cell cycle proce	GO:0010948	9.35E-18
negative regulation of cell cycle phase	GO:1901988	1.53E-17
attachment of spindle microtubules to	GO:0008608	2.40E-17
negative regulation of mitotic cell cycl	GO:0045930	5.56E-17
cytoskeleton organization	GO:0007010	7.54E-16
negative regulation of cell cycle	GO:0045786	1.26E-15
meiotic cell cycle	GO:0051321	2.26E-15
cell cycle G2/M phase transition	GO:0044839	4.30E-15
regulation of cellular component orga	GO:0051128	5.98E-15
meiotic cell cycle process	GO:1903046	1.32E-14
mitotic spindle assembly	GO:0090307	2.31E-14
mitotic metaphase chromosome align	GO:0007080	8.86E-14
meiotic nuclear division	GO:0140013	8.99E-14
cytokinesis	GO:0000910	9.74E-14
negative regulation of organelle orgar	GO:0010639	1.61E-13
G2/M transition of mitotic cell cycle	GO:0000086	1.47E-12
cellular component organization	GO:0016043	6.93E-12
organelle localization	GO:0051640	7.99E-12
mitotic cytokinesis	GO:0000281	1.31E-11
regulation of cytokinesis	GO:0032465	1.69E-11
establishment of organelle localizatio	GO:0051656	1.88E-11
DNA metabolic process	GO:0006259	2.50E-11
cellular component organization or bi	GO:0071840	2.66E-11
cytoskeleton-dependent cytokinesis	GO:0061640	3.50E-10
organelle assembly	GO:0070925	6.44E-10
regulation of cell division	GO:0051302	1.73E-09
negative regulation of cellular compo	GO:0051129	1.93E-09
regulation of G2/M transition of mito	GO:0010389	2.33E-09

1

regulation of cell cycle G2/M phase tr	GO:1902749	7.30E-09
positive regulation of mitotic cell cycl	GO:0045931	1.23E-08
meiosis I cell cycle process	GO:0061982	4.37E-08
DNA replication	GO:0006260	2.47E-07
sexual reproduction	GO:0019953	2.80E-07
DNA repair	GO:0006281	1.82016E-06
regulation of transferase activity	GO:0051338	2.50157E-06
DNA damage response	GO:0006974	6.64693E-06
protein phosphorylation	GO:0006468	1.56624E-05
reproductive process	GO:0022414	2.09691E-05
reproduction	GO:0000003	2.35263E-05
phosphorylation	GO:0016310	4.11631E-05
intracellular signal transduction	GO:0035556	4.45812E-05
regulation of cellular process	GO:0050794	0.000174996
positive regulation of organelle organ	GO:0010638	0.000182577
regulation of DNA metabolic process	GO:0051052	0.000300674
regulation of biological process	GO:0050789	0.000373574
positive regulation of cellular process	GO:0048522	0.000787367
biological regulation	GO:0065007	0.001892153
microtubule-based movement	GO:0007018	0.002323195
positive regulation of biological proce	GO:0048518	0.00300106
regulation of protein modification pro	GO:0031399	0.003773295
phosphate-containing compound met	GO:0006796	0.00576019
phosphorus metabolic process	GO:0006793	0.006783209
regulation of catalytic activity	GO:0050790	0.007027181
positive regulation of transferase acti	GO:0051347	0.015300009
cellular component assembly	GO:0022607	0.018071685
gamete generation	GO:0007276	0.019283702
cellular response to stress	GO:0033554	0.020477439
protein modification process	GO:0036211	0.033583341
establishment of localization in cell	GO:0051649	0.036578634
cellular localization	GO:0051641	0.037844996
regulation of protein phosphorylation	GO:0001932	0.046711891
cellular response to stress	GO:0033554	1.21E-16
cell cycle	GO:0007049	8.16E-16
DNA metabolic process	GO:0006259	1.03E-13
DNA damage response	GO:0006974	1.72E-13
cell cycle process	GO:0022402	1.92E-13
response to abiotic stimulus	GO:0009628	7.63E-12
response to stress	GO:0006950	1.12E-11
regulation of cell population prolifera	GO:0042127	2.44E-11
response to radiation	GO:0009314	6.00E-11
regulation of cell cycle	GO:0051726	1.72E-10
chromosome organization	GO:0051276	3.94E-10
apoptotic process	GO:0006915	4.50E-10
regulation of nitrogen compound met	GO:0051171	4.58E-10
regulation of molecular function	GO:0065009	5.03E-10

regulation of response to stress	GO:0080134	6.37E-10
programmed cell death	GO:0012501	9.03E-10
cell death	GO:0008219	9.56E-10
intracellular signal transduction	GO:0035556	9.80E-10
regulation of DNA metabolic process	GO:0051052	9.93E-10
regulation of primary metabolic proce	GO:0080090	1.09E-09
positive regulation of cell population	GO:0008284	1.12E-09
positive regulation of nitrogen compo	GO:0051173	2.21E-09
regulation of cellular component orga	GO:0051128	2.94E-09
regulation of protein modification pro	GO:0031399	5.99E-09
cell population proliferation	GO:0008283	6.02E-09
transmembrane receptor protein tyro	GO:0007169	6.21E-09
response to endogenous stimulus	GO:0009719	1.15E-08
regulation of apoptotic process	GO:0042981	1.44E-08
regulation of transferase activity	GO:0051338	1.63E-08
regulation of immune system process	GO:0002682	1.77E-08
negative regulation of nitrogen comp	GO:0051172	2.23E-08
regulation of programmed cell death	GO:0043067	2.31E-08
animal organ development	GO:0048513	2.70E-08
regulation of catalytic activity	GO:0050790	2.80E-08
regulation of protein metabolic proce	GO:0051246	2.95E-08
positive regulation of macromolecule	GO:0010604	3.24E-08
regulation of cell cycle process	GO:0010564	4.63E-08
organelle organization	GO:0006996	5.21E-08
response to light stimulus	GO:0009416	6.49E-08
negative regulation of cellular process	GO:0048523	6.97E-08
regulation of nucleobase-containing c	GO:0019219	7.04E-08
cellular response to stimulus	GO:0051716	7.07E-08
cellular response to environmental sti	GO:0104004	8.04E-08
cellular response to abiotic stimulus	GO:0071214	8.04E-08
regulation of organelle organization	GO:0033043	8.94E-08
regulation of macromolecule metabol	GO:0060255	9.05E-08
enzyme-linked receptor protein signal	GO:0007167	1.06E-07
regulation of cellular localization	GO:0060341	1.47E-07
negative regulation of macromolecule	GO:0010605	1.49E-07
regulation of multicellular organismal	GO:0051239	1.56E-07
cellular response to endogenous stim	GO:0071495	1.86E-07
positive regulation of metabolic proce	GO:0009893	2.00E-07
regulation of protein phosphorylation	GO:0001932	2.19E-07
cellular response to chemical stimulus	GO:0070887	2.63E-07
reproductive process	GO:0022414	3.39E-07
regulation of response to stimulus	GO:0048583	3.68E-07
reproduction	GO:0000003	3.74E-07
negative regulation of biological proce	GO:0048519	4.04E-07
regulation of protein localization	GO:0032880	5.45E-07
negative regulation of metabolic proc	GO:0009892	5.47E-07
positive regulation of RNA metabolic	GO:0051254	5.63E-07

positive regulation of catalytic activity	GO:0043085	5.72E-07
regulation of developmental process	GO:0050793	5.76E-07
positive regulation of protein modifica	GO:0031401	6.26E-07
response to growth factor	GO:0070848	6.82E-07
regulation of metabolic process	GO:0019222	6.82E-07
cell cycle phase transition	GO:0044770	7.77E-07
regulation of phosphorylation	GO:0042325	7.80E-07
cellular component organization	GO:0016043	9.18E-07
regulation of cellular metabolic proce	GO:0031323	9.47E-07
positive regulation of protein metabo	GO:0051247	9.94E-07
epithelial cell proliferation	GO:0050673	1.63323E-06
regulation of intracellular signal trans	GO:1902531	1.66927E-06
gland development	GO:0048732	1.78237E-06
positive regulation of DNA-templated	GO:0045893	1.78903E-06
cellular component organization or bi	GO:0071840	1.8971E-06
positive regulation of RNA biosyntheti	GO:1902680	1.9003E-06
lymphocyte activation	GO:0046649	1.90567E-06
DNA repair	GO:0006281	1.97301E-06
tissue development	GO:0009888	2.03975E-06
positive regulation of cellular metabol	GO:0031325	2.15941E-06
positive regulation of nucleobase-con	GO:0045935	2.66657E-06
protein phosphorylation	GO:0006468	3.13974E-06
positive regulation of transcription by	GO:0045944	3.20491E-06
regulation of cell-cell adhesion	GO:0022407	3.27922E-06
immune system process	GO:0002376	3.34167E-06
positive regulation of molecular funct	GO:0044093	3.34892E-06
response to oxygen-containing compo	GO:1901700	3.36761E-06
positive regulation of protein localizat	GO:1903829	3.70169E-06
regulation of phosphate metabolic pro	GO:0019220	3.78164E-06
regulation of phosphorus metabolic p	GO:0051174	3.82066E-06
cell development	GO:0048468	4.07801E-06
cellular response to oxygen-containin	GO:1901701	4.1043E-06
system development	GO:0048731	4.23963E-06
regulation of cell development	GO:0060284	4.36691E-06
cell surface receptor signaling pathwa	GO:0007166	4.64074E-06
regulation of cellular response to stre	GO:0080135	5.00674E-06
regulation of lymphocyte activation	GO:0051249	5.17368E-06
regulation of cell differentiation	GO:0045595	5.84617E-06
cell activation	GO:0001775	6.75666E-06
cellular response to growth factor stir	GO:0071363	6.94692E-06
macromolecule modification	GO:0043412	7.03416E-06
negative regulation of apoptotic proce	GO:0043066	7.98708E-06
positive regulation of transferase acti	GO:0051347	8.43777E-06
positive regulation of response to stin	GO:0048584	9.33377E-06
regulation of kinase activity	GO:0043549	9.67884E-06
response to inorganic substance	GO:0010035	9.75728E-06
multicellular organism development	GO:0007275	1.11237E-05

	negative regulation of programmed ce	GO:0043069	1.12066E-05
	positive regulation of macromolecule	GO:0010557	1.31561E-05
	regulation of RNA metabolic process	GO:0051252	1.32321E-05
	T cell activation	GO:0042110	1.36524E-05
	regulation of multicellular organismal	GO:2000026	1.40216E-05
2	growth	GO:0040007	1.46505E-05
	positive regulation of biological proce	GO:0048518	1.50115E-05
	response to stimulus	GO:0050896	1.55243E-05
	positive regulation of cellular process	GO:0048522	1.60006E-05
	leukocyte activation	GO:0045321	1.63155E-05
	cell-cell adhesion	GO:0098609	1.63155E-05
	hemopoiesis	GO:0030097	1.88008E-05
	negative regulation of molecular func	GO:0044092	1.99335E-05
	regulation of DNA-templated transcrip	GO:0006355	2.14866E-05
	anatomical structure morphogenesis	GO:0009653	2.19154E-05
	positive regulation of cellular biosynth	GO:0031328	2.20465E-05
	positive regulation of intracellular sigr	GO:1902533	2.3984E-05
	regulation of RNA biosynthetic proces	GO:2001141	2.41726E-05
	phosphorylation	GO:0016310	2.45077E-05
	positive regulation of biosynthetic pro	GO:0009891	2.468E-05
	regulation of leukocyte activation	GO:0002694	2.49677E-05
	signaling	GO:0023052	2.53704E-05
	regulation of transcription by RNA pol	GO:0006357	3.39741E-05
	regulation of protein kinase activity	GO:0045859	3.48692E-05
	cell communication	GO:0007154	3.61928E-05
	signal transduction	GO:0007165	3.62953E-05
	regulation of catabolic process	GO:0009894	3.79628E-05
	positive regulation of signal transduct	GO:0009967	3.81783E-05
	response to chemical	GO:0042221	3.83484E-05
	apoptotic signaling pathway	GO:0097190	3.88687E-05
	DNA-templated transcription	GO:0006351	4.05188E-05
	response to organonitrogen compoun	GO:0010243	4.61463E-05
	RNA biosynthetic process	GO:0032774	4.85734E-05
	regulation of cell activation	GO:0050865	5.50255E-05
	negative regulation of nucleobase-cor	GO:0045934	5.54034E-05
	cellular response to organonitrogen co	GO:0071417	5.58462E-05
	negative regulation of cellular metabo	GO:0031324	5.81755E-05
	sexual reproduction	GO:0019953	6.74399E-05
	transcription by RNA polymerase II	GO:0006366	6.78264E-05
	regulation of cellular catabolic proces	GO:0031329	7.05362E-05
	positive regulation of protein phospho	GO:0001934	8.48904E-05
	cellular response to nitrogen compou	GO:1901699	9.76323E-05
	negative regulation of cell differentiat	GO:0045596	9.76323E-05
	response to nitrogen compound	GO:1901698	9.91583E-05
	regulation of signal transduction	GO:0009966	0.000111821
	nucleic acid metabolic process	GO:0090304	0.000126184
	regulation of signaling	GO:0023051	0.000131637

regulation of cell communication	GO:0010646	0.000137522
mitotic cell cycle	GO:0000278	0.000138275
protein modification process	GO:0036211	0.000140216
negative regulation of developmental	GO:0051093	0.000163296
cellular response to organic substance	GO:0071310	0.000166523
cell differentiation	GO:0030154	0.000168433
cellular developmental process	GO:0048869	0.000169143
response to external stimulus	GO:0009605	0.000174966
positive regulation of phosphorylation	GO:0042327	0.000195198
positive regulation of signaling	GO:0023056	0.000204231
positive regulation of cell communicat	GO:0010647	0.000204231
anatomical structure development	GO:0048856	0.000210297
mitotic cell cycle process	GO:1903047	0.000280727
response to organic substance	GO:0010033	0.000283715
epithelium development	GO:0060429	0.000328506
regulation of cell adhesion	GO:0030155	0.000336776
nucleobase-containing compound bio	GO:0034654	0.000338369
regulation of localization	GO:0032879	0.000364604
heterocycle biosynthetic process	GO:0018130	0.000460386
positive regulation of phosphate meta	GO:0045937	0.000468289
positive regulation of phosphorus met	GO:0010562	0.000468289
aromatic compound biosynthetic proc	GO:0019438	0.000481974
nucleobase-containing compound me	GO:0006139	0.000601035
peptidyl-amino acid modification	GO:0018193	0.00077124
tube development	GO:0035295	0.000822704
organic cyclic compound biosynthetic	GO:1901362	0.000869872
negative regulation of response to sti	GO:0048585	0.000909889
tube morphogenesis	GO:0035239	0.00098146
heterocycle metabolic process	GO:0046483	0.000986426
regulation of biological process	GO:0050789	0.001018995
cellular aromatic compound metaboli	GO:0006725	0.001144911
cell motility	GO:0048870	0.001175671
developmental process	GO:0032502	0.00133835
multicellular organismal reproductive	GO:0048609	0.001438102
cellular nitrogen compound biosynthe	GO:0044271	0.001448704
protein metabolic process	GO:0019538	0.001517516
positive regulation of gene expression	GO:0010628	0.001956771
organic cyclic compound metabolic pr	GO:1901360	0.002237281
cell migration	GO:0016477	0.002249331
regulation of gene expression	GO:0010468	0.002327767
multicellular organism reproduction	GO:0032504	0.002346346
cell adhesion	GO:0007155	0.00276426
biological regulation	GO:0065007	0.002845933
negative regulation of protein metabo	GO:0051248	0.003020558
regulation of cellular process	GO:0050794	0.003097473
developmental process involved in re	GO:0003006	0.003340505
cellular nitrogen compound metaboli	GO:0034641	0.003589913

	regulation of macromolecule biosynth	GO:0010556	0.003596776
	regulation of cellular biosynthetic pro	GO:0031326	0.005383984
	catabolic process	GO:0009056	0.005440843
	animal organ morphogenesis	GO:0009887	0.005567868
	regulation of biosynthetic process	GO:0009889	0.005954072
	positive regulation of multicellular org	GO:0051240	0.006395301
	nitrogen compound metabolic proces	GO:0006807	0.007268711
	phosphate-containing compound met	GO:0006796	0.007889338
	phosphorus metabolic process	GO:0006793	0.008862644
	positive regulation of cellular compon	GO:0051130	0.009216575
	negative regulation of macromolecule	GO:0010558	0.009807446
	circulatory system development	GO:0072359	0.009823343
	embryo development	GO:0009790	0.009901583
	macromolecule metabolic process	GO:0043170	0.011910221
	negative regulation of cellular biosynt	GO:0031327	0.012467633
	negative regulation of biosynthetic pr	GO:0009890	0.013271213
	regulation of transport	GO:0051049	0.014429525
	anatomical structure formation involv	GO:0048646	0.014796995
	regulation of biological quality	GO:0065008	0.02139886
	locomotion	GO:0040011	0.021831579
	primary metabolic process	GO:0044238	0.025743047
	negative regulation of signal transduc	GO:0009968	0.029514778
	organonitrogen compound metabolic	GO:1901564	0.032193331
	positive regulation of developmental	GO:0051094	0.039179573
	multicellular organismal process	GO:0032501	0.0438453
	cellular metabolic process	GO:0044237	0.043971151
3	cell division	GO:0051301	2.21E-13
	nuclear division	GO:0000280	6.76E-11
	organelle fission	GO:0048285	2.32E-10
	regulation of cell cycle process	GO:0010564	2.44E-08
	regulation of cell cycle	GO:0051726	3.55879E-06
	DNA metabolic process	GO:0006259	2.57785E-05
	DNA-templated DNA replication	GO:0006261	4.06E-12
	sister chromatid segregation	GO:0000819	1.43E-10
	mitotic nuclear division	GO:0140014	9.95E-10
	DNA replication	GO:0006260	1.11E-09
	nuclear chromosome segregation	GO:0098813	3.24E-09
	chromosome segregation	GO:0007059	5.00E-08
	DNA damage response	GO:0006974	8.16771E-05
	regulation of organelle organization	GO:0033043	0.000940005
	positive regulation of cell population	GO:0008284	7.50E-21
	regulation of programmed cell death	GO:0043067	2.84E-20
	regulation of apoptotic process	GO:0042981	4.33E-19
	regulation of cell population prolifera	GO:0042127	7.02E-19
	regulation of developmental process	GO:0050793	7.45E-19
	programmed cell death	GO:0012501	1.41E-18
	cell death	GO:0008219	1.53E-18

negative regulation of developmental	GO:0051093	8.76E-18
apoptotic process	GO:0006915	1.47E-17
negative regulation of cell differentiat	GO:0045596	1.65E-17
system development	GO:0048731	4.83E-17
regulation of cell differentiation	GO:0045595	6.29E-17
regulation of multicellular organismal	GO:0051239	1.67E-16
multicellular organism development	GO:0007275	2.24E-16
positive regulation of cellular metabol	GO:0031325	3.90E-16
circulatory system development	GO:0072359	5.50E-16
cell surface receptor signaling pathwa	GO:0007166	6.43E-16
positive regulation of macromolecule	GO:0010604	7.56E-16
cell differentiation	GO:0030154	7.94E-16
cellular developmental process	GO:0048869	8.00E-16
tissue development	GO:0009888	1.22E-15
cell population proliferation	GO:0008283	1.75E-15
positive regulation of macromolecule	GO:0010557	2.20E-15
locomotion	GO:0040011	3.80E-15
positive regulation of cellular biosynth	GO:0031328	5.22E-15
positive regulation of biosynthetic prc	GO:0009891	6.31E-15
positive regulation of cell migration	GO:0030335	9.61E-15
positive regulation of metabolic proce	GO:0009893	1.00E-14
positive regulation of developmental	GO:0051094	1.58E-14
positive regulation of cell motility	GO:2000147	2.13E-14
regulation of cell motility	GO:2000145	2.78E-14
positive regulation of locomotion	GO:0040017	3.32E-14
regulation of locomotion	GO:0040012	6.11E-14
negative regulation of cellular process	GO:0048523	8.39E-14
anatomical structure morphogenesis	GO:0009653	1.00E-13
cell motility	GO:0048870	1.58E-13
positive regulation of nitrogen compo	GO:0051173	1.92E-13
cell migration	GO:0016477	1.95E-13
regulation of cell migration	GO:0030334	2.03E-13
developmental process	GO:0032502	4.60E-13
response to stress	GO:0006950	4.69E-13
epithelial cell proliferation	GO:0050673	5.12E-13
anatomical structure development	GO:0048856	6.45E-13
anatomical structure formation involv	GO:0048646	7.52E-13
negative regulation of biological proce	GO:0048519	8.58E-13
intracellular signal transduction	GO:0035556	8.61E-13
positive regulation of gene expression	GO:0010628	9.00E-13
signal transduction	GO:0007165	9.22E-13
regulation of multicellular organismal	GO:2000026	1.20E-12
tube morphogenesis	GO:0035239	1.38E-12
epithelium development	GO:0060429	1.99E-12
regulation of intracellular signal trans	GO:1902531	3.43E-12
tube development	GO:0035295	3.49E-12
response to endogenous stimulus	GO:0009719	3.65E-12

regulation of molecular function	GO:0065009	6.26E-12
positive regulation of cellular process	GO:0048522	6.31E-12
signaling	GO:0023052	1.14E-11
cell communication	GO:0007154	1.88E-11
regulation of signal transduction	GO:0009966	2.11E-11
positive regulation of cell differentiati	GO:0045597	2.18E-11
blood vessel development	GO:0001568	2.46E-11
response to growth factor	GO:0070848	2.57E-11
negative regulation of cell population	GO:0008285	2.90E-11
regulation of epithelial cell proliferatic	GO:0050678	4.19E-11
animal organ development	GO:0048513	4.55E-11
vasculature development	GO:0001944	4.58E-11
cellular response to stimulus	GO:0051716	6.05E-11
negative regulation of programmed ce	GO:0043069	6.28E-11
neurogenesis	GO:0022008	6.69E-11
positive regulation of biological proce	GO:0048518	1.10E-10
multicellular organismal process	GO:0032501	1.87E-10
regulation of cell development	GO:0060284	3.19E-10
ameboidal-type cell migration	GO:0001667	3.30E-10
regulation of signaling	GO:0023051	5.31E-10
regulation of cell communication	GO:0010646	5.66E-10
glial cell differentiation	GO:0010001	5.70E-10
cell development	GO:0048468	6.58E-10
negative regulation of apoptotic proce	GO:0043066	7.26E-10
cellular response to endogenous stim	GO:0071495	7.36E-10
regulation of nitrogen compound met	GO:0051171	8.77E-10
heart development	GO:0007507	9.70E-10
nervous system development	GO:0007399	1.02E-09
cell activation	GO:0001775	1.08E-09
phosphorylation	GO:0016310	1.27E-09
response to stimulus	GO:0050896	1.56E-09
leukocyte differentiation	GO:0002521	1.69E-09
blood vessel morphogenesis	GO:0048514	1.69E-09
response to chemical	GO:0042221	2.12E-09
regulation of primary metabolic proce	GO:0080090	2.19E-09
tissue morphogenesis	GO:0048729	2.29E-09
muscle cell proliferation	GO:0033002	2.67E-09
enzyme-linked receptor protein signal	GO:0007167	2.68E-09
negative regulation of transcription by	GO:0000122	3.56E-09
morphogenesis of an epithelium	GO:0002009	4.15E-09
cellular response to chemical stimulus	GO:0070887	4.38E-09
angiogenesis	GO:0001525	4.67E-09
regulation of transcription by RNA pol	GO:0006357	4.97E-09
positive regulation of multicellular org	GO:0051240	5.28E-09
cellular response to growth factor stir	GO:0071363	5.88E-09
regulation of gene expression	GO:0010468	7.04E-09
response to wounding	GO:0009611	7.28E-09

regulation of DNA-templated transcrip	GO:0006355	7.99E-09
lymphocyte differentiation	GO:0030098	9.21E-09
regulation of RNA biosynthetic proces	GO:2001141	9.31E-09
positive regulation of response to stin	GO:0048584	1.05E-08
positive regulation of DNA-templated	GO:0045893	1.11E-08
positive regulation of RNA biosyntheti	GO:1902680	1.19E-08
transcription by RNA polymerase II	GO:0006366	1.26E-08
regulation of macromolecule biosynth	GO:0010556	1.36E-08
regulation of metabolic process	GO:0019222	1.37E-08
gliogenesis	GO:0042063	1.37E-08
cellular response to oxygen-containin	GO:1901701	1.38E-08
positive regulation of protein modifica	GO:0031401	1.43E-08
positive regulation of signaling	GO:0023056	1.45E-08
positive regulation of cell communicat	GO:0010647	1.45E-08
positive regulation of phosphorylatior	GO:0042327	1.50E-08
protein phosphorylation	GO:0006468	1.50E-08
regulation of response to stimulus	GO:0048583	1.53E-08
regulation of cellular metabolic proce	GO:0031323	1.56E-08
positive regulation of molecular funct	GO:0044093	1.62E-08
positive regulation of signal transduct	GO:0009967	1.81E-08
DNA-templated transcription	GO:0006351	1.82E-08
regulation of macromolecule metabol	GO:0060255	1.91E-08
negative regulation of DNA-templatec	GO:0045892	2.26E-08
RNA biosynthetic process	GO:0032774	2.30E-08
regulation of cellular biosynthetic pro	GO:0031326	2.50E-08
positive regulation of nucleobase-con	GO:0045935	2.54E-08
regulation of phosphorylation	GO:0042325	2.64E-08
negative regulation of RNA biosynthet	GO:1902679	2.67E-08
regulation of catalytic activity	GO:0050790	2.72E-08
embryo development	GO:0009790	2.75E-08
regulation of biosynthetic process	GO:0009889	2.91E-08
mononuclear cell differentiation	GO:1903131	3.29E-08
hemopoiesis	GO:0030097	3.65E-08
regulation of protein metabolic proce	GO:0051246	4.62E-08
positive regulation of RNA metabolic	GO:0051254	4.71E-08
positive regulation of protein metabo	GO:0051247	4.86E-08
positive regulation of intracellular sigr	GO:1902533	4.97E-08
positive regulation of phosphate meta	GO:0045937	5.25E-08
positive regulation of phosphorus met	GO:0010562	5.25E-08
regulation of protein modification pro	GO:0031399	5.52E-08
regulation of RNA metabolic process	GO:0051252	5.90E-08
regulation of angiogenesis	GO:0045765	6.61E-08
regulation of vasculature developmen	GO:1901342	7.33E-08
positive regulation of protein phosphc	GO:0001934	8.29E-08
regulation of transferase activity	GO:0051338	9.11E-08
negative regulation of RNA metabolic	GO:0051253	9.97E-08
regulation of hemopoiesis	GO:1903706	1.17E-07

response to decreased oxygen levels	GO:0036293	1.21E-07
positive regulation of transcription by	GO:0045944	1.34E-07
epithelial cell migration	GO:0010631	1.38E-07
animal organ morphogenesis	GO:0009887	1.47E-07
epithelium migration	GO:0090132	1.52E-07
T cell differentiation	GO:0030217	1.52E-07
regulation of kinase activity	GO:0043549	1.56E-07
regulation of phosphate metabolic pro	GO:0019220	1.61E-07
regulation of phosphorus metabolic p	GO:0051174	1.63E-07
mesenchyme development	GO:0060485	1.78E-07
tissue migration	GO:0090130	1.84E-07
nucleobase-containing compound bio	GO:0034654	2.86E-07
response to oxygen levels	GO:0070482	2.99E-07
regulation of nucleobase-containing c	GO:0019219	3.06E-07
negative regulation of nucleobase-cor	GO:0045934	3.39E-07
heterocycle biosynthetic process	GO:0018130	4.28E-07
leukocyte activation	GO:0045321	4.34E-07
aromatic compound biosynthetic proc	GO:0019438	4.54E-07
apoptotic signaling pathway	GO:0097190	5.25E-07
transmembrane receptor protein tyro	GO:0007169	5.34E-07
embryonic morphogenesis	GO:0048598	5.44E-07
lymphocyte activation	GO:0046649	5.45E-07
response to organic substance	GO:0010033	7.92E-07
positive regulation of cytokine produc	GO:0001819	7.92E-07
response to peptide	GO:1901652	9.20E-07
organic cyclic compound biosynthetic	GO:1901362	9.82E-07
multicellular organismal-level homeos	GO:0048871	1.00802E-06
regulation of protein phosphorylation	GO:0001932	1.18417E-06
positive regulation of apoptotic proce	GO:0043065	1.23243E-06
response to organonitrogen compoun	GO:0010243	1.46989E-06
positive regulation of transferase activ	GO:0051347	1.54164E-06
central nervous system development	GO:0007417	1.54571E-06
regulation of immune system process	GO:0002682	1.74053E-06
positive regulation of programmed ce	GO:0043068	1.84464E-06
negative regulation of signal transduc	GO:0009968	2.02584E-06
regulation of anatomical structure mo	GO:0022603	2.07889E-06
response to oxygen-containing compo	GO:1901700	2.60595E-06
T cell activation	GO:0042110	2.61586E-06
positive regulation of catalytic activity	GO:0043085	3.05865E-06
negative regulation of nitrogen compo	GO:0051172	3.0969E-06
epithelial cell differentiation	GO:0030855	3.46786E-06
negative regulation of multicellular or	GO:0051241	3.48165E-06
response to nitrogen compound	GO:1901698	3.60595E-06
response to abiotic stimulus	GO:0009628	3.64828E-06
response to external stimulus	GO:0009605	3.68402E-06
positive regulation of kinase activity	GO:0033674	3.80974E-06
phosphate-containing compound met	GO:0006796	3.88341E-06

phosphorus metabolic process	GO:0006793	4.57349E-06
immune system process	GO:0002376	4.91206E-06
gland development	GO:0048732	5.41644E-06
negative regulation of signaling	GO:0023057	6.3368E-06
negative regulation of cell communica	GO:0010648	6.3368E-06
MAPK cascade	GO:0000165	6.53913E-06
regulation of cytokine production	GO:0001817	6.83226E-06
regulation of cell adhesion	GO:0030155	7.1372E-06
cytokine production	GO:0001816	7.45438E-06
negative regulation of response to sti	GO:0048585	7.50199E-06
homeostatic process	GO:0042592	1.31254E-05
negative regulation of cellular metabo	GO:0031324	1.34865E-05
cellular response to organic substance	GO:0071310	1.63108E-05
generation of neurons	GO:0048699	1.74978E-05
cellular nitrogen compound biosynthe	GO:0044271	1.81177E-05
muscle structure development	GO:0061061	2.0391E-05
regulation of cellular process	GO:0050794	2.23558E-05
protein metabolic process	GO:0019538	2.40399E-05
negative regulation of intracellular sig	GO:1902532	2.62254E-05
peptidyl-amino acid modification	GO:0018193	2.85497E-05
protein modification process	GO:0036211	3.54658E-05
negative regulation of macromolecule	GO:0010558	3.9889E-05
response to lipid	GO:0033993	4.2902E-05
negative regulation of cellular biosynt	GO:0031327	5.47191E-05
negative regulation of biosynthetic pr	GO:0009890	5.94142E-05
negative regulation of metabolic proc	GO:0009892	7.88108E-05
regulation of response to stress	GO:0080134	9.57063E-05
macromolecule modification	GO:0043412	0.00010904
regulation of biological process	GO:0050789	0.000117041
regulation of catabolic process	GO:0009894	0.000144338
negative regulation of macromolecule	GO:0010605	0.00016262
cellular response to organonitrogen c	GO:0071417	0.000164056
inflammatory response	GO:0006954	0.0001711
cellular response to cytokine stimulus	GO:0071345	0.00017321
chordate embryonic development	GO:0043009	0.000244675
regulation of MAPK cascade	GO:0043408	0.000255102
regulation of cellular component orga	GO:0051128	0.000274363
cellular response to nitrogen compou	GO:1901699	0.000284847
embryo development ending in birth (	GO:0009792	0.000330724
biological regulation	GO:0065007	0.000373739
regulation of cell cycle	GO:0051726	0.000396899
response to organic cyclic compound	GO:0014070	0.000469142
response to cytokine	GO:0034097	0.000495507
gene expression	GO:0010467	0.000976863
organonitrogen compound metabolic	GO:1901564	0.000997011
regulation of cellular localization	GO:0060341	0.001009909
head development	GO:0060322	0.001233958

	cell adhesion	GO:0007155	0.001389866
	regulation of localization	GO:0032879	0.001937086
	response to hormone	GO:0009725	0.00252
	regulation of protein localization	GO:0032880	0.003165413
	cellular response to stress	GO:0033554	0.003632015
	macromolecule biosynthetic process	GO:0009059	0.003963431
	regulation of biological quality	GO:0065008	0.00405414
	mitotic cell cycle	GO:0000278	0.004192825
	immune response	GO:0006955	0.004260931
	neuron differentiation	GO:0030182	0.004710849
	biological process involved in interspe	GO:0044419	0.005436438
	cell-cell adhesion	GO:0098609	0.005659654
	nitrogen compound metabolic proces	GO:0006807	0.007806913
	regulation of transport	GO:0051049	0.008191436
	cell cycle	GO:0007049	0.011079549
	response to other organism	GO:0051707	0.011230205
	response to external biotic stimulus	GO:0043207	0.011458743
	positive regulation of immune system	GO:0002684	0.013174414
	macromolecule metabolic process	GO:0043170	0.013175965
	response to biotic stimulus	GO:0009607	0.014445387
	regulation of response to external stir	GO:0032101	0.016797695
	RNA metabolic process	GO:0016070	0.028350726
	primary metabolic process	GO:0044238	0.02977435
	cellular biosynthetic process	GO:0044249	0.036033834
	organic substance biosynthetic proces	GO:1901576	0.044494721
	defense response	GO:0006952	0.049747254
	DNA metabolic process	GO:0006259	1.16E-09
	nucleic acid metabolic process	GO:0090304	0.015298106
6	nucleobase-containing compound me	GO:0006139	0.031525405
	heterocycle metabolic process	GO:0046483	0.039746618
	cellular aromatic compound metaboli	GO:0006725	0.042625092
	regulation of developmental process	GO:0050793	1.35E-17
	positive regulation of gene expression	GO:0010628	4.39E-17
	positive regulation of cellular metabol	GO:0031325	1.05E-15
	positive regulation of macromolecule	GO:0010557	1.21E-15
	regulation of cell differentiation	GO:0045595	1.97E-15
	positive regulation of macromolecule	GO:0010604	2.13E-15
	regulation of cell population prolifera	GO:0042127	2.40E-15
	defense response	GO:0006952	2.56E-15
	positive regulation of cellular biosynth	GO:0031328	3.11E-15
	response to external stimulus	GO:0009605	3.18E-15
	cellular response to organic substance	GO:0071310	3.76E-15
	positive regulation of biosynthetic prc	GO:0009891	3.83E-15
	multicellular organism development	GO:0007275	5.25E-15
	response to organic substance	GO:0010033	8.27E-15
	positive regulation of metabolic proce	GO:0009893	3.41E-14
	regulation of response to external stir	GO:0032101	3.42E-14

positive regulation of cellular process	GO:0048522	3.56E-14
response to biotic stimulus	GO:0009607	3.58E-14
regulation of multicellular organismal	GO:2000026	4.22E-14
cell population proliferation	GO:0008283	4.43E-14
cellular response to chemical stimulus	GO:0070887	5.15E-14
positive regulation of response to stin	GO:0048584	5.34E-14
response to stress	GO:0006950	8.65E-14
positive regulation of multicellular org	GO:0051240	9.26E-14
system development	GO:0048731	1.22E-13
cell surface receptor signaling pathwa	GO:0007166	1.33E-13
response to chemical	GO:0042221	1.48E-13
biological process involved in interspe	GO:0044419	2.47E-13
response to other organism	GO:0051707	3.25E-13
response to external biotic stimulus	GO:0043207	3.40E-13
regulation of defense response	GO:0031347	3.43E-13
immune response	GO:0006955	4.70E-13
regulation of multicellular organismal	GO:0051239	8.06E-13
tube development	GO:0035295	9.49E-13
positive regulation of biological proce	GO:0048518	1.04E-12
developmental process	GO:0032502	2.66E-12
regulation of immune response	GO:0050776	4.95E-12
positive regulation of immune system	GO:0002684	6.26E-12
defense response to other organism	GO:0098542	6.74E-12
programmed cell death	GO:0012501	9.24E-12
cell death	GO:0008219	9.91E-12
response to cytokine	GO:0034097	1.14E-11
immune system process	GO:0002376	1.30E-11
regulation of response to stimulus	GO:0048583	1.46E-11
regulation of response to stress	GO:0080134	1.71E-11
anatomical structure development	GO:0048856	2.30E-11
signal transduction	GO:0007165	3.35E-11
regulation of immune system process	GO:0002682	3.56E-11
positive regulation of developmental		GO:0051094	4.08E-11
response to endogenous stimulus	GO:0009719	4.62E-11
apoptotic process	GO:0006915	4.97E-11
cell communication	GO:0007154	6.27E-11
positive regulation of cytokine produc	GO:0001819	6.32E-11
regulation of cytokine production	GO:0001817	1.67E-10
cytokine production	GO:0001816	1.89E-10
cell differentiation	GO:0030154	2.55E-10
cellular developmental process	GO:0048869	2.57E-10
positive regulation of nitrogen compo	GO:0051173	2.67E-10
regulation of macromolecule metabol	GO:0060255	2.76E-10
innate immune response	GO:0045087	3.29E-10
hemopoiesis	GO:0030097	3.53E-10
negative regulation of biological proce	GO:0048519	3.73E-10
regulation of gene expression	GO:0010468	3.84E-10

signaling	GO:0023052	4.59E-10
cellular response to stimulus	GO:0051716	5.08E-10
regulation of cell development	GO:0060284	6.31E-10
regulation of programmed cell death	GO:0043067	6.35E-10
regulation of macromolecule biosynth	GO:0010556	8.21E-10
tube morphogenesis	GO:0035239	1.16E-09
regulation of response to biotic stimu	GO:0002831	1.37E-09
response to oxygen-containing compo	GO:1901700	1.40E-09
vasculature development	GO:0001944	1.40E-09
regulation of cellular biosynthetic pro	GO:0031326	1.66E-09
multicellular organismal process	GO:0032501	1.70E-09
leukocyte differentiation	GO:0002521	1.86E-09
regulation of biosynthetic process	GO:0009889	1.98E-09
regulation of cellular metabolic proce:	GO:0031323	2.55E-09
cell activation	GO:0001775	3.13E-09
response to stimulus	GO:0050896	3.16E-09
regulation of metabolic process	GO:0019222	3.78E-09
angiogenesis	GO:0001525	3.84E-09
negative regulation of cellular process	GO:0048523	4.13E-09
leukocyte activation	GO:0045321	4.15E-09
regulation of apoptotic process	GO:0042981	4.25E-09
circulatory system development	GO:0072359	5.50E-09
regulation of nitrogen compound met	GO:0051171	6.29E-09
epithelial cell proliferation	GO:0050673	6.92E-09
cellular response to cytokine stimulus	GO:0071345	7.92E-09
leukocyte proliferation	GO:0070661	8.90E-09
positive regulation of immune respon	GO:0050778	1.05E-08
positive regulation of protein metabo	GO:0051247	1.55E-08
regulation of primary metabolic proce	GO:0080090	1.65E-08
positive regulation of response to ext	GO:0032103	1.69E-08
cell development	GO:0048468	1.81E-08
positive regulation of phosphorylatior	GO:0042327	1.88E-08
regulation of molecular function	GO:0065009	2.26E-08
cytokine-mediated signaling pathway	GO:0019221	2.40E-08
negative regulation of immune systen	GO:0002683	2.47E-08
blood vessel morphogenesis	GO:0048514	3.14E-08
positive regulation of cell population	GO:0008284	3.39E-08
regulation of innate immune response	GO:0045088	4.77E-08
lymphocyte proliferation	GO:0046651	5.03E-08
mononuclear cell proliferation	GO:0032943	6.41E-08
regulation of phosphorylation	GO:0042325	6.48E-08
positive regulation of phosphorus met	GO:0010562	7.11E-08
positive regulation of phosphate meta	GO:0045937	7.11E-08
positive regulation of cell migration	GO:0030335	7.76E-08
anatomical structure morphogenesis	GO:0009653	8.22E-08
positive regulation of protein phosphc	GO:0001934	8.29E-08
regulation of signal transduction	GO:0009966	9.32E-08

response to virus	GO:0009615	1.01E-07
regulation of leukocyte mediated imm	GO:0002703	1.20E-07
defense response to virus	GO:0051607	1.37E-07
positive regulation of cell motility	GO:2000147	1.40E-07
defense response to symbiont	GO:0140546	1.41E-07
regulation of catalytic activity	GO:0050790	1.65E-07
positive regulation of cell differentiati	GO:0045597	1.69E-07
regulation of protein phosphorylation	GO:0001932	1.73E-07
blood vessel development	GO:0001568	1.82E-07
positive regulation of defense respons	GO:0031349	1.90E-07
positive regulation of locomotion	GO:0040017	1.94E-07
regulation of anatomical structure mo	GO:0022603	2.16E-07
negative regulation of cell population	GO:0008285	2.16E-07
positive regulation of immune effecto	GO:0002699	2.52E-07
regulation of leukocyte proliferation	GO:0070663	2.81E-07
negative regulation of defense respon	GO:0031348	4.04E-07
negative regulation of programmed ce	GO:0043069	4.17E-07
regulation of phosphate metabolic pro	GO:0019220	4.30E-07
regulation of phosphorus metabolic p	GO:0051174	4.35E-07
regulation of inflammatory response	GO:0050727	4.53E-07
regulation of immune effector process	GO:0002697	5.21E-07
negative regulation of developmental	GO:0051093	5.24E-07
regulation of cell migration	GO:0030334	5.65E-07
immune effector process	GO:0002252	5.73E-07
negative regulation of multicellular or	GO:0051241	5.88E-07
leukocyte mediated immunity	GO:0002443	6.15E-07
protein phosphorylation	GO:0006468	6.43E-07
positive regulation of molecular funct	GO:0044093	6.94E-07
peptidyl-tyrosine phosphorylation	GO:0018108	7.25E-07
peptidyl-tyrosine modification	GO:0018212	7.75E-07
positive regulation of intracellular sigr	GO:1902533	1.07208E-06
regulation of cell motility	GO:2000145	1.34275E-06
inflammatory response	GO:0006954	1.34998E-06
animal organ development	GO:0048513	1.39088E-06
anatomical structure formation involv	GO:0048646	1.44528E-06
regulation of transferase activity	GO:0051338	1.5786E-06
T cell activation	GO:0042110	1.70281E-06
regulation of signaling	GO:0023051	1.71012E-06
positive regulation of nucleobase-con	GO:0045935	1.7135E-06
regulation of cell communication	GO:0010646	1.81077E-06
regulation of locomotion	GO:0040012	2.34813E-06
positive regulation of RNA metabolic p	GO:0051254	2.45259E-06
regulation of RNA biosynthetic proces	GO:2001141	2.55832E-06
negative regulation of response to stir	GO:0048585	2.89823E-06
positive regulation of transcription by	GO:0045944	3.47242E-06
positive regulation of protein modifica	GO:0031401	3.65497E-06
regulation of protein metabolic proce	GO:0051246	3.89218E-06

cellular response to endogenous stim	GO:0071495	3.99318E-06
cellular response to oxygen-containin	GO:1901701	4.57437E-06
response to lipid	GO:0033993	4.66199E-06
cell migration	GO:0016477	4.76255E-06
regulation of intracellular signal trans	GO:1902531	4.77637E-06
response to organic cyclic compound	GO:0014070	4.9331E-06
positive regulation of DNA-templated	GO:0045893	5.14987E-06
positive regulation of catalytic activity	GO:0043085	5.22429E-06
positive regulation of RNA biosyntheti	GO:1902680	5.49874E-06
RNA biosynthetic process	GO:0032774	5.98999E-06
response to nitrogen compound	GO:1901698	6.21438E-06
regulation of cell cycle	GO:0051726	6.77146E-06
regulation of cell adhesion	GO:0030155	7.00816E-06
phosphorylation	GO:0016310	7.19083E-06
lymphocyte activation	GO:0046649	7.4569E-06
regulation of DNA-templated transcri	GO:0006355	1.44666E-05
regulation of RNA metabolic process	GO:0051252	1.45147E-05
regulation of protein modification pro	GO:0031399	1.60263E-05
regulation of hemopoiesis	GO:1903706	1.8518E-05
intracellular signal transduction	GO:0035556	1.92739E-05
response to organonitrogen compoun	GO:0010243	2.39008E-05
response to hormone	GO:0009725	2.47495E-05
locomotion	GO:0040011	2.50252E-05
regulation of kinase activity	GO:0043549	2.6388E-05
negative regulation of response to ext	GO:0032102	2.91233E-05
DNA-templated transcription	GO:0006351	3.00454E-05
response to bacterium	GO:0009617	3.03932E-05
cell motility	GO:0048870	3.25526E-05
negative regulation of apoptotic proce	GO:0043066	3.38698E-05
positive regulation of cell developmer	GO:0010720	3.54427E-05
cellular response to stress	GO:0033554	3.70016E-05
gland development	GO:0048732	4.0303E-05
positive regulation of signal transduct	GO:0009967	5.44615E-05
regulation of biological process	GO:0050789	6.16218E-05
nucleobase-containing compound bio	GO:0034654	6.33953E-05
regulation of protein kinase activity	GO:0045859	6.52396E-05
immune response-regulating signaling	GO:0002764	6.61746E-05
regulation of nucleobase-containing c	GO:0019219	6.74457E-05
apoptotic signaling pathway	GO:0097190	7.33436E-05
positive regulation of MAPK cascade	GO:0043410	8.39299E-05
mononuclear cell differentiation	GO:1903131	8.72666E-05
heterocycle biosynthetic process	GO:0018130	9.20758E-05
aromatic compound biosynthetic proc	GO:0019438	9.73319E-05
regulation of cellular process	GO:0050794	0.000107287
positive regulation of apoptotic proce	GO:0043065	0.000150691
positive regulation of transferase acti	GO:0051347	0.000183761
regulation of transcription by RNA pol	GO:0006357	0.000196344

organic cyclic compound biosynthetic	GO:1901362	0.000198898
response to inorganic substance	GO:0010035	0.00021168
positive regulation of programmed ce	GO:0043068	0.00021542
epithelium development	GO:0060429	0.00022406
biological regulation	GO:0065007	0.000229642
cellular response to organic cyclic com	GO:0071407	0.000346831
positive regulation of cell communicat	GO:0010647	0.000346858
positive regulation of signaling	GO:0023056	0.000346858
transcription by RNA polymerase II	GO:0006366	0.000408265
gene expression	GO:0010467	0.00045775
regulation of leukocyte activation	GO:0002694	0.000527485
cellular nitrogen compound biosynthe	GO:0044271	0.00061718
cell-cell adhesion	GO:0098609	0.000639027
cell cycle	GO:0007049	0.000674912
cellular response to lipid	GO:0071396	0.000686278
transmembrane receptor protein tyro	GO:0007169	0.000822323
regulation of cell activation	GO:0050865	0.001134235
phosphate-containing compound met	GO:0006796	0.001161889
phosphorus metabolic process	GO:0006793	0.001342144
regulation of MAPK cascade	GO:0043408	0.001772187
negative regulation of cell differentiat	GO:0045596	0.001973968
macromolecule biosynthetic process	GO:0009059	0.002228717
negative regulation of signal transduc	GO:0009968	0.002278594
cell adhesion	GO:0007155	0.002504222
peptidyl-amino acid modification	GO:0018193	0.002733257
adaptive immune response	GO:0002250	0.003399317
negative regulation of cellular metabo	GO:0031324	0.004142551
negative regulation of signaling	GO:0023057	0.005763822
negative regulation of cell communica	GO:0010648	0.005763822
MAPK cascade	GO:0000165	0.006064052
enzyme-linked receptor protein signal	GO:0007167	0.00630383
negative regulation of macromolecule	GO:0010558	0.007608372
negative regulation of cellular biosynt	GO:0031327	0.010023484
negative regulation of biosynthetic pr	GO:0009890	0.010768513
tissue development	GO:0009888	0.014191853
negative regulation of metabolic proc	GO:0009892	0.019136454
protein metabolic process	GO:0019538	0.020052478
response to abiotic stimulus	GO:0009628	0.024250045
macromolecule modification	GO:0043412	0.025300269
cellular biosynthetic process	GO:0044249	0.026704217
mitotic cell cycle	GO:0000278	0.027054008
macromolecule metabolic process	GO:0043170	0.027689189
negative regulation of gene expressio	GO:0010629	0.032880826
organic substance biosynthetic proces	GO:1901576	0.033826353
biosynthetic process	GO:0009058	0.038638363
protein modification process	GO:0036211	0.040846913
immune system process	GO:0002376	9.65133E-05

10	cell surface receptor signaling pathwa	GO:0007166	0.000116405
	signal transduction	GO:0007165	0.011038934
	signaling	GO:0023052	0.024868947
	cell communication	GO:0007154	0.029274003
	response to stimulus	GO:0050896	0.049716927
