# Supplementary material for: Exploring molecular targets: herbal isolates in cervical cancer therapy
Source: Genomics Inform. 2024 Jun 26;22:9. doi: 10.1186/s44342-024-00008-1 (PMC11201312; doi:10.1186/s44342-024-00008-1)
Supplement: Supplementary file 6 — Additional file 6: Table S5. Molecular functions enriched in cervical cancer. [file 44342_2024_8_MOESM6_ESM.pdf]

| term_name | Term ID | FDR |
|---|---|---|
| binding | GO:0005488 | 1.42466E-06 |
| protein binding | GO:0005515 | 2.77E-39 |
| ion binding | GO:0043167 | 0.004356924 |
| catalytic activity | GO:0003824 | 0.002081875 |
| DNA binding | GO:0003677 | 3.83E-09 |
| small molecule binding | GO:0036094 | 1.36E-07 |
| anion binding | GO:0043168 | 5.95E-07 |
| identical protein binding | GO:0042802 | 7.24E-11 |
| carbohydrate derivative binding | GO:0097367 | 5.30E-08 |
| enzyme binding | GO:0019899 | 2.84E-11 |
| nucleotide binding | GO:0000166 | 2.57E-09 |
| nucleoside phosphate binding | GO:1901265 | 2.67E-09 |
| purine nucleotide binding | GO:0017076 | 1.64E-07 |
| molecular function regulator activity | GO:0098772 | 6.69829E-06 |
| ribonucleotide binding | GO:0032553 | 4.29E-07 |
| purine ribonucleoside triphosphate binding | GO:0035639 | 3.01E-08 |
| purine ribonucleotide binding | GO:0032555 | 4.12E-07 |
| protein-containing complex binding | GO:0044877 | 1.98E-08 |
| adenyl nucleotide binding | GO:0030554 | 3.20E-09 |
| ATP binding | GO:0005524 | 4.15E-10 |
| adenyl ribonucleotide binding | GO:0032559 | 7.80E-09 |
| signaling receptor binding | GO:0005102 | 6.76582E-06 |
| double-stranded DNA binding | GO:0003690 | 0.004592458 |
| sequence-specific DNA binding | GO:0043565 | 0.020101564 |
| sequence-specific double-stranded DNA bindin | GO:1990837 | 0.033406118 |
| molecular function activator activity | GO:0140677 | 1.11988E-05 |
| enzyme regulator activity | GO:0030234 | 0.022148486 |
| transferase activity, transferring phosphorus-cc | GO:0016772 | 7.09268E-06 |
| protein dimerization activity | GO:0046983 | 0.012089186 |
| cytoskeletal protein binding | GO:0008092 | 0.001377241 |
| kinase binding | GO:0019900 | 1.81E-08 |
| kinase activity | GO:0016301 | 2.35101E-06 |
| protein kinase binding | GO:0019901 | 6.51819E-06 |
| chromatin binding | GO:0003682 | 2.21E-08 |
| phosphotransferase activity, alcohol group as a | GO:0016773 | 0.000130562 |
| transcription factor binding | GO:0008134 | 7.37E-07 |
| catalytic activity, acting on a nucleic acid | GO:0140640 | 6.16895E-05 |
| protein homodimerization activity | GO:0042803 | 0.012687705 |
| protein kinase activity | GO:0004672 | 0.000161735 |
| ATP-dependent activity | GO:0140657 | 0.000744023 |
| DNA-binding transcription factor binding | GO:0140297 | 0.000142186 |
| molecular adaptor activity | GO:0060090 | 0.049453481 |
| signaling receptor regulator activity | GO:0030545 | 0.028982973 |
| catalytic activity, acting on DNA | GO:0140097 | 1.27E-12 |
| signaling receptor activator activity | GO:0030546 | 0.042595086 |
| DNA-binding transcription activator activity | GO:0001216 | 0.00931363 |

| | | |
|---|---|---|
| protein serine/threonine kinase activity | GO:0004674 | 0.001982285 |
| DNA-binding transcription activator activity, RN | GO:0001228 | 0.016004859 |
| protein serine kinase activity | GO:0106310 | 0.000207394 |
| tubulin binding | GO:0015631 | 0.001224248 |
| ATP hydrolysis activity | GO:0016887 | 0.002832271 |
| ubiquitin-like protein ligase binding | GO:0044389 | 0.001688526 |
| RNA polymerase II-specific DNA-binding transc | GO:0061629 | 0.024207735 |
| ubiquitin protein ligase binding | GO:0031625 | 0.002994205 |
| microtubule binding | GO:0008017 | 0.001821396 |
| kinase regulator activity | GO:0019207 | 0.00811892 |
| helicase activity | GO:0004386 | 4.1213E-06 |
| single-stranded DNA binding | GO:0003697 | 2.13E-08 |
| protein kinase regulator activity | GO:0019887 | 0.020284624 |
| ATP-dependent activity, acting on DNA | GO:0008094 | 1.75E-07 |
| growth factor activity | GO:0008083 | 0.0121685 |
| DNA helicase activity | GO:0003678 | 1.53E-09 |
| cytokine binding | GO:0019955 | 0.007005481 |
| transcription coregulator binding | GO:0001221 | 0.002900282 |
| damaged DNA binding | GO:0003684 | 8.30297E-06 |
| single-stranded DNA helicase activity | GO:0017116 | 5.78E-12 |
| cyclin-dependent protein serine/threonine kina | GO:0016538 | 0.000258889 |
| DNA secondary structure binding | GO:0000217 | 0.011973739 |
