# Supplementary material for: Exploring molecular targets: herbal isolates in cervical cancer therapy
Source: Genomics Inform. 2024 Jun 26;22:9. doi: 10.1186/s44342-024-00008-1 (PMC11201312; doi:10.1186/s44342-024-00008-1)
Supplement: Supplementary file 7 — Additional file 7: Table S6. Cellular components enriched in cervical cancer. [file 44342_2024_8_MOESM7_ESM.pdf]

term_name	term_id	adjusted_p_value
cytoplasm	GO:0005737	3.78E-36
chromosome	GO:0005694	6.34E-36
nucleoplasm	GO:0005654	3.32E-33
chromosomal region	GO:0098687	1.43E-29
condensed chromosome	GO:0000793	1.28E-28
chromosome, centromeric region	GO:0000775	2.05E-26
microtubule cytoskeleton	GO:0015630	2.51E-23
organelle lumen	GO:0043233	1.26E-21
intracellular organelle lumen	GO:0070013	1.26E-21
membrane-enclosed lumen	GO:0031974	1.26E-21
cytoskeleton	GO:0005856	2.23E-20
intracellular non-membrane-bounded organelle	GO:0043232	4.65E-20
non-membrane-bounded organelle	GO:0043228	4.81E-20
microtubule organizing center	GO:0005815	2.08E-18
nuclear lumen	GO:0031981	9.66E-18
condensed chromosome, centromeric region	GO:0000779	1.16E-17
cytosol	GO:0005829	1.47E-17
centrosome	GO:0005813	3.79E-17
spindle	GO:0005819	2.68E-16
kinetochore	GO:0000776	2.97E-16
nuclear chromosome	GO:0000228	5.05E-15
supramolecular complex	GO:0099080	3.35E-13
intracellular anatomical structure	GO:0005622	6.59E-13
intracellular organelle	GO:0043229	2.71E-12
midbody	GO:0030496	2.27E-11
organelle	GO:0043226	2.79E-11
membrane-bounded organelle	GO:0043227	2.88E-11
intracellular membrane-bounded organelle	GO:0043231	4.17E-11
protein-DNA complex	GO:0032993	5.06E-10
spindle pole	GO:0000922	1.34E-09
CMG complex	GO:0071162	1.98E-08
replication fork	GO:0005657	4.04E-08
chromatin	GO:0000785	1.43E-07
mitotic spindle	GO:0072686	1.86E-07
DNA replication preinitiation complex	GO:0031261	2.37E-07
condensed nuclear chromosome	GO:0000794	2.99E-07
endomembrane system	GO:0012505	4.01E-07
supramolecular polymer	GO:0099081	9.44E-07
supramolecular fiber	GO:0099512	1.35925E-06
extracellular space	GO:0005615	2.26824E-06
microtubule	GO:0005874	3.72043E-06
nuclear envelope	GO:0005635	3.8208E-06
polymeric cytoskeletal fiber	GO:0099513	3.84967E-06
nucleus	GO:0005634	4.79689E-06
extracellular region	GO:0005576	2.04933E-05
vesicle	GO:0031982	4.68768E-05

MCM complex	GO:0042555	5.14693E-05
transcription regulator complex	GO:0005667	7.63566E-05
side of membrane	GO:0098552	8.93644E-05
cell surface	GO:0009986	0.000100576
condensin complex	GO:0000796	0.000102647
vesicle lumen	GO:0031983	0.000190358
spindle midzone	GO:0051233	0.000233735
external side of plasma membrane	GO:0009897	0.000295245
cyclin-dependent protein kinase holoenzyme complex	GO:0000307	0.000414684
cytoplasmic vesicle lumen	GO:0060205	0.000484188
procentriole replication complex	GO:0120099	0.000495002
transferase complex, transferring phosphorus-containing	GO:0061695	0.000560217
membrane raft	GO:0045121	0.000574821
membrane microdomain	GO:0098857	0.00062145
nuclear periphery	GO:0034399	0.000788217
secretory granule lumen	GO:0034774	0.001043706
anchoring junction	GO:0070161	0.001250571
centriole	GO:0005814	0.001366145
ruffle	GO:0001726	0.001506974
chromosome, telomeric region	GO:0000781	0.00169163
perinuclear region of cytoplasm	GO:0048471	0.00170359
cell-cell junction	GO:0005911	0.001814591
RecQ family helicase-topoisomerase III complex	GO:0031422	0.001850842
spindle microtubule	GO:0005876	0.002325152
transferase complex	GO:1990234	0.002912449
nuclear matrix	GO:0016363	0.002934655
Ctf18 RFC-like complex	GO:0031390	0.004270804
outer kinetochore	GO:0000940	0.005657098
extracellular exosome	GO:0070062	0.005777217
nuclear membrane	GO:0031965	0.006039361
nuclear replication fork	GO:0043596	0.006828923
DNA polymerase complex	GO:0042575	0.008353912
nuclear body	GO:0016604	0.008574245
alpha DNA polymerase:primase complex	GO:0005658	0.008910959
phosphatidylinositol 3-kinase complex, class I	GO:0097651	0.009239625
phosphatidylinositol 3-kinase complex, class IA	GO:0005943	0.009239625
extracellular vesicle	GO:1903561	0.010086278
extracellular membrane-bounded organelle	GO:0065010	0.010318791
extracellular organelle	GO:0043230	0.010318791
phagocytic vesicle	GO:0045335	0.011942533
cytoplasmic vesicle	GO:0031410	0.012041617
intracellular vesicle	GO:0097708	0.013371525
secretory granule	GO:0030141	0.015392481
cell leading edge	GO:0031252	0.017107069
mitotic spindle pole	GO:0097431	0.018854859
phosphatidylinositol 3-kinase complex	GO:0005942	0.019893012
transcription repressor complex	GO:0017053	0.020339234

microtubule associated complex	GO:0005875	0.020359927
kinesin complex	GO:0005871	0.023555694
chromosome passenger complex	GO:0032133	0.025742872
cell periphery	GO:0071944	0.032164067
centralspindlin complex	GO:0097149	0.039882485
ribonuclease H2 complex	GO:0032299	0.039882485
membrane	GO:0016020	0.040277535
specific granule lumen	GO:0035580	0.041079208
replisome	GO:0030894	0.042226956
lumenal side of endoplasmic reticulum membrane	GO:0098553	0.042226956
extracellular matrix	GO:0031012	0.042972899
intercellular bridge	GO:0045171	0.043791762
plasma membrane region	GO:0098590	0.044303828
external encapsulating structure	GO:0030312	0.044815713
cytoplasm	GO:0005737	3.78E-36
chromosome	GO:0005694	6.34E-36
nucleoplasm	GO:0005654	3.32E-33
chromosomal region	GO:0098687	1.43E-29
condensed chromosome	GO:0000793	1.28E-28
chromosome, centromeric region	GO:0000775	2.05E-26
microtubule cytoskeleton	GO:0015630	2.51E-23
organelle lumen	GO:0043233	1.26E-21
intracellular organelle lumen	GO:0070013	1.26E-21
membrane-enclosed lumen	GO:0031974	1.26E-21
cytoskeleton	GO:0005856	2.23E-20
intracellular non-membrane-bounded organelle	GO:0043232	4.65E-20
non-membrane-bounded organelle	GO:0043228	4.81E-20
microtubule organizing center	GO:0005815	2.08E-18
nuclear lumen	GO:0031981	9.66E-18
condensed chromosome, centromeric region	GO:0000779	1.16E-17
cytosol	GO:0005829	1.47E-17
centrosome	GO:0005813	3.79E-17
spindle	GO:0005819	2.68E-16
kinetochore	GO:0000776	2.97E-16
nuclear chromosome	GO:0000228	5.05E-15
supramolecular complex	GO:0099080	3.35E-13
intracellular anatomical structure	GO:0005622	6.59E-13
intracellular organelle	GO:0043229	2.71E-12
midbody	GO:0030496	2.27E-11
organelle	GO:0043226	2.79E-11
membrane-bounded organelle	GO:0043227	2.88E-11
intracellular membrane-bounded organelle	GO:0043231	4.17E-11
protein-DNA complex	GO:0032993	5.06E-10
spindle pole	GO:0000922	1.34E-09
CMG complex	GO:0071162	1.98E-08
replication fork	GO:0005657	4.04E-08
chromatin	GO:0000785	1.43E-07

mitotic spindle	GO:0072686	1.86E-07
DNA replication preinitiation complex	GO:0031261	2.37E-07
condensed nuclear chromosome	GO:0000794	2.99E-07
endomembrane system	GO:0012505	4.01E-07
supramolecular polymer	GO:0099081	9.44E-07
supramolecular fiber	GO:0099512	1.35925E-06
extracellular space	GO:0005615	2.26824E-06
microtubule	GO:0005874	3.72043E-06
nuclear envelope	GO:0005635	3.8208E-06
polymeric cytoskeletal fiber	GO:0099513	3.84967E-06
nucleus	GO:0005634	4.79689E-06
extracellular region	GO:0005576	2.04933E-05
vesicle	GO:0031982	4.68768E-05
MCM complex	GO:0042555	5.14693E-05
transcription regulator complex	GO:0005667	7.63566E-05
side of membrane	GO:0098552	8.93644E-05
cell surface	GO:0009986	0.000100576
condensin complex	GO:0000796	0.000102647
vesicle lumen	GO:0031983	0.000190358
spindle midzone	GO:0051233	0.000233735
external side of plasma membrane	GO:0009897	0.000295245
cyclin-dependent protein kinase holoenzyme complex	GO:0000307	0.000414684
cytoplasmic vesicle lumen	GO:0060205	0.000484188
procentriole replication complex	GO:0120099	0.000495002
transferase complex, transferring phosphorus-containin	GO:0061695	0.000560217
membrane raft	GO:0045121	0.000574821
membrane microdomain	GO:0098857	0.00062145
nuclear periphery	GO:0034399	0.000788217
secretory granule lumen	GO:0034774	0.001043706
anchoring junction	GO:0070161	0.001250571
centriole	GO:0005814	0.001366145
ruffle	GO:0001726	0.001506974
chromosome, telomeric region	GO:0000781	0.00169163
perinuclear region of cytoplasm	GO:0048471	0.00170359
cell-cell junction	GO:0005911	0.001814591
RecQ family helicase-topoisomerase III complex	GO:0031422	0.001850842
spindle microtubule	GO:0005876	0.002325152
transferase complex	GO:1990234	0.002912449
nuclear matrix	GO:0016363	0.002934655
Ctf18 RFC-like complex	GO:0031390	0.004270804
outer kinetochore	GO:0000940	0.005657098
extracellular exosome	GO:0070062	0.005777217
nuclear membrane	GO:0031965	0.006039361
nuclear replication fork	GO:0043596	0.006828923
DNA polymerase complex	GO:0042575	0.008353912
nuclear body	GO:0016604	0.008574245
alpha DNA polymerase:primase complex	GO:0005658	0.008910959

phosphatidylinositol 3-kinase complex, class I	GO:0097651	0.009239625
phosphatidylinositol 3-kinase complex, class IA	GO:0005943	0.009239625
extracellular vesicle	GO:1903561	0.010086278
extracellular membrane-bounded organelle	GO:0065010	0.010318791
extracellular organelle	GO:0043230	0.010318791
phagocytic vesicle	GO:0045335	0.011942533
cytoplasmic vesicle	GO:0031410	0.012041617
intracellular vesicle	GO:0097708	0.013371525
secretory granule	GO:0030141	0.015392481
cell leading edge	GO:0031252	0.017107069
mitotic spindle pole	GO:0097431	0.018854859
phosphatidylinositol 3-kinase complex	GO:0005942	0.019893012
transcription repressor complex	GO:0017053	0.020339234
microtubule associated complex	GO:0005875	0.020359927
kinesin complex	GO:0005871	0.023555694
chromosome passenger complex	GO:0032133	0.025742872
cell periphery	GO:0071944	0.032164067
centralspindlin complex	GO:0097149	0.039882485
ribonuclease H2 complex	GO:0032299	0.039882485
membrane	GO:0016020	0.040277535
specific granule lumen	GO:0035580	0.041079208
replisome	GO:0030894	0.042226956
lumenal side of endoplasmic reticulum membrane	GO:0098553	0.042226956
extracellular matrix	GO:0031012	0.042972899
intercellular bridge	GO:0045171	0.043791762
plasma membrane region	GO:0098590	0.044303828
external encapsulating structure	GO:0030312	0.044815713
